# The DnaJK chaperone of *Bacillus subtilis* post-transcriptionally regulates gene expression through the YlxR(RnpM)/RNase P complex

**DOI:** 10.1128/mbio.04053-24

**Published:** 2025-02-11

**Authors:** Mitsuo Ogura, Yu Kanesaki, Hirofumi Yoshikawa, Koki Haga

**Affiliations:** 1 Institute of Oceanic Research and Development,Tokai University, Shizuoka, Japan; 2 Shizuoka Instrumental Analysis Center, Shizuoka University, Shizuoka, Japan; 3 Department of Bioscience, Tokyo University of Agriculture, Tokyo, Japan; 4 Institutete of Molecular and Cellular Biosciences, The University of Tokyo, Tokyo, Japan; The University of Kansas Medical Center, Kansas City, Kansas, USA

**Keywords:** chaperone, transcriptome, RNase P, DnaJK, YlxR(RnpM), mRNA metabolism, proline biosynthesis

## Abstract

To survive in harsh natural environments, translation and mRNA metabolism must be tightly and coordinately controlled, as saving biological costs increases fitness. However, the roles of protein chaperones in this control system are unclear. This study proposes the novel aspect of the link between translation and mRNA metabolism, that is, the co-translational DnaJK chaperone activity is involved in changes in mRNA metabolism by RNase P. We found that the expression of *proBA,* which encodes proline biosynthetic enzymes, is regulated by *ylxR*(*rnpM*) through the *proBA* promoter. YlxR(RnpM), which is associated with RNase P, was also involved in the posttranscriptional regulation of *proBA*. To clarify this posttranscriptional regulation, we screened transposon (Tn)-inserted mutants for cells with low *proB::lacZ* expression and identified the DnaJK chaperone as a regulator of *proB*. To explore the possibility that the complex of YlxR(RnpM) and RNase P might work with DnaJK, we performed an epistatic analysis using the *lacZ* fusions, which revealed that the regulation of *proB* by DnaJK/YlxR(RnpM)/RNase P, that is, co-translational chaperone activity, controlled mRNA metabolism. RNA sequencing analysis of cells deficient in the RNA component of RNase P (*rnpB*) revealed that 261 genes were upregulated in the *rnpB*::Tn strain. Among them, we identified *yoyD/yodF, besA*, and *epeXE,* which were also under the control of DnaJK/YlxR(RnpM)/RNase P regulatory cascade. Finally, we performed yeast two-hybrid analysis using DnaK as bait and identified two genes, *spoIVCA* and *nupG*, whose expression was post-transcriptionally regulated by DnaJK but independent of YlxR(RnpM). These results suggest a broader role for posttranscriptional gene regulation by DnaJK.

**IMPORTANCE:**

*Bacillus subtilis* lacking the DnaJK chaperone has not been reported to exhibit a distinct phenotype. However, our study revealed proline-dependent growth in a minimal medium in the *dnaJ*::Tn strain. Inhibition of *spoIVCA* expression in this strain was identified as a probable cause of the sporulation deficiency in previous and current studies using a single cell-level analysis. We also observed posttranscriptional regulation of *proBA* by the DnaJK and YlxR(RnpM)/RNase P complex. LacZ analyses of *proB::lacZ* in different backgrounds suggested that the above regulation ultimately functions in mRNA metabolism. In DnaJK-deficient cells, the nascent peptide may be misfolded, and if DnaJK chaperone activity is lost, such a signal may be transferred to RNase P. Therefore, *proBA* mRNA may be degraded in an RNase P-dependent manner if the misfolding of the polypeptide translated from this mRNA is detected. This system is useful for reducing the biological costs of futile mRNA elongation.

## INTRODUCTION

Translation and mRNA decay are tightly and coordinately controlled in both prokaryotes and eukaryotes (1–4). Because protein synthesis encounters many critical points and difficulties, in such cases, the translation process should be abandoned, and mRNA should be degraded to save valuable biological resources. For example, some nascent peptides tend to misfold. To avoid this issue, prokaryotes have evolved protein chaperone systems (5). The DnaJK chaperone with the GrpE cofactor is one such system and is highly conserved in both prokaryotes and eukaryotes (6–8). The ATP-dependent reaction cycle of DnaK is regulated by DnaJ, which presents substrate peptides to DnaK and the nucleotide exchange factor GrpE. ATP-bound DnaK has high protein-folding activity, and GrpE is involved in the ATP/ADP exchange of DnaK. In *Escherichia coli* and *Clostridium difficile,* DnaK is involved in heat tolerance, whereas in *Bacillus subtilis* DnaK is not distinctly involved in heat tolerance (growth defect was observed only at 53°C), and *dnaK* disruption shows negligible effects on many physiological processes (6, 9–11). However, the double mutant of *dnaK* and *tig*, which encodes the Trigger factor (TF), displays a range of phenotypic alterations, including a reduction in cell wall integrity (11). This finding is consistent with the role of TF as a ribosome-associated chaperone that initiates the folding of nascent peptides (12). As protein synthesis advances to the folding phase of the protein domains, TF is known to recruit DnaJ-binding to facilitate the proper folding of the domains (13).

The metabolism of mRNA starts with its transcription and ends with its degradation by RNases (14, 15). In *B. subtilis,* several RNases are involved in mRNA degradation; however, many issues remain unsolved (16). Briefly, the initial event of mRNA degradation is mRNA cleavage by the endoribonuclease RNase Y. Next, the 5′-exoribonuclease RNase J1 and the 3′-exoribonuclease polynucleotide phosphorylase (PNPase) play major roles in mRNA degradation. In the RNase Y-independent mechanism, RNase J1 is responsible for mRNA degradation. The other RNases play a role in the processing of tRNAs, leading to their maturation (15, 17). The 5′-end of pre-tRNA is cleaved by the endoribonuclease RNase P. In *B. subtilis,* several pre-tRNAs lack the CCA sequence, which is required for the binding of amino acids to tRNA. CCA-less pre-tRNAs are cleaved by the endoribonuclease RNase Z, and the CCA sequence is subsequently added. For the 3′-end of pre-tRNAs with the CCA sequence, several exoribonucleases are involved in their maturation, such as YhaM and PNPase. RNase P consists of the protein RnpA and the RNA RnpB (15). In *E. coli*, the gene for RNase P is essential; however, many tRNAs with immature 5′-ends can be efficiently aminoacylated (18). Therefore, the essentiality may be owing to global changes in mRNA metabolism for half of the mRNA in *E. coli* caused by the depletion of RNase P (19). RnpB of *B. subtilis* is specifically associated with YlxR (20). YlxR modulates RNase P activity *in vitro* and has been renamed RnpM. YlxR(RnpB) was initially identified as a component of the regulatory feedback-loop for glucose-induced gene expression (21–25). In this report, the term “YlxR(RnpM)” has been used because of its two biological activities, binding to RnpB and non-specific DNA binding, which would involve promoter regulation of several genes. RNA sequencing (RNA-seq) analysis has shown that the disruption of *ylxR*(*rnpM*) influences the expression of approximately 400 genes (22). These effects appear to be a combination of two different properties of YlxR(RnpM).

The expression of *proBA* encoding proline biosynthetic enzymes is controlled by *ylxR*(*rnpM*) (22, 26). This study revealed that YlxR(RnpM) affected both promoter activity and posttranscriptional regulation of *proB*. To further characterize this posttranscriptional regulation, we screened Tn-inserted mutants for low expression phenotypes of *proB::lacZ*, leading to the identification of the DnaJK chaperone as a regulator for *proB*. We explored the possibility that the complex of YlxR(RnpM) and RNase P might work with DnaJK and found such regulation, that is, the control of mRNA metabolism through co-translational chaperone activity. RNA-seq analysis of *rnpB*::Tn revealed that DnaJK/YlxR(RnpM)/RNase P regulated several genes in addition to *proBA*. Finally, we performed yeast two-hybrid analysis using DnaK as bait and identified two genes, *spoIVCA* and *nupG*, whose expression was also post-transcriptionally regulated by DnaJK.

## RESULTS

### Decreased glucose induction (GI) of the *proBA* promoter in the *ylxR*(*rnpM*) strain

**Fig 1:**
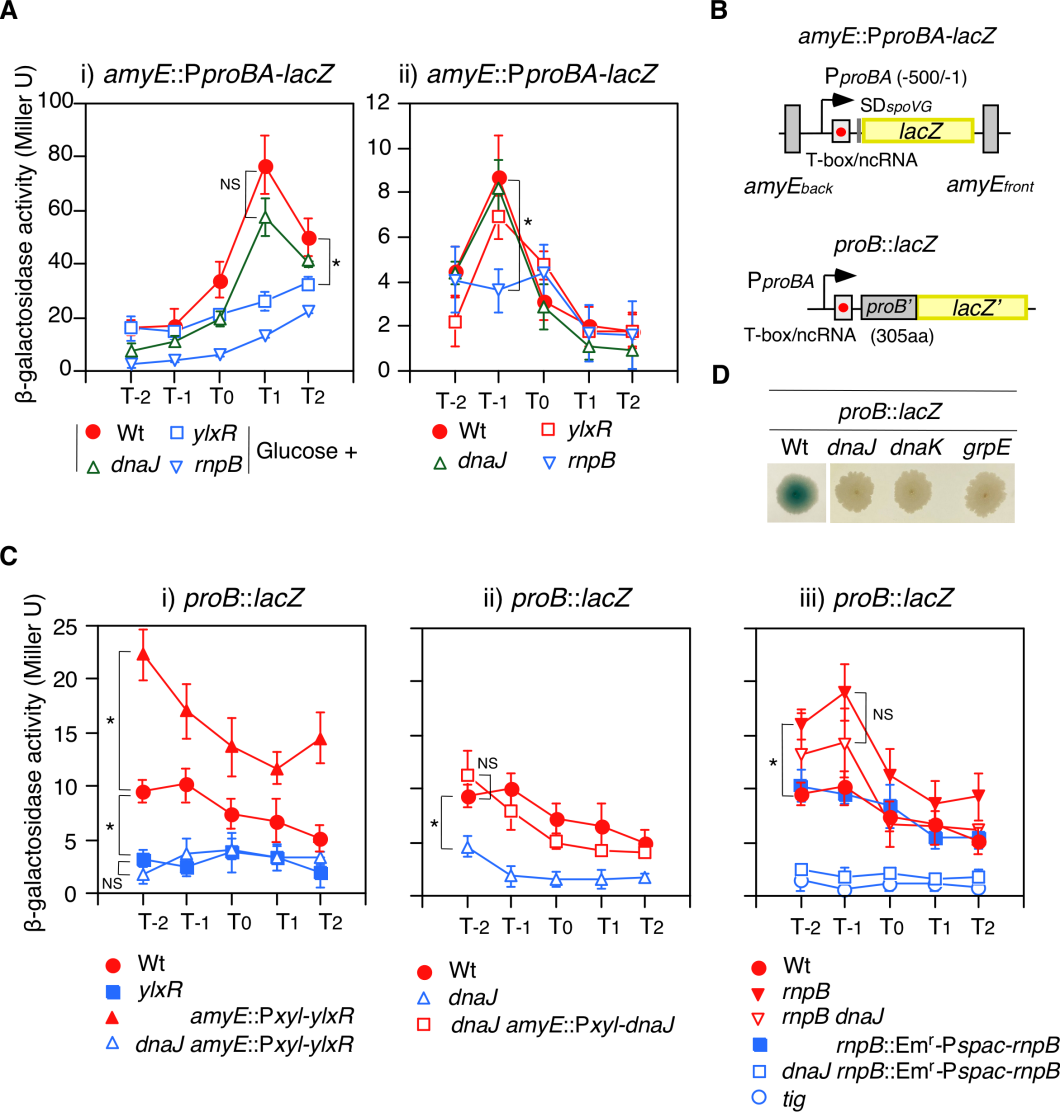
Expression of *proB*. β-Galactosidase activities are shown in Miller units. Data represent means ± standard deviations of three independent experiments. The *x*-axis represents the growth time in hours relative to the end of vegetative growth (T0). Cells were grown in SM. Significant differences for some data points were determined using nonpaired *t*-test and are shown in the panel. *P < 0.05; NS, not significant. (**A**) Expression of transcriptional P*proBA-lacZ* fusion in various genetic backgrounds. Substrate ONPG was used. (i) With 2% glucose. (ii) No glucose. OAM1163, wild-type; OAM1164, *ylxR*(*rnpM*); OAM1165, *dnaJ*; OAM1166, *rnpB*. (**B**) Schematic representations of fusion structure. Box and bent arrows show open reading frame or RNA gene, and promoter, respectively. Numbers in parenthesis indicate promoter region relative to the translation start site. aa, amino acid. The red circles show T-box. (**C**) Expression of translational *proB::lacZ* fusion in various genetic backgrounds. Substrate CPRG was used. (i) OAM821, wild-type; OAM822, *ylxR*(*rnpM*); OAM1139, Pxyl-*ylxR*; OAM1141, *dnaJ* Pxyl-*ylxR*. (ii) OAM821, wild-type; OAM1126, *dnaJ*; OAM1138, *dnaJ* Pxyl-*dnaJ*. (iii) OAM821, wild-type; OAM1136, *rnpB*; OAM1137, *rnpB dnaJ*; OAM1144, *rnpB*::Em^r^-Pspac-*rnpB*; OAM1145, *dnaJ rnpB*::Em^r^-Pspac-*rnpB*; OAM1220, *tig*. (**D**) Confirmation of Tn-screening. Cells carry *proB::lacZ* and the indicated Tn-insertion. The flesh cells from preculture on the LB agar plate containing appropriate antibiotics were inoculated to the SM agar plate containing appropriate antibiotics and 5-bromo-4-chloro-3-indolyl-β-D-galactoside (X-gal) and incubated 36 h at 42°C. OAM821, wild type; OAM1126, *dnaJ*; OAM1127, *dnaK*; OAM1128, *grpE*.

We identified YlxR(RnpM)-dependent GI of *proBA* mRNA using RNA-seq analysis (22). To confirm the effect of the *ylxR*(*rnpM*) disruption on the promoter activity, we constructed a P*proBA-lacZ* reporter system. The expression of P*proBA-lacZ* was induced by glucose, and its levels were considerably decreased in the *ylxR*(*rnpM*) strain (Fig. 1Ai). In the absence of glucose, *ylxR*(*rnpM*) disruption did not affect P*proBA-lacZ* (Fig. 1Aii). Therefore, the effect of *ylxR*(*rnpM*) on P*proBA* activity was limited to the GI.

### Posttranscriptional regulation of *proB*::*lacZ* by *ylxR*(*rnpM*)

YlxR(RnpM) operates at both transcriptional and posttranscriptional levels to regulate bimodal *frlB* gene expression without glucose (24). Thus, to explore the possible posttranscriptional regulation of *proB* by YlxR(RnpM) in the absence of glucose, we used the translational *proB::lacZ* fusion construct, which includes the first 305 codons of the *proB* open reading frame (ORF) (26) (Fig. 1B). This strategy was selected because the expression of *proB::lacZ* is expected to provide insights into both promoter activity and posttranscriptional regulation, owing to the inherent structural characteristics of the fusion construct. The partial ORF (1st–305th codons) contains a complete domain of ProB, which is composed of two domains according to the AlphaFold database (P39820). As *ylxR*(*rnpM*) disruption negatively affected *proB::lacZ* expression in the absence of glucose (Fig. 1Ci)*,* posttranscriptional regulation by *ylxR*(*rnpM*) may function through the mRNA encoding the partial *proB* ORF. The translational *proB::lacZ-2* fusion, which consists of the initial segment of the ORF (1st–68th codons) appeared to be unaffected by *ylxR*(*rnpM*) disruption (Fig. S1). This finding indicates that the synthesis of mRNA encoding the complete domain adjacent to the N-terminus is crucial for the regulation mediated by YlxR(RnpM).

### Identification of *dnaJK* and *grpE* as the posttranscriptional regulators of *proB*

To gain a deeper understanding of the posttranscriptional regulation of *proB*, we screened the Tn-inserted *B. subtilis* library carrying *proB::lacZ* for low-level *proB::lacZ* expression. In the specific mutant context, a pivotal gene involved in the posttranscriptional regulation of *proB* would be compromised, thereby facilitating the identification of that gene. We note that transposition was triggered by a temperature elevation from 30°C to 42°C (27). After screening approximately 12,000 colonies, nine loci were identified, including *cshA* and *ptsG*, both of which were isolated in a previous Tn-screen using *sigX-lacZ* (21, 28) (Table 1 and Fig. S2A). The *nusA* locus was isolated owing to the polar effect on *ylxR*(*rnpM*) that is downstream of *nusA* (data not shown). However, the rationale for isolating *pykA*, *xtmB*, and *flhB* remains unknown. The *dnaJK* and *grpE* loci were identified as mutants with low *proB::lacZ* expression (Fig. 1D). *dnaJ*::Tn was chosen as a representative of the DnaJK/GrpE chaperone system because *dnaJ* is the most downstream in *dnaJK/grpE* of the operon. As *dnaJ* is known to be heat-inducible and to counteract heat-induced protein aggregation (29), we investigated *proB::lacZ* expression and the effect of *dnaJ* disruption under three different thermal conditions. At 37°C, we observed the highest *proB* expression and the moderate effect of *dnaJ* disruption. In contrast, at 42°C, *proB* expression showed a decrease, but the highest impact of *dnaJ* disruption was observed (Fig. S2B). When the temperature was increased to 48°C, *proB* expression was abolished, but the underlying reasons remain elusive. Therefore, *dnaJ* experiments were performed at 42°C throughout this study, except for those related to sporulation.

**TABLE 1:** Transposon screening

Gene	Description	Tn-inserted sequence	Reproducibility (LacZ analysis) without glucose
*dnaK*	Molecular chaperone (Hsp70)	ACGTCACC	Observed (Fig. 1)
*dnaJ*	Molecular chaperone (Hsp40)	ACTTACTC	Observed (Fig. 1)
*grpE*	Activation of DnaK	CGTTAATT	Observed (Fig. 1)
		ATAGTTTT(2)^*a*^	Not tested
		ATAGCCTT	Not tested
		ATGGAATT	Not tested
*nusA*	Transcription termination factor	ATTCATCC	Observed (polar effect on downstream *ylxR*, data not shown)
*cshA*	DEAD-box RNA helicase	ATCCTGTC	Not tested
*ptsG*	Glucose permease	ACGACAAT	Not tested
*pykA*	Pyruvate kinase	ATGTGTGC	Observed (data not shown)
*xtmB*	PBSX terminase	ACGTCCAA	Not tested
*flhB*	Part of the flagellar type III export apparatus	ACTGCAGG(3)	Not tested

^*a*^ Numbers in parentheses indicate the number of independent Tn-insertions to the same site.

**Fig 2:**
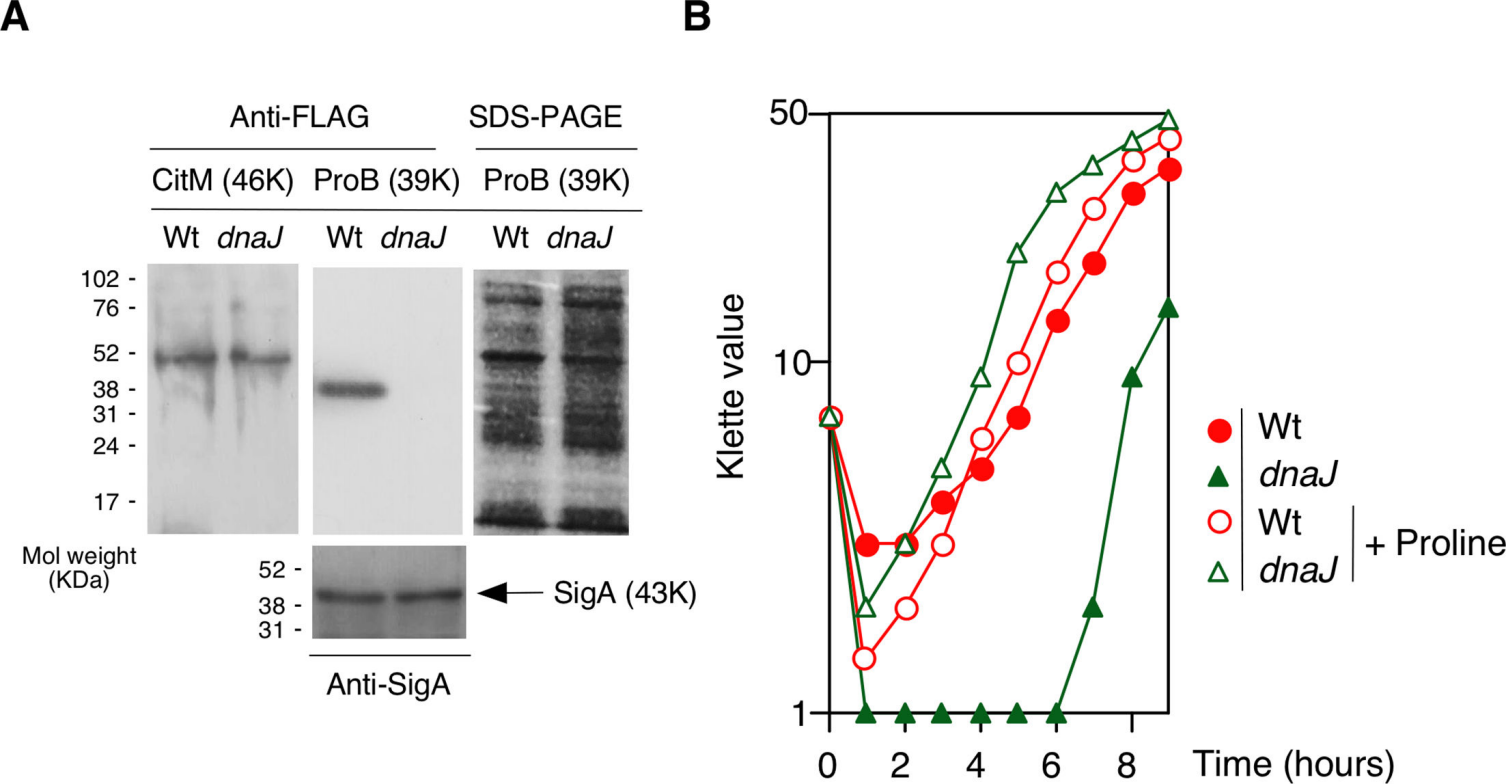
Characterization of *proB* mutant. (**A**) Western blot of ProB-FLAG (OAM1206) and CitM-FLAG (OAM1208). Their derivatives with *dnaJ* disruption were also analyzed (OAM1207 and OAM1209). (**B**) Cell growth profiles in various mutants. Typical cell growth profiles monitored with a Klett calorimeter (Fisher Scientific, Waltham, MA, USA) are shown. Overnight culture was grown in MC medium and inoculated to 4 mL Spizizen’s minimum medium in an L-tube. The final proline concentrations were 50 µg/mL. 168, wild type; OAM1124, *dnaJ*.

*dnaJ*::Tn severely decreased the amount of ProB-FLAG (Fig. 2A), although in the longer exposed membrane, a FLAG signal was detected. The *dnaJ*::Tn strain showed proline-dependent growth in the minimal medium (Fig. 2B). These results confirmed the depletion in ProB in the *dnaJ*::Tn strain. The *dnaJ*::Tn strain showed a decrease in *proB::lacZ* expression but not in P*proBA-lacZ* expression (Fig. 1A and Cii), suggesting that the effect of *dnaJ*::Tn requires a portion of the mRNA encoding the *proB* ORF. Probably, the folding of nascent peptides by DnaJK was involved in this regulation. We observed the complementation of the *dnaJ:*:Tn by an artificial expression of *dnaJ* from a xylose-inducible promoter (Fig. 1Cii), indicating that *dnaJ*::Tn was responsible for the decrease in *proB::lacZ* expression. The leaky xylose-inducible promoter (Pxyl) activity fulfilled the requirement of DnaJ for *proB::lacZ* expression. The TF encoded by *tig* is recognized as essential for DnaJ-binding to nascent peptides during the domain-folding stage (13). Consequently, we investigated whether *tig* is indispensable for *proB::lacZ* expression, and our findings corroborated this expectation (Fig. 1Ciii). The *tig* disruption did not affect the *proBA* promoter activity (data not shown). These demonstrate the functional chaperone network between TF and DnaJK for *proB* expression. Since *dnaJ,* in addition to *ylxR*(*rnpM*), post-transcriptionally affects *proB* expression, this suggests a regulatory link between *dnaJ*, *ylxR*(*rnpM*), and *rnpB,* which is a YlxR(RnpM)-associated RNA component of RNase P. Thus, we investigated the impact of the *rnpB* defect on *proBA* expression.

### Different regulations of the *proBA* promoter by *ylxR*(*rnpM*) and *rnpB*::Tn

We observed a decrease in P*proBA-lacZ* expression in the *rnpB*::Tn strain irrespective of glucose (Fig. 1A). The typical growth profile of the *rnpB*::Tn strain was similar to that of the wild-type strain (Fig. S2E). In our previous study, *rnpB*::Tn has been isolated as a mutant responsible for the decrease in *sigX-lacZ* GI (28) (Fig. S2A). Depletion of *rnpB* causes a stringent response, resulting in decreased expression of *pdhABCD*, which encodes pyruvate dehydrogenase (30, 31). This decrease was the cause of the loss of *proB* GI (Fig. S2A). Indeed, the *rnpB*::Tn strain exhibited decreased *pdhABCD* expression (Fig. S2C). *pdhABCD* expression, however, did not change in the *ylxR*(*rnpM*) strain, suggesting a mechanism for the regulation of P*proBA* by *ylxR*(*rnpM*) different from that in the *rnpB*::Tn strain. These results were obtained from RNA-seq analysis. Considering the non-specific DNA-binding activity of YlxR(RnpM) (22), this activity may function for *proBA* GI through the binding of YlxR(RnpM) to the *proBA* promoter (Fig. S2D).

### Identification of posttranscriptional regulation of *proB* by *rnpB*

Unlike P*proBA-lacZ*, *rnpB*::Tn increased *proB::lacZ* expression (Fig. 1Ciii)*,* suggesting posttranscriptional regulation by *rnpB*. The increased *proB::lacZ* expression in the *rnpB*::Tn strain was downregulated by the artificial expression of *rnpB* by the Pspac promoter (Fig. 1Ciii), indicating that *rnpB* was responsible for an increase in *proB::lacZ* expression. The leaky IPTG-inducible promoter activity in the absence of IPTG fulfilled the requirement of RnpB for *proB::lacZ* expression. Therefore, it was concluded that RNase P negatively affected *proB* expression in a direct or indirect manner. Posttranscriptional regulation at the mRNA level includes translation initiation, regulation by an anti-terminator located upstream of the ORF, and mRNA turnover (32). To discriminate these modes, we constructed *proB::lacZ-1* composed of the Shine-Dalgarno sequence and N-terminal two codons of *proB* fused to *lacZ* (Fig. S1). Therefore, translation initiation of this fusion was processed by *proB*-own sequence. The disruptions of *ylxR*(*rnpM*), *dnaJ*, and *rnpB* did not essentially affect *proB::lacZ-1* expression. These results strongly suggested that the final regulatory points of *ylxR*(*rnpM*), *dnaJ*, and *rnpB* lay at the *proBA* mRNA metabolism. This posttranscriptional regulation was evident in the *proB::lacZ* construct but not in the *proB::lacZ-2* variant, which features a truncated partial ORF (1st–68th codons) (Fig. S1).

### Analyses of linkage between *dnaJ, ylxR*(*rnpM*)*,* and *rnpB*

**Fig 3:**
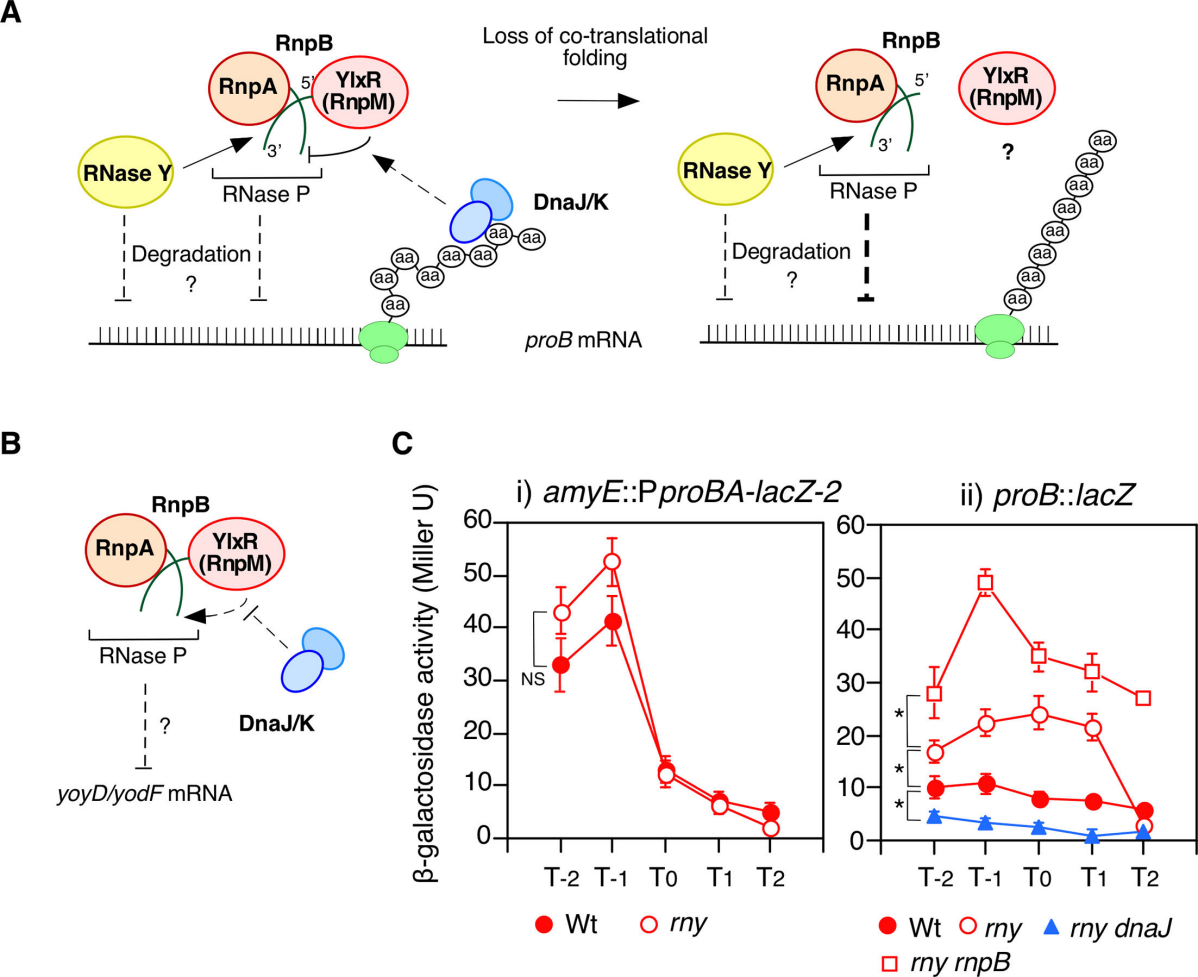
Effect of RNases on *proBA*. (**A** and **B**) Schematic model for linkage of DnaJK, RNase P/YlxR(RnpM), and RNase Y (A, *proBA*; B, *yoyD/yodF*). Arrows and T-bars indicate activation and inhibition, respectively. Dotted arrow and T-bar indicate effects that are based on circumstantial evidence. Green ovals show ribosome sub-units. Association of YlxR(RnpM) with RnpB and inhibition of RNase P by YlxR(RnpM) are reported (20). RNase Y has been reported to be responsible for 3′-end maturation of RnpB (33). As to mRNA degradation by RNase P neither its denial nor affirmation is in direct evidence. (**C**) β-Galactosidase activities are shown in Miller units. Data represents means ± standard deviations of three independent experiments. The *x*-axis represents the growth time in hours relative to the end of vegetative growth (T0). Cells were grown in SM. Significant differences for some data points were determined using nonpaired *t*-test and are shown in the panel. *P < 0.05; NS, not significant. Substrate CPRG was used. (i) *amyE*::P*proBA-lacZ*-2 has essentially the same structure as that of *amyE*::P*proBA-lacZ* except for antibiotic marker. OAM1167, wild-type; OAM1168, *rny*. (ii) OAM821, wild-type; OAM1140, *rny*; OAM1142, *rny rnpB*; OAM1143, *rny dnaJ*.

Since the disruption of *ylxR*(*rnpM*) decreased *proB::lacZ* expression, we expected that *ylxR*(*rnpM*) overexpression by Pxyl would increase *proB::lacZ* expression and observed the results accordingly (Fig. 1Ci). The leaky Pxyl activity fulfilled the requirement of YlxR(RnpM) for increasing *proB::lacZ* expression. Furthermore, *proB::lacZ* overexpression by YlxR(RnpM) required intact *dnaJ* because *ylxR*(*rnpM*) overexpression did not cause an increase in *proB::lacZ* expression in the *dnaJ*::Tn strain. Considering that YlxR(RnpM) inhibits RNase P activity (20), DnaJ may counteract the negative effect of RNase P activity on *proBA* mRNA through YlxR(RnpM) (Fig. 3A). In that case, the increased *proB::lacZ* expression in the *rnpB*::Tn strain would not be altered by the introduction of *dnaJ*::Tn. Indeed, we observed the expected bypass of the negative effect of *dnaJ*::Tn by *rnpB*::Tn (Fig. 1Ciii). We also observed that artificial *rnpB* expression in this *dnaJ rnpB* double mutant abolished the increased *proB::lacZ* expression*,* confirming the role of *rnpB*. This observation indicated that *dnaJ* sustained normal *proBA* expression through the partial *proBA* mRNA and RNase P activity, which was negatively regulated by YlxR(RnpM) (Fig. 3A). Although RNase P plays a significant role in mRNA metabolism, mRNA cleavage by RNase P has not been directly evidenced (19). Therefore, we investigated the possible role of major RNase in *B. subtilis,* RNase Y, on mRNA turnover (16). In the RNase Y-depleted strain, *proB::lacZ* expression*,* but not P*proBA-lacZ* expression, was increased, suggesting that RNase Y was involved in *proBA* mRNA turnover (Fig. 3C). We investigated whether RNase Y was involved in DnaJK/YlxR(RnpM)-dependent *proBA* regulation. To do so, *proB::lacZ* expression was examined in the strain with *rny* depletion and *dnaJ*::Tn. Even after *rny* depletion, the strong inhibitory effect of *dnaJ*::Tn on *proB::lacZ* was observed (Fig. 3Cii), suggesting that RNase Y and DnaJK had mutually independent regulatory modes. Moreover, the strain with *rny*-depletion and *rnpB*::Tn showed an additive effect on *proB::lacZ* expression. This result supported the idea that the *dnaJ*/*ylxR*(*rnpM*)/*rnpB* regulatory cascade and RNase Y function independently in the posttranscriptional regulation of *proBA*. RNase Y is responsible for 3′-end maturation of RnpB (33). However, this does not play a major role in the *dnaJ*/*ylxR*(*rnpM*)/*rnpB* regulatory cascade, based on the epistatic analysis above.

### Decreased *proB* expression in the *dnaJ* strain with disruptions of genes encoding tRNA-modifying enzymes

**Fig 4:**
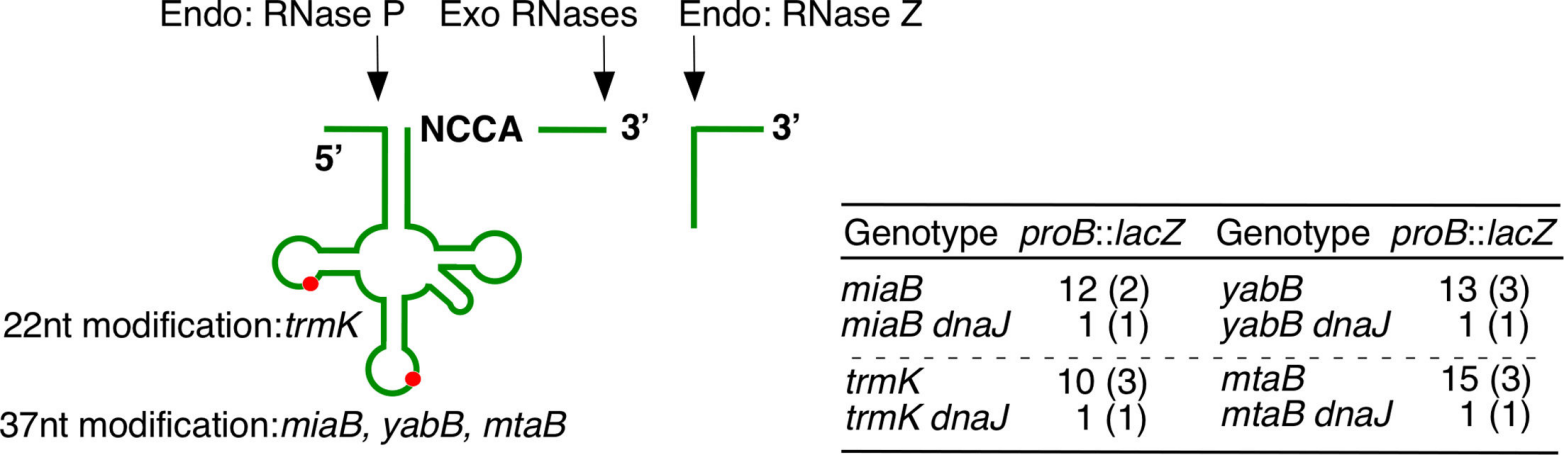
*proB* expression in the *dnaJ* strain with genes encoding tRNA-modifying enzymes. Left, schemes of tRNA maturation in *B. subtilis*. Exo, exoRNase; Endo, endoRNase. Two types of tRNA precursors are in *B. subtilis* cell, that is, tRNA with the CCA sequence and CCA-less tRNA. A part of the latter type tRNAs strictly requires RNase Z for processing. The red circle indicates the positions to be modified and the genes responsible for the modification of tRNA. Right, expression of *proB::lacZ* in the *dnaJ* and genes encoding tRNA modification enzyme. The numbers denote averages of the peak value and standard deviations are in the parenthesis. The values were obtained from at least three biologically independent experiments. In each experiment, the expression profile samples were taken at five points. OAM1151, *miaB*; OAM1152, *miaB dnaJ*; OAM1153, *yabB*; OAM1154, *yabB dnaJ*; OAM1155, *trmK*; OAM1156, *trmK dnaJ*; OAM1157, *mtaB*; OAM1158, *mtaB dnaJ*.

The tRNA-modifying enzyme *mtaB* is located downstream from *dnaJ* (34). To investigate the possible polar effects of *dnaJ*::Tn, the effect of *mtaB* disruption on *proB::lacZ* was observed. This disruption had no effect on *proB::lacZ,* whereas with *dnaJ*::Tn, *mtaB* disruption negatively affected *proB::lacZ* expression (Fig. 4). For P*proBA-lacZ,* double mutations had no effect (data not shown). Similar to this synergy, the other three disruptions of genes encoding tRNA-modifying enzymes showed the same effects (Fig. 4) (35). These effects are caused by a translation defect caused by immature tRNA. These observations were clearly different from the effects of *rnpB*::Tn, which also produces immature tRNAs. This suggested a role for RNase P other than tRNA maturation. The deficiency of some types of tRNA-modifying enzymes was evident in phenotypes of cell growth, perhaps through protein aggregation; however, the molecular mechanisms are unexplored (35). Therefore, these observations may help to elucidate such mechanisms.

### RNA-seq analyses of *rnpB* and *dnaJ*

**Fig 5:**
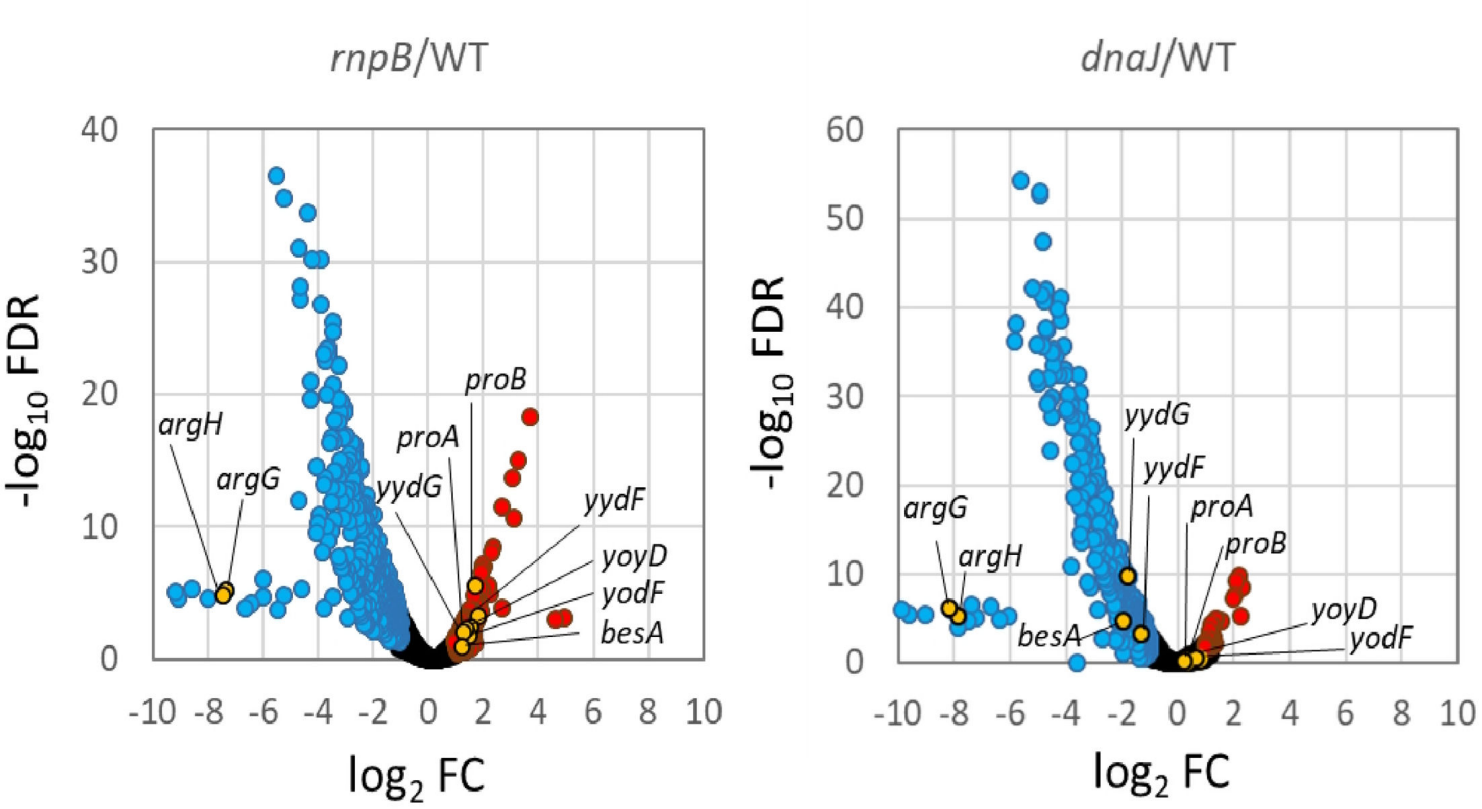
Volcano plot of the results of transcriptome analysis. Each dot represents an individual gene. The horizontal axis indicates log_2_ values of fold change expression level of each gene. Black dots represent genes with no significant change in mRNA level between the mutant and wild-type cells (WT). The blue and red dots represent downregulated and upregulated genes, respectively (|log_2_ FC| > 1; FDR < 0.05). Genes used in the detailed analysis in this study were indicated as orange dots. FDR; false discovery rate adjusted *p*P-value; FC, fold change.

The above analysis of *proB* expression raised two questions: whether *proBA* mRNA expression decreased in the *rnpB*::Tn strain and whether genes other than *proBA* are under *dnaJ/ylxR*(*rnpM*)*/rnpB* regulation. To address these questions, we performed comparative RNA-seq analyses of the *rnpB*::Tn and *dnaJ*::Tn strains using the late log phase cells grown in SM (Fig. 5 and Table S1). First, we focused on 261 genes with increased mRNA levels in the *rnpB*::Tn strain (Table S1). These included tRNA genes (21 of 86 tRNA genes in the genome), suggesting that *rnpB*::Tn altered the processing of tRNA for unknown reasons. In *B. subtilis*, little is known about the processing of polycistronic tRNA genes (15, 17). In a single polycistronic tRNA unit, some showed increased levels, which remains to be solved. In the *rnpB*::Tn strain, *proBA* mRNA levels increased, confirming that *proBA* was regulated by *rnpB* through mRNA metabolism. We used previous comparable RNA-seq results for the *ylxR*(*rnpM*) strain with glucose for further analysis (22). Among the genes with increased mRNA levels induced by *rnpB*::Tn, eight and seven genes were negatively affected by *dnaJ*::Tn and *ylxR*(*rnpM*)::Tn, respectively. Additionally, 31 genes were positively affected by *ylxR*(*rnpM*)::Tn. We tried to identify *proB*-type regulation (in *rnpB*, upregulation; in *dnaJ*, downregulation; in *ylxR*[*rnpM*], downregulation); however, such genes were not detected. Instead, different types of regulation (in *rnpB*, upregulation; in *dnaJ*, downregulation; in *ylxR*[*rnpM*], upregulation) were observed in five cases. When we focused on the 479 genes that showed decreased expression in the *rnpB*::Tn strain, 332 genes were concomitantly downregulated by *dnaJ* for unknown reasons (Table S1). From this category, we selected *argGH* for further analysis because of its strong downregulation by *rnpB*::Tn (Fig. 5; Fig. S3A). Using promoter fusion, the effects of *rnpB*::Tn and *dnaJ*::Tn, but not *ylxR*(*rnpM*)::Tn, were detected (Fig. S3B). These effects would be indirect because RnpB and DnaJ should not directly affect the promoter activity. Instead, *argGH* is regulated by the transcription factor AhrC, which may be the target of DnaJ or RnpB (36). In RNA-seq of *dnaJ*, 483 genes were downregulated, and 42 were upregulated (Fig. 5 and Table S1). Among the downregulated genes, 106 sporulation genes were detected. However, because the RNA samples were taken at the late log phase, the altered expression of many sporulation genes may not have a physiological role. Notably, the biofilm matrix operons *eps* (13 of total 15 ORFs) and *tasA/sipW* were downregulated. RNA-seq analysis of *dnaJ*::Tn showed levels of *proBA* mRNA similar to those in the wild-type.

### Expression analysis of genes identified by RNA-seq

**Fig 6:**
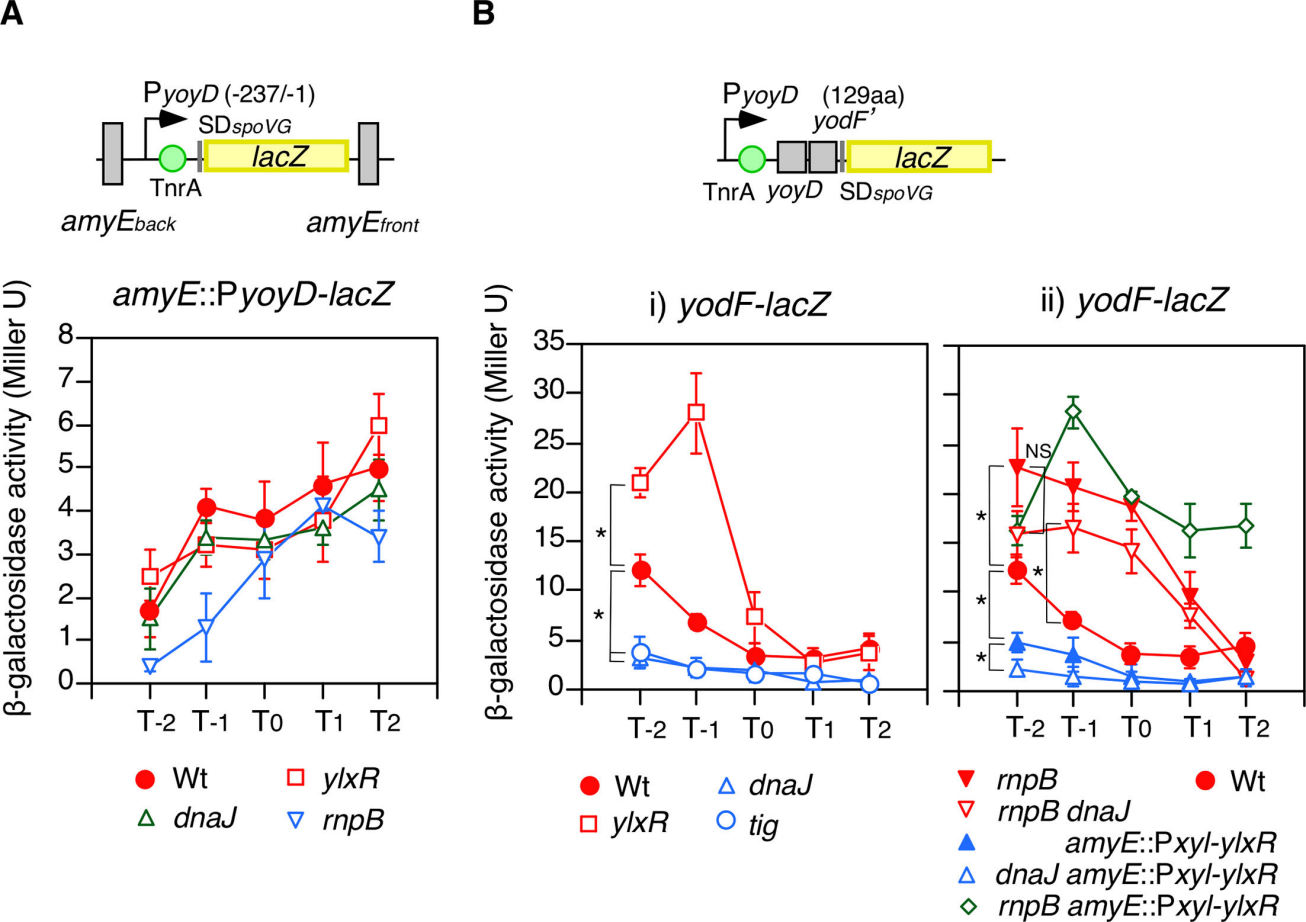
Expression of *yodF*. β-Galactosidase activities are shown in Miller units. Data represent means ± standard deviations of three independent experiments. The *x*-axis represents the growth time in hours relative to the end of vegetative growth (T0). Cells were grown in SM. Significant differences for some data points were determined using nonpaired *t*-test and are shown in the panel. *P < 0.05; NS, not significant. Substrate CPRG was used. Schematic representations of the fusion structure are shown above the panel. Box and bent arrows show open reading frame and promoter, respectively. Number in parenthesis indicates the promoter region relative to the translation start site. aa, amino acid. (**A**) Expression of transcriptional P*yoyD-lacZ* fusion in various genetic backgrounds. OAM1176, wild type; OAM1177, *ylxR(rnpM*); OAM1178, *dnaJ*; OAM1179, *rnpB*. (**B**) Expression of the transcriptional *yodF-lacZ* fusion in various genetic backgrounds. (i) YODFd, wild-type; OAM1169, *dnaJ*; OAM1170, *ylxR*(*rnpM*); OAM1224, *tig*. (ii) YODFd, wild-type; OAM1171, *rnpB*; OAM1172, *rnpB dnaJ*; OAM1173, Pxyl-*ylxR*, OAM1174, *dnaJ* Pxyl-*ylxR*; OAM1175, *rnpB* Pxyl-*ylxR*.

Based on the results of RNA-seq, the expression of several candidate genes using *lacZ* fusions was analyzed (Fig. S3A). *yoyD* and *yodF* encode a protein of unknown function and that is similar to a proline transporter, respectively (37). The promoter activity of *yoyD/yodF* was not altered in the *rnpB*, *ylxR*(*rnpM*), and *dnaJ* backgrounds, indicating no regulation of the promoter by these genes (Fig. 6A). In contrast, when using the transcriptional fusion with the entire *yoyD* ORF and partial *yodF* ORF (1st–129th codons), downregulation by *dnaJ*::Tn and *tig*, upregulation by *ylxR*(*rnpM*)::Tn, and upregulation by *rnpB*::Tn were observed (Fig. 6B). These results suggested that the regulation of *yoyD/yodF* was dependent on the *dnaJ*/*ylxR*(*rnpM*)/*rnpB* regulatory cascade. Notably, the regulatory vector between *ylxR*(*rnpM*) and *rnpB* should be opposite to that previously reported (20) (Fig. 3B). This suggested that an unknown molecule might modify the YlxR(RnpM) activity *in vivo*. The negative effect of *dnaJ*::Tn was bypassed by *rnpB*::Tn, indicating that the *dnaJ* target in this cascade was *rnpB* (Fig. 6Bii). Moreover, overexpression of *ylxR*(*rnpM*) resulted in a decrease in *yodF-lacZ* expression, and further introduction of *dnaJ*::Tn resulted in a further decrease (Fig. 6Bii). This indicated that DnaJK inhibited YlxR(RnpM). Finally, the introduction of *rnpB*::Tn into the strain overexpressing *ylxR*(*rnpM*) bypassed the decreasing effect of overexpressed *ylxR*(*rnpM*) (Fig. 6Bii). This strongly suggested that YlxR(RnpM) stimulated RnpB activity. These results were consistent with the scheme shown in Fig. 3B. We examined the effect of this regulatory cascade on candidate genes identified in RNA-seq analyses (Fig. S3A). One was monocistronic *besA* encoding ferri-bacillibactin esterase, which lacks a terminator downstream, resulting in readthrough transcription of *dhbACBEF* encoding proteins involved in enterobactin synthesis (38, 39). The other was the *epeXEP* operon, which controls the LiaSR two-component regulatory system; however, *epeP* did not meet our criteria (40) (Fig. S3A). The regulation of both operons was similar to that of *yoyD/yodF* according to the expression analyses using promoter fusion and promoter fusion with the partial ORF (1st–132th and 1st–114th codons for *besA* and *epeE*, respectively) (Fig. S3C and D).

### Yeast two-hybrid (Y2H) analyses using DnaK and GrpE as bait

The detailed analysis of *proBA* and RNA-seq showed that the DnaJK chaperone would directly regulate specific genes through mRNA encoding a part of or all of the ORF. To identify the genes subject to the *dnaJ/ylxR*[*rnpM*]/*rnpB* regulatory cascade, we adopted the Y2H system for determining interacting partner proteins with DnaK or GrpE. In *E. coli,* the DnaK interactome is very large (6); therefore, we adopted stringent screening conditions (supplemental methods). Fourteen and nine proteins were identified for DnaK and GrpE, respectively (Fig. S4A). This low number may reflect a weak interaction between DnaK and client proteins (41). Next, a FLAG tag was added to the C-terminus of the ORF of each gene, and protein levels in the wild and *dnaJ*::Tn backgrounds were analyzed by western blotting. The levels of SpoIVCA, ThiW, and NupG significantly decreased in the *dnaJ:*:Tn strain (Fig. S4B and data not shown for ThiW). Among them, the promoter activities of *spoIVCA* encoding a DNA recombinase for SigK and *nupG* encoding a purine nucleotide transporter were not affected by *dnaJ*::Tn (42, 43), whereas a decreasing effect of *dnaJ*::Tn was observed when *lacZ* was fused with partial ORF with N-terminus (25 and 132 codons for *spoIVCA* and *nupG*, respectively) (Fig. S4C and D). These results suggested that DnaJ positively regulated gene expression through its mRNA, which encoded partial ORF. However, *ylxR*(*rnpM*)::Tn showed no effect on either gene expression, indicating that *dnaJ* might function independently of YlxR(RnpM)/RNase P. We expected that mature SigK would not be generated in the *dnaJ*::Tn strain, resulting in inhibition of genes that are subject to regulators downstream of SigK in the sporulation sigma cascade (44). Four gene fusions were selected, and their expression was examined. The results matched our expectations (Fig. S4E). Microscopic assessments indicated a moderate reduction in sporulation efficiency in the *dnaJ* mutant cells (Fig. S4F). When counting the numbers of heat-resistant cells, indicative of spores, moderate reduction was similarly observed in sporulation efficiency at a low dilution factor (10^−6^). On the other hand, *dnaJ* cells exhibited a near-complete absence of spores at high dilution factors (10^−7^ and 10^−8^), in contrast to wild-type cells; however, viable cells were similarly absent in the *dnaJ* cell population, leading to an inflated estimation of apparent sporulation efficiency (Fig. S4F). Microscopic observations suggested that non-sporulating *dnaJ* cells apparently lost their ability to form colonies after 24 h of culture. These properties of *dnaJ* cells would previously have led to the assumption that sporulation was not abnormal (45). Our results are consistent with the findings using a single-cell-based approach that *dnaK*, *dnaJ*, and *grpE* are required for spore formation (46).

## DISCUSSION

We used a Tn-inserted library and the Y2H system to identify two phenotypes of the *dnaJ* disruptant: proline-dependent growth in minimal medium, and sporulation defect owing to the repression of *spoIVCA*.

A decrease in *proBA* mRNA was not observed in RNA-seq in *dnaJ*::Tn, although the decrease was observed in *proB::lacZ* analyses. This type of difference was also detected in the cases of *yoyD/yodF* and *nupG,* although a significant decrease in mRNA levels was observed for *besA* and *epeXE*. DnaJK acts as a chaperone for the nascent peptide from the transcribed mRNA, and the depletion of DnaJK results in peptide misfolding and concomitant stimulation of RNase P, possibly leading to mRNA turnover. Given this mode of action of DnaJK, which requires transcription and translation of a portion of the target gene, mRNA can be detected using RNA-seq analysis. Otherwise, the defect in *dnaJK* could result in the cleavage of mRNA, but not complete decay of mRNA. In this case, the remaining target mRNA can be detected by RNA-seq. In the case of *spoIVCA*, the RNA sample obtained in the late log phase did not contain mRNA derived from sporulation stage IV.

*rnpB* has been reported to be essential (47), whereas the *rnpB*::Tn strain used in this study was viable. The *rnpB*::Tn strain showed three characteristics of the *rnpB*-depleted strain. (i) tRNA processing was altered and this characteristic was observed in the *E. coli rnpB*-depleted strain (18, 19). (ii) In *B. subtilis,* the *rnpB*-depleted strain has been reported to show stringent phenotypes such as downregulation of *pdhABCD* and upregulation of *livBHCleuABCD* operons (31, 48). Our RNA-seq analysis also detected this phenotype (Table S1). Notably, the *rnpB*::Tn strain did not show any growth defects, probably resulting in no change in the *rrn* operons, whose expression is subject to a stringent response (49). (iii) The amount of *rnpB* RNA was depleted in the *rnpB*::Tn strain in RNA-seq. The reasons for deviation from the previous results on viability is unknown, although the use of a different medium may have contributed to the difference.

The link between translation and mRNA turnover has been extensively studied (1–4). However, the involvement of nascent protein folding in this linkage is, to our knowledge, unprecedent. Only one study has reported reduced mRNA stability in an *E. coli dnaK* mutant (50). The mechanism involves mRNA secondary structure that protects mRNA from RNase attack. Our study suggests that a defect in co-translational protein folding in the *dnaJ*::Tn strain causes RNase P-dependent alteration of mRNA metabolism through YlxR(RnpM). In the interactome of cyanobacterial YlxR(RnpM), DnaJ has been detected among the seven DnaJ homologs in the cyanobacterial genome (51). This observation raises the possibility that in *B. subtilis,* YlxR(RnpM) interacts with DnaJ, leading to the regulation of YlxR(RnpM) activity by DnaJK.

The half-life of mRNA transcribed from several genes is either upregulated or downregulated in an *rnpB*-depleted strain of *B. subtilis* (52). The genes analyzed in that study have not been obtained by genome-wide analysis, perhaps indicating the inadequate coverage of the inventory. The different gene set was observed in our RNA-seq, except for *bmrDE*(*yheIH*) (52). An immature tRNA-dependent mechanism has been proposed for the alteration of mRNA half-lives (Fig. S5). Immature tRNAs lead to ribosome stalling on mRNA, in the *rnpB-*depleted strain, where mRNA might be protected by stalled ribosomes from RNase Y. However, when *ylxR*(*rnpM*)::Tn causes RNase P activation, the immature tRNA hypothesis cannot explain the decrease in the *proBA* mRNA levels (22) because the target molecules of activated RNase P are unknown except for pre-tRNAs. Furthermore, based on this hypothesis, the expected increase in *proB::lacZ* expression in the *rny*-depleted strain should be similar to that observed in the *rny*-depleted *rnpB*::Tn strain. However, this was not the case in the present study. These observations do not support this hypothesis. The results of epistasis in this study can be easily explained by the hypothesis that RNase P catalyzes mRNA turnover. The RNase P dimer binds to the ribosomal 30S subunits, to which mRNA binds (53). Moreover, mRNA processing of several polycistronic operons of *E. coli* requires RNase P (54, 55). In eukaryotes, RNase P-MRP complex has been reported to target approximately 25% of mRNAs with one or more m^6^A internal modifications (56). These observations support the hypothesis that RNase P cleaves mRNA in *B. subtilis*. In RNase P-deficient *E. coli* cells, approximately 46% of mRNAs are altered (19), and in the *B. subtilis rnpB*::Tn strain, approximately 17% were changed, indicating a global effect of RNase P on mRNA metabolism, although in both bacteria, the molecular mechanism by which RNase P deficiency functions in mRNA metabolism is unknown.

Depletion of *rnpB* or *rnz* alters the mRNA metabolism of genes involved in ribosome biogenesis and generates immature tRNAs (30), and increased *proB::lacZ* expression was observed in the *rnz*-depleted strain (30 Miller units, unpublished results). To test the possibility that *rnpB*::Tn and *rnz* depletion are in the same regulatory cascade, we examined *proB::lacZ* expression in the *dnaJ*::Tn strain with *rnz*-depletion, and an additive effect of both mutations was observed (approximately 7 Miller units). Therefore, *rnpB* and *rnz* were involved in different regulatory cascades, that is, the effect of *rnz* depletion was probably caused by the immature tRNA-dependent mechanism shown in Fig. S5.

Finally, the RNA polymerase subunits, RpoA, RpoB, and RpoC were identified in the interactome of the cyanobacterium YlxR(RnpM) (51). This supports the hypothesis of transcriptional regulation by the promoter-bound YlxR(RnpM). Considering these, YlxR(RnpM) functions at two levels, that is, transcriptional and posttranscriptional regulation. This study clarifies the profile of *in vivo* roles of YlxR(RnpM) and highlights the need for further functional studies on this interesting protein, YlxR(RnpM).

## MATERIALS AND METHODS

### Strains, media, plasmid, and PCR

*B. subtilis* strains and plasmids in this study are listed in Table S2. The plasmids construction is provided in supplementary methods. A one-step competence medium (57), Schaeffer’s sporulation medium (SM) (58), Antibiotic III medium (Difco, MI, USA), and Spizizen’s minimal medium (59) were used. Antibiotic concentrations were described previously (60). Synthetic oligonucleotides were purchased from Tsukuba Oligo Service (Ibaraki, Japan) and are listed in Table S3. For PCR, PrimeSTAR MAX DNA polymerase (Takara Co., Shiga, Japan) was used.

### Transposon mutagenesis

The transposon delivery vector pMarA was introduced into OAM821 (Table S2) (27). The resultant strain was incubated in a liquid LB medium containing tetracycline and kanamycin at 30°C overnight. The cells were diluted and plated onto SM with 1.5% agar plates containing X-gal (100 µg/mL), kanamycin, tetracycline, and 2% glucose. The plates were incubated at 42°C, and white colonies were selected. The Tn-inserted mutations were backcrossed into OAM821 and subject to the Lac assay. Total DNA from the candidates was *Sau*IIIA1-digested, ligated, and amplified with inverse PCR using oligonucleotides 695 and 696, as described previously (61) (Table S3). The PCR products were sequenced using the oligonucleotide 696.

### β-Galactosidase analysis

Growth conditions and methods for β-galactosidase analysis have been previously described except for an incubation temperature 42°C (21). Growth was monitored and expressed relative to T0, the end of log phase (optical density at 600 nm = 1.5). The highly sensitive substrate chlorophenol red β-D-galactopyranoside (CPRG; Roche, Germany) and 2-nitrophenyl-β-D-galactopyranoside (ONPG; Wako-Fuji, Japan) were used for the β-galactosidase assay.

### Western blot analysis

Cells were grown in 50 mL SM at 42°C and harvested in the late log phase. Procedures of cell lysis, fractionation, and western blot were described previously (23, 62). Monoclonal mouse anti-FLAG M2 antibody (F3165) was purchased from Sigma-Aldrich (Darmstadt, Germany). Polyclonal rabbit anti-SigA antibodies and the detection of antigen were described previously (21, 23).

### RNA isolation and RNA-seq analysis

*B. subtilis* wild-type (168), *rnpB*::Tn (OAM1125), and *dnaJ*::Tn (OAM1124) strains were grown in 50 mL of SM at 42°C, and 4 mL of culture was sampled in the late log phase. Three independent cultures were used for each experiment. RNA isolation and removal of DNA were performed using an RNeasy mini kit (Qiagen, Germantown, MD, USA) according to the manufacturer’s instructions. RNA-seq was carried out by Novogene Inc. (Hong Kong, PRC). One hundred and fifty cycles of paired-end sequencing were carried out. To process the raw data, the Illumina package bcl2fastq was used. The RNA-seq reads were trimmed using CLC Genomics Workbench ver. 10.0.1 (Qiagen) with the following parameters; phred quality score >30; removing terminal 15 nucleotides from the 5′ end, and 2 nucleotides from the 3′ end; removing truncated reads less than 30 nucleotide length. Trimmed reads were mapped to all genes in *B. subtilis* 168 (accession number: NC_000964.1) using CLC Genomics Workbench ver. 10.0.1. with the following parameters; length fraction, 0.8; similarity fraction, 0.9; the maximum number of hits for a read, 1. The expression level of each gene was calculated by counting the mapped reads of each gene and normalized by calculating reads per million mapped reads values. .FDR <0.05 was considered statistically significant.

## Data Availability

Original sequence reads were deposited in the DRA/SRA database (accession numbers DRR590828–DRR590836). The raw data are available under bioproject number PRJDB18663.

## SUPPLEMENTAL MATERIAL

Supplemental material mbio.04053-24-s0001.pdf Supplemental methods, Tables S1-S3, and Fig. S1-S5.

ASM does not own the copyrights to Supplemental Material that may be linked to, or accessed through, an article. The authors have granted ASM a non-exclusive, world-wide license to publish the Supplemental Material files. Please contact the corresponding author directly for reuse.
