# Supplementary material for: The DnaJK chaperone of Bacillus subtilis post-transcriptionally regulates gene expression through the YlxR(RnpM)/RNase P complex
Source: mBio. 2025 Feb 11;16(3):e04053-24. doi: 10.1128/mbio.04053-24 (PMC11898665; doi:10.1128/mbio.04053-24)
Supplement: Supplemental material — Supplemental methods, Tables S1-S3, and Fig. S1-S5. [file mbio.04053-24-s0001.pdf]

The DnaJK chaperone of *Bacillus subtilis* post-transcriptionally regulates gene expression through the YlxR(RnpM)/RNase P complex

Mitsuo Ogura, Yu Kanesaki, Hirofumi Yoshikawa and Koki Haga

Supplementary Methods

Plasmid construction To construct pX-dnaJ, PCR products were amplified by using the oligonucleotide pair pX-dnaJ-Spe/pX-dnaJ-Bam, digested with SpeI/BamHI, and cloned into pX treated with the same enzymes (1). To construct pMutin-besA, PCR products were amplified by using the oligonucleotide pair pMut-besA-F-E/pMut-besA-R-B, digested with EcoRI/BamHI, and cloned into pMutinIII treated with the same enzymes (2). To construct pMUTIN-His-miaB, PCR products were amplified by using the oligonucleotide pair pUKM-miaB-F-E/pUKM-miaB-R-Xh, digested with EcoRI/XhoI, and cloned into pMutin-His treated with the same enzymes (3). To construct pIS284-proB, pIS284-yoyD, pIS284-besA, and pIS284-epeX, PCR products were amplified by using the oligonucleotide pairs pIS-proB-F-E/pIS-proB-R-B, pIS-yodF-F-E/pIS-yodF-R-B, pMut-besA-F-E/pIS-besA-R-B, and pIS-yydF-E/pIS-yydF-B, digested with EcoRI/BamHI, and cloned into pIS284 treated with the same enzymes (4). To construct pproB-flag, pcitM-flag, pspoIVCA-flag, and pnupG-flag, PCR products were amplified by using the oligonucleotide pairs Pflag-proB-F-E/Pflag-proB-R-Xb, Pflag-citM-F-E/Pflag-citM-R-Xb, Pflag-spoIVCA-F-E/Pflag-spoIVCA-R-Xb, and Pflag-nupG-F-E/Pflag-nupG-R-Xb, digested with EcoRI/XbaI, and cloned into pCA3xFLAG treated with the same enzymes (5). To construct pSPB107 and pSPB108, PCR products were amplified by using the oligonucleotide pairs pSK-ProB-E/pSK-ProB-H1 and pSK-ProB-E/pSK-ProB-H2, digested with EcoRI/HindIII, and cloned into pSK10D6-Tc treated with the same enzymes (6). To construct pUKM504-tig, PCR products were amplified by using the oligonucleotide pair pUKM-tig-F-E/pUKM-tig-R-Xh, digested with EcoRI/XhoI, and cloned into pUKM504 treated with the same enzymes (7). To construct pGBT9-dnaK and pGBT9-grpE, PCR products

were amplified by using the oligonucleotide pairs dnaK-5'/dnaK-3' and grpE-5'/grpE-3', digested with BamHI, and cloned into pGBT9 treated with the same enzyme.

Yeast two-hybrid analysis Yeast two-hybrid analysis for the library screening was performed according to the method described previously (8, 9). *Bacillus subtilis* genomic library for the screening was constructed using plasmids pGAD-C1, pGAD-C2, and pGAD-C3, and restriction enzymes AciI, HinPI, MspI, MaeII, and Taq1 (8). Yeast PJ69-4Aα was used for screening. The bait plasmid pGBT9-dnaK or pGBT9-grpE carries full-length *dnaK* or *grpE*. Diploid cells were grown on SC-LWHA plate containing 5 mM 3-aminotriazol and incubated for 7 days.

Microscopic observations 200 μl of the culture was centrifuged, and the cells were resuspended in 40 μl of LB medium. Portions (2 μl) of each sample were mounted on poly-L-lysine pre-coated glass slides. Microscopy was performed with an Olympus BX50 phase-contrast microscope with a 100× PlanN objective (Olympus, Tokyo, Japan). Images were captured using a SenSys (Photometrics, AZ, USA) charge-coupled device camera and Metamorph v7.6.5.0 software (Universal Imaging, PA, USA).

Sporulation test Cells were grown in 2 x SG medium at 37°C for 24 h. Serial dilutions of the cells were plated on LB agar plates before and after heating at 80°C for 10 min.

References to Supplementary Information

1. Hori K, Kaneko M, Tanji Y, Xing XH, Unno H. 2002. Construction of self-disruptive *Bacillus megaterium* in response to substrate exhaustion for polyhydroxybutyrate production. Appl Microbiol Biotechnol 59:211-216.
2. Vagner V, Dervyn E, Ehrlich SD. 1998. A vector for systematic gene inactivation in *Bacillus subtilis*. Microbiology 144:3097-3104.
3. Murayama S, Ishikawa S, Chumsakul O, Ogasawara N, Oshima T. 2015. The role of α-CTD in the genome-wide transcriptional regulation of the *Bacillus subtilis* cells. PLoS One 10:e0131588.

4. Tsukahara K, Ogura M. 2008. Promoter selectivity of the *Bacillus subtilis* response regulator DegU, a positive regulator of the *fla/che* operon and *sacB*. BMC Microbiol 8:8
5. Yamamoto H, Kurosawa S, Sekiguchi J. 2003. Localization of the vegetative cell wall hydrolases LytC, LytE, and LytF on the *Bacillus subtilis* cell surface and stability of these enzymes to cell wall-bound or extracellular proteases. J Bacteriol 185:6666-6677.
6. Ogura M, Kawata-Mukai M, Itaya M, Takio K, Tanaka T. 1994. Multiple copies of the *proB* gene enhance *degS*-dependent extracellular protease production in *Bacillus subtilis*. J Bacteriol 176:5673-5680.
7. Ogura, M, Tanaka T. 1996. Transcription of *Bacillus subtilis degR* is σ^D-dependent and suppressed by multicopy *proB* through σ^D. J Bacteriol 178:216-222.
8. James P, Halladay J, Craig EA. 1996. Genomic libraries and a host strain designed for highly efficient two-hybrid selection in yeast. Genetics 144:1425-1436.
9. Fukushima S, Yoshimura M, Chibazakura T, Sato T, Yoshikawa H. 2006. The putative ABC transporter YheH/YheI is involved in the signalling pathway that activates KinA during sporulation initiation. FEMS Microbiol Lett 256:90-97.
10. Ogura M, Kanesaki Y. 2018. Newly identified nucleoid-associated-like protein YlxR regulates metabolic gene expression in *Bacillus subtilis*. mSphere 3:e00501-18.
11. Ogura M. 2022. Identification of transposon-inserted mutations including *rnpB*::Tn that abolished glucose induction of *sigX* encoding extracytoplasmic function-sigma factor in *Bacillus subtilis.* Biosci Biotechnol Biochem 86:282-285.
12. Figaro S, Durand S, Gilet L, Cayet N, Sachse M, Condon C. 2013. *Bacillus subtilis* mutants with knockouts of the genes encoding ribonucleases RNase Y and RNase J1 are viable, with major defects in cell morphology, sporulation, and competence. J Bacteriol 195:2340-2348.
13. Kobayashi K, Ehrlich SD, Albertini A, Amati G, Andersen KK, *et al.*, 2003. Essential *Bacillus subtilis* genes. Proc Natl Acad Sci USA 100:4678-4683.
14. Ogura M, Matsutani M, Asai K, Suzuki M. 2023. Glucose controls

manganese homeostasis through transcription factors regulating known and newly identified manganese transporter genes in *Bacillus subtilis*. J Biol Chem 299:105069.
15. Sato T, Harada K, Ohta Y, Kobayashi Y. 1994. Expression of the *Bacillus subtilis spoIVCA* gene, which encodes a site-specific recombinase, depends on the *spoIIGB* product. J Bacteriol 176:935-937.
16. Stragier P, Bonamy C, Karmazyn-Campelli C. 1988. Processing of a sporulation sigma factor in *Bacillus subtilis*: how morphological structure could control gene expression. Cell 52:697-704.
17. Fajardo-Cavazos P, Tovar-Rojo F, Setlow P. 1991. Effect of promoter mutations and upstream deletions on the expression of genes coding for small, acid-soluble spore proteins of *Bacillus subtilis*. J Bacteriol 173:2011-2016.
18. Popham DL, Stragier P. 1991. Cloning, characterization, and expression of the *spoVB* gene of *Bacillus subtilis*. J Bacteriol 173:7942-7949.
19. González-Pastor JE, Hobbs EC, Losick R. 2003. Cannibalism by sporulating bacteria. Science 301:510-513.
20. Le Breton Y, Mohapatra NP, Haldenwang WG. 2006. In vivo random mutagenesis of *Bacillus subtilis* by use of TnYLB-1, a mariner-based transposon. Appl Environ Microbiol 72:327-333.
21. Delumeau O, Lecointe F, Muntel J, Guillot A, Guédon E, Monnet V. Hecker M, Becher D, Polard P, Noirot P. 2011. The dynamic protein partnership of RNA polymerase in *Bacillus subtilis*. Proteomics 11:2992-3001.
22. Ogura M, Asai K. 2016. Glucose induces ECF sigma factor genes, *sigX* and *sigM*, independent of cognate anti-sigma factors through acetylation of CshA in *Bacillus subtilis*. Front Microbiol 7:1918.
23. Ogura M, Sato T, Abe K. 2019. *Bacillus subtilis* YlxR, which is involved in glucose-responsive metabolic changes, regulates expression of *tsaD* for protein quality control of pyruvate dehydrogenase. Front Microbiol 10:923.
24. Trinquier A, Ulmer JE, Gilet L, Figaro S, Hammann P, Kuhn L, Braun F, Condon C. 2019. tRNA maturation defects lead to inhibition of rRNA processing via synthesis of pppGpp. Mol Cell 74:1227-1238.e3.

25. Trinquier A, Condon C, Braun F. 2023. Effect of tRNA Maturase depletion on levels and stabilities of ribosome assembly cofactor and other mRNAs in *Bacillus subtilis*. Microbiol Spectr 11:e0513422.

Table S1. RNA-seq of *rnpB* and *dnaJ*.
* RNA-seq for *ylxR* has been reported in Ref 10, where cells were grown in the same medium at 37C with 2% glucose. Cells for RNA-seq were sampled at T1.
# Sporulation-related genes are shown in red characters.

locus_tag	Region	Gene	Product	**Fold change (*rnpB*/WT)**	**Fold change (*dnaJ*/WT)**	*ylxR*/Wt up*	*ylxR*/Wt down
BSU28680	2933185..2934597	*glcD*	glycolate oxidase subunit	**31.161**	**1.11**		
BSU28690	2934594..2935928	*glcF*	glycolate oxidase iron-sulfur subunit	**24.606**	**1.13**		
BSU_tRNA_75	194205..194279	*trnSL-Glu2*	tRNA-Glu	**13.140**	**0.66**		
BSU_tRNA_32	951869..951944	*trnD-Val*	tRNA-Val	**9.741**	**0.52**		
BSU_tRNA_34	952042..952118	*trnD-Asp*	tRNA-Asp	**8.697**	**0.74**		
BSU_tRNA_78	194283..194358	*trnSL-Val1*	tRNA-Val	**8.219**	**0.78**		
BSU09280	1002501..1003325	*glpF*	glycerol permease	**6.447**	**1.48**		
BSU_tRNA_45	528704..528778	*trnS-Asn*	tRNA-Asn	**6.425**	**0.83**		
BSU_tRNA_33	951954..952030	*trnD-Met*	tRNA-Met	**5.219**	**0.66**		
BSU_tRNA_12	95375..95450	*trnJ-Val*	tRNA-Val	**5.032**	**1.16**		
BSU34850	complement(3581965..3582690)	*yvcA*	putative lipoprotein	**4.733**	**1.55**		
BSU02780	complement(299438..300502)	*ycdA*	lipoprotein involved in swarming behaviour	**4.615**	**1.64**		
BSU_tRNA_46	528783..528873	*trnS-Ser*	tRNA-Ser	**4.091**	**0.77**		
BSU18010	1929481..1929993	*yneN*	putative membrane-bound proteins with athioredoxin-like domain	**4.007**	**2.30**		
BSU_tRNA_47	528903..528974	*trnS-Glu*	tRNA-Glu	**3.977**	**1.15**		
BSU_tRNA_76	194363..194435	*trnSL-Thr1*	tRNA-Thr	**3.918**	**0.94**		
BSU24090	complement(2503765..2504664)	*ptb*	phosphate butyryl coenzyme A transferase	**3.873**	**2.41**		
BSU18000	1926680..1929409	*citB*	aconitate hydratase (aconitase)	**3.778**	**2.46**		
BSU_tRNA_37	952307..952391	*trnD-Tyr*	tRNA-Tyr	**3.720**	**0.87**		
BSU_tRNA_77	194458..194542	*trnSL-Tyr1*	tRNA-Tyr	**3.718**	**0.88**		
BSU33800	complement(3467546..3468235)	*opuCD*	glycine betaine/carnitine/choline/cholinesulfate ABC transporter (permease)	**3.496**	**0.90**		
BSU40180	complement(4127498..4127647)	*yydF*	peptide controlling LiaRS	**3.470**	**0.42**		
BSU05690	complement(614885..615622)	*ydhB*	putative integral inner membrane protein	**3.381**	**1.16**		
BSU04790	528129..528581	*ydcK*	conserved hypothetical protein	**3.359**	**1.14**		
BSU02140	complement(233994..235328)	*glpT*	glycerol-3-phosphate permease	**3.356**	**1.39**		
BSU_tRNA_35	952131..952206	*trnD-Phe*	tRNA-Phe	**3.325**	**0.94**		
BSU34840	complement(3580053..3581963)	*yvcB*	conserved hypothetical protein	**3.311**	**1.35**		
BSU29040	2968824..2970014	*ytbD*	putative transporter	**3.306**	**1.23**		
BSU08350	910019..910651	*estB*	secreted esterase / lipase	**3.270**	**2.15**		
BSU28260	complement(2889552..2890970)	*leuC*	3-isopropylmalate dehydratase (large subunit)	**3.249**	**1.93**		
BSU13120	1378496..1379593	*proB*	glutamate 5-kinase	**3.244**	**1.20**		
BSU34950	3590603..3591268	*pelC*	secreted pectate lyase	**3.230**	**0.97**		
BSU07340	complement(805456..806841)	*yfnA*	metabolite permease	**3.224**	**2.11**		
BSU32030	3294270..3294872	*bioYB*	putative biotin transporter	**3.209**	**1.36**		
BSU33820	complement(3469184..3469837)	*opuCB*	glycine betaine/carnitine/choline/cholinesulfate ABC transporter (permease)	**3.198**	**0.85**		
BSU02700	292205..292843	*estA*	secreted alkaliphilic lipase	**3.196**	**1.15**		
BSU12080	complement(1279514..1280503)	*ctaO*	minor protoheme IX farnesyltransferase 1 (heme Osynthase)	**3.196**	**0.91**		
BSU33330	complement(3419656..3421065)	*lysP*	lysine permease	**3.181**	**2.91**		
BSU23690	complement(2463571..2464041)	*yqjY*	putative acetyltransferase	**3.122**	**1.96**		
BSU_misc_RNA_44	complement(2869366..2869588)	*tboV*		**3.121**	**1.22**		
BSU19210	complement(2092899..2093762)	*yocH*	putative exported cell wall-binding protein	**3.114**	**1.87**		
BSU28250	complement(2888940..2889539)	*leuD*	3-isopropylmalate dehydratase (small subunit)	**3.087**	**1.86**		
BSU03070	complement(332441..333979)	*mdr*	multidrug-efflux transporter	**3.031**	**1.47**		
BSU31230	complement(3204067..3206055)	*tlpB*	methyl-accepting chemotaxis protein	**3.007**	**1.25**		
BSU09840	1057680..1059185	*hemZ*	coproporphyrinogen III oxidase	**3.003**	**1.69**		
BSU34830	complement(3579679..3580038)	*yvzA*	hypothetical protein	**2.998**	**1.29**		
BSU27370	complement(2795887..2796969)	*yrrL*	conserved hypothetical protein	**2.987**	**1.76**		
BSU24900	complement(2574408..2574557)	*rpmGA*	ribosomal protein L33	**2.970**	**1.49**		
BSU10570	complement(1130918..1132072)	*yhjN*	putative integral inner membrane protein	**2.967**	**1.29**		
BSU21330	2247889..2248335	*yomK*	hypothetical protein; phage SPbeta	**2.966**	**0.80**		
BSU19579	2130177..2130377	*yoyD*	putative exported protein	**2.963**	**1.60**		
BSU28270	complement(2891020..2892117)	*leuB*	3-isopropylmalate dehydrogenase	**2.961**	**1.57**		
BSU34010	complement(3489910..3491253)	*yvbW*	putative leucine permease	**2.876**	**1.06**		
BSU03580	complement(408240..408887)	*yczE*	integral inner membrane protein regulatingantibiotic production	**2.855**	**1.51**		
BSU19580	2130377..2131867	*yodF*	putative Na+/metabolite permease	**2.850**	**1.69**		
BSU28470	complement(2909520..2910746)	*lysC*	aspartokinase II alpha subunit (aa 1->408) andbeta subunit (aa 246->408)	**2.839**	**1.64**		
BSU33740	3463534..3464067	*yvaV*	putative transcriptional regulator	**2.805**	**1.44**		
BSU33750	3464289..3464765	*sdpA#*	export of killing factor	**2.803**	**0.24**		
BSU23210	complement(2425248..2425841)	*spcB*	chromosome condensation and segregation factor	**2.802**	**1.75**		
BSU39960	4104444..4104899	*yxaI*	putative integral inner membrane protein	**2.778**	**2.01**		
BSU_tRNA_36	952212..952284	*trnD-Thr*	tRNA-Thr	**2.771**	**1.01**		
BSU02400	complement(260123..261535)	*ybgF*	putative aminoacid permease	**2.736**	**1.19**		
BSU40830	complement(4193264..4194163)	*yyaK*	putative integral inner membrane protein	**2.714**	**1.16**		
BSU33810	complement(3468253..3469164)	*opuCC*	glycine betaine/carnitine/choline/cholinesulfate ABC transporter (osmoprotectant-bindinglipoprotein)	**2.707**	**0.69**		
BSU39480	complement(4058029..4058778)	*yxeO*	putative ABC transporter (ATP-binding protein)	**2.682**	**1.14**		
BSU38840	complement(3987927..3988763)	*yxkD*	efflux transporter	**2.677**	**2.16**		
BSU28310	complement(2895248..2896972)	*ilvB*	acetolactate synthase (acetohydroxy-acidsynthase) (large subunit)	**2.663**	**1.53**		
BSU39520	complement(4060818..4062143)	*yxeK*	putative monooxygenase	**2.658**	**1.42**		
BSU_tRNA_49	529087..529162	*trnS-Lys*	tRNA-Lys	**2.653**	**1.24**		
BSU39600	complement(4066607..4067005)	*yxeC*	putative integral inner membrane protein	**2.650**	**0.94**		
BSU15960	complement(1671166..1671651)	*ylqB*	conserved hypothetical protein	**2.646**	**0.68**		
BSU33760	3464762..3465733	*sdpB*	exporter of killing factor SpbC	**2.620**	**0.24**		
BSU15010	1569519..1570073	*rsmD*	putative ribosomal RNA small subunitmethyltransferase D	**2.619**	**1.77**		
BSU02130	complement(233014..233895)	*glpQA*	glycerophosphoryl diester phosphodiesterase	**2.617**	**1.36**		
BSU21610	2279977..2280867	*yokF*	SPbeta phage DNA nuclease, lipoprotein	**2.612**	**1.31**		
BSU03460	complement(375166..375798)	*hxlA*	3-hexulose-6-phosphate synthase (HPS)	**2.607**	**1.55**		
BSU07200	787992..788636	*yetJ*	putative integral membrane protein	**2.597**	**1.61**		
BSU_tRNA_48	528986..529060	*trnS-Gln*	tRNA-Gln	**2.592**	**0.77**		
BSU13130	1379605..1380852	*proA*	gamma-glutamyl phosphate reductase	**2.581**	**1.20**		
BSU03260	complement(351842..352696)	*ycgS*	putative aromatic hydrocarbon hydrolase	**2.575**	**1.07**		
BSU39490	complement(4058791..4059465)	*yxeN*	putative ABC transporter (permease)	**2.574**	**1.07**		
BSU07320	complement(803317..804546)	*yfnC*	putative efflux transporter	**2.570**	**1.05**		
BSU03450	complement(374603..375160)	*hxlB*	6-phospho-3-hexuloisomerase (PHI)	**2.564**	**0.74**		
BSU06250	679390..679761	*ydjM*	conserved hypothetical protein	**2.562**	**2.54**		
BSU35860	complement(3696257..3697498)	*pgdS*	gamma-DD-glutamyl hydrolase (PGA depolymerase)	**2.556**	**1.12**		
BSU39460	complement(4055536..4056873)	*yxeQ*	putative catabolic enzyme	**2.548**	**0.91**		
BSU40080	4117080..4118486	*gntZ*	NAD+-6-phosphogluconate dehydrogenase	**2.533**	**1.61**		
BSU33870	3473372..3474070	*yvbI*	putative permease	**2.530**	**1.09**		
BSU39510	complement(4060307..4060804)	*yxeL*	putative acetyltransferase	**2.528**	**1.11**		
BSU05680	613641..614849	*ydgK*	putative efflux transporter	**2.526**	**1.32**		
BSU37170	complement(3813246..3814385)	*acdA*	acyl-CoA dehydrogenase	**2.525**	**0.56**		
BSU28620	2926031..2926972	*rnhC*	ribonuclease HIII	**2.521**	**1.29**		
BSU13110	1377243..1378145	*purU*	formyltetrahydrofolate hydrolase	**2.511**	**1.39**		
BSU28280	complement(2892138..2893694)	*leuA*	2-isopropylmalate synthase	**2.509**	**1.40**		
BSU10230	complement(1098120..1098260)	*yhfH*	hypothetical protein	**2.488**	**1.34**		
BSU21020	complement(2221061..2221387)	*yonR*	putative transcriptional regulator (Xre family);phage SPbeta	**2.486**	**1.14**		
BSU_misc_RNA_40	complement(2800890..2801097)	*tboA*		**2.480**	**1.80**		
BSU39090	complement(4013795..4014475)	*yxiP*	putative lipoprotein	**2.479**	**1.05**		
BSU21540	complement(2272896..2273363)	*yolA*	putative exported protein; SPbeta phage	**2.478**	**1.30**		
BSU35190	3615793..3618288	*yvkC*	putative phosphotransferase	**2.475**	**0.62**		
BSU11055	complement(1183029..1183958)	*yitO*	putative integral inner membrane protein withHTTM domain	**2.475**	**0.69**		
BSU09290	1003344..1004834	*glpK*	glycerol kinase	**2.475**	**0.78**		
BSU29900	complement(3059547..3060188)	*trmB*	tRNA (guanine-N(7)-)-methyltransferase	**2.474**	**1.56**		
BSU02980	321013..322269	*opuAA*	glycine betaine ABC transporter (ATP-bindingprotein)	**2.473**	**1.17**		
BSU12180	1289298..1289939	*yjhA*	putative lipoprotein	**2.471**	**1.65**		
BSU16060	1677451..1678218	*rnhB*	ribonuclease HII	**2.459**	**1.21**		
BSU23680	complement(2463217..2463561)	*yqjZ*	putative degradation enzyme (oxygenase)	**2.456**	**1.66**		
BSU09130	986986..988377	*tcyP*	sodium-cystine symporter	**2.450**	**1.30**		
BSU_misc_RNA_51	3179105..3179206	*tswC*		**2.442**	**1.85**		
BSU24070	complement(2501549..2502640)	*buk*	branched-chain fatty-acid kinase	**2.440**	**1.75**		
BSU21340	2248417..2249100	*yomJ*	putative phage immunity protein; phage SPbeta	**2.435**	**0.91**		
BSU26620	complement(2720687..2721652)	*yrdR*	putative efflux transporter	**2.421**	**1.27**		
BSU38050	complement(3903646..3905031)	*sacP*	phosphotransferase system (PTS) sucrose-specificenzyme IIBC component	**2.414**	**1.24**		
BSU40170	complement(4126481..4127440)	*yydG*	putative AdoMet radical enzyme	**2.405**	**0.30**		
BSU34190	3510780..3512471	*yvfH*	putative lactate permease	**2.404**	**1.79**		
BSU12190	1290018..1290644	*yjhB*	putative ADP-ribose pyrophosphatase	**2.404**	**0.91**		

BSU40060	4114141..4115682	*gntK*	gluconate kinase	2.363	1.49
BSU39470	complement(4056870..4058012)	*yxeP*	putative amidohydrolase	2.356	1.00
BSU29600	3028297..3029634	*braB*	branched-chain amino acid-Na+ symporter	2.353	1.79
BSU35680	complement(3667209..3669911)	*ggaB*	poly(glucosyl N-acetylgalactosamine 1-phosphate)glucosyltransferase	2.349	1.56
BSU27410	complement(2798174..2800810)	*alaS*	alanyl-tRNA synthetase	2.340	1.44
BSU17910	1922549..1922767	*yneF*	conserved hypothetical protein	2.337	1.87
BSU33840	3471266..3471823	*yvbF*	putative transcriptional regulator	2.333	1.09
BSU24570	complement(2548245..2549333)	*gcvT*	aminomethyltransferase (glycine cleavage systemprotein T)	2.332	1.65
BSU07380	811569..812015	*yfmQ*	conserved hypothetical protein	2.324	1.41
BSU01580	177083..178519	*ybaR*	putative permease	2.321	1.57
BSU_misc_RNA_48	complement(2961232..2961479)	*tboT*		2.316	1.75
BSU38850	3989331..3989873	*yxkC*	conserved hypothetical protein	2.311	1.12
BSU33310	complement(3417222..3418376)	*fhuB*	ferrichrome ABC transporter (permease)	2.309	0.76
BSU03440	complement(372771..374492)	*tlpC*	methyl-accepting chemotaxis protein	2.308	1.10
BSU13570	complement(1424767..1425546)	*mtnU*	ketoglutaramate omega-amidase	2.307	1.57
BSU27390	complement(2797399..2797815)	*yrrK*	putative Holliday junction resolvase	2.306	1.39
BSU09720	1047072..1049093	*yheH*	ABC transporter (ATP-binding protein) involvedin the signalling pathway that activates KinA duringsporulation initiation	2.305	0.50
BSU32010	complement(3292490..3293359)	*besA*	bacillibactin trilactone hydrolase	2.304	0.27
BSU13280	1394776..1395288	*ykoJ*	putative exported protein	2.300	1.12
BSU14630	complement(1534279..1535751)	*speA*	arginine decarboxylase	2.299	1.50
BSU40070	4115711..4117057	*gntP*	gluconate permease	2.297	1.46
BSU38550	3957391..3958482	*ilvK*	branched-chain amino acid aminotransferase	2.293	1.72
BSU15020	1570078..1570563	*coaD*	phosphopantetheine adenylyltransferase	2.292	1.78
BSU03790	complement(430623..431987)	*yclM*	aspartate kinase III	2.282	1.25
BSU28080	complement(2865312..2866604)	*folC*	folyl-polyglutamate synthase	2.281	1.55
BSU09710	1045318..1047075	*yheI*	ABC transporter (ATP-binding protein) involvedin the signalling pathway that activates KinA duringsporulation initiation	2.276	0.51
BSU28290	complement(2893681..2894709)	*ilvC*	acetohydroxy-acid isomeroreductase	2.274	1.73
BSU38540	3956492..3957250	*dltE*	putative oxidoreductase	2.273	1.46
BSU02720	293499..294575	*yccF*	conserved hypothetical protein	2.272	1.45
BSU35520	3647406..3648581	*yvhJ*	putative membrane bound transcriptionalregulator	2.270	1.78
BSU10370	complement(1111925..1112485)	*bioY*	biotin transporter	2.269	1.32
BSU18650	2034745..2035782	*pelB*	pectin lyase	2.264	1.15
BSU19170	2088257..2089234	*yocD*	putative carboxypeptidase	2.259	1.01
BSU19140	complement(2086070..2086606)	*yozB*	putative integral inner membrane protein	2.257	1.42
BSU_tRNA_64	complement(3172600..3172676)	*trnB-Met2*	tRNA-Met	2.256	0.94
BSU_tRNA_65	complement(3172512..3172588)	*trnB-Asp*	tRNA-Asp	2.255	0.88
BSU24080	complement(2502659..2503753)	*bcd*	branched-chain amino acid dehydrogenase	2.255	1.72
BSU15210	1590317..1591417	*spoVE*	factor for spore cortex peptidoglycan synthesis(stage V sporulation)	2.246	1.41
BSU08930	970135..970617	*trmL*	tRNA (cytidine(34)-2'-O)-methyltransferase TrmL	2.243	1.56
BSU34640	complement(3558940..3559515)	*yvdD*	conserved hypothetical protein	2.241	1.07
BSU18800	2048533..2049453	*penP*	beta-lactamase precursor	2.236	1.33
BSU08460	921472..922503	*yfhA*	iron(III) siderophore transport permease	2.229	0.79
BSU23380	complement(2436947..2438266)	*lysA*	diaminopimelate decarboxylase	2.229	1.14
BSU23860	complement(2480750..2482159)	*gndA*	NADP+-dependent 6-P-gluconate dehydrogenase	2.226	2.17
BSU27110	2768827..2769654	*yrhO*	putative transcriptional regulator	2.225	1.51
BSU22810	complement(2387854..2388588)	*yphF*	putative lipoprotein	2.221	0.99
BSU13030	1372035..1372553	*ykhA*	putative acyl-CoA hydrolase	2.219	1.47
BSU02120	231348..232967	*ybeC*	putative H+/amino acid transporter	2.218	2.22
BSU18849	complement(2055868..2056107)	*yozV*	putative phage protein	2.213	1.45
BSU13430	1409912..1410577	*ykoX*	putative integral inner membrane protein	2.210	1.17
BSU35690	complement(3670035..3671375)	*ggaA*	poly(glucosyl N-acetylgalactosamine 1-phosphate)glucosyltransferase	2.209	1.24
BSU23070	2411086..2412663	*serA*	3-phosphoglycerate dehydrogenase	2.206	1.74
BSU35630	complement(3660648..3662765)	*lytB*	modifier protein of major autolysin LytC	2.203	1.35
BSU28890	2953795..2954460	*yscB*	putative lipoprotein	2.201	0.68
BSU31250	complement(3208280..3210268)	*tlpA*	methyl-accepting chemotaxis protein	2.201	0.36
BSU27080	complement(2763025..2765832)	*levR*	transcriptional regulator (NifA/NtrC family)	2.200	1.40
BSU36300	complement(3739206..3739982)	*glcR*	transcriptional regulator (DeoR family)	2.200	1.07
BSU39450	complement(4054354..4055466)	*yxeR*	putative ethanolamine transporter	2.200	0.85
BSU31510	complement(3236422..3236979)	*yufK*	putative integral inner membrane protein	2.198	1.45
BSU33460	complement(3436849..3437457)	*yvgT*	putative integral inner membrane protein	2.197	1.64
BSU08720	943891..944364	*ygaF*	putative bacterioferritin comigratory protein;putative peroxiredoxin	2.195	1.43
BSU19090	complement(2082531..2083013)	*yobU*	putative effector of transcriptional regulator	2.191	0.85
BSU_misc_RNA_59	3946910..3947116	*tboYB*		2.191	0.98
BSU03000	323119..324000	*opuAC*	glycine betaine ABC transporter (glycinebetaine-binding lipoprotein)	2.189	0.85
BSU14180	1488973..1489683	*dapH*	tetrahydrodipicolinate N-acetyltransferase	2.187	1.68
BSU05080	553711..554475	*yddR*	putative metal-dependent hydrolase	2.184	1.28
BSU08710	complement(942449..943738)	*gsaB*	glutamate-1-semialdehyde aminotransferase, classIII aminotransferase	2.171	1.44
BSU21530	complement(2272534..2272890)	*yolB*	conserved hypothetical protein; phage SPbeta	2.168	1.17
BSU38410	3942234..3943613	*sacX*	negative regulator of SacY	2.168	1.69
BSU11910	complement(1263702..1264931)	*yjcM*	conserved hypothetical protein	2.160	0.92
BSU17880	1919459..1919692	*ynzC*	conserved hypothetical protein	2.159	1.60
BSU15450	1616744..1617208	*lspA*	signal peptidase II	2.156	1.69
BSU36890	complement(3788426..3789055)	*upp*	uracil phosphoribosyltransferase	2.149	1.60
BSU18880	2057801..2058460	*yobE*	putative phage protein	2.144	1.31
BSU38640	3966745..3967947	*yxlH*	putative efflux transporter	2.142	1.34
BSU23040	2409729..2409977	*fer*	ferredoxin	2.142	1.62
BSU23840	complement(2478006..2478929)	*rnz*	ribonuclease Z	2.141	1.50
BSU_misc_RNA_20	complement(1424527..1424683)	*mswC*		2.140	1.35
BSU38530	3955223..3956401	*dltD*	putative D-alanine esterase for lipoteichoicacid and wall teichoic acid synthesis	2.140	1.50
BSU16350	1704863..1705528	*fliP*	component of the flagellar export machinery	2.139	1.35
BSU03370	complement(367305..367985)	*yckA*	putative ABC transporter (permease)	2.128	0.83
BSU05060	complement(552052..552501)	*lrpB*	transcriptional regulator (Lrp/AsnC family)	2.128	1.36
BSU31580	3244770..3246116	*maeN*	Na+/malate symporter	2.126	1.52
BSU16420	1711736..1712809	*cheB*	methyl-accepting chemotaxis proteins(MCP)-glutamate methylesterase	2.117	1.42
BSU28300	complement(2894733..2895251)	*ilvH*	acetolactate synthase (acetohydroxy-acidsynthase) (small subunit)	2.116	1.25
BSU23850	2479156..2480625	*zwf*	glucose-6-phosphate 1-dehydrogenase	2.111	1.77
BSU34020	complement(3491655..3492689)	*yvbX*	putative epimerase modification ofpeptidoglycan	2.110	1.28
BSU24710	complement(2557673..2557969)	*comGC*	pilin-like component of the DNA transportmembrane platform	2.108	0.55
BSU31070	3186763..3187305	*yuaC*	putative transcriptional regulator	2.108	1.71
BSU31100	3189089..3190426	*ktrB*	potassium transporter ATPase	2.105	1.68
BSU34660	complement(3559979..3561571)	*yvdB*	putative anion transporter	2.101	1.26
BSU39420	complement(4051602..4052273)	*deoC*	deoxyribose-phosphate aldolase	2.100	1.25
BSU14640	1535936..1536202	*yktA*	conserved hypothetical protein	2.100	1.63
BSU27090	2766558..2767946	*aapA*	d-Serine/d-alanine/glycine permease	2.100	1.29
BSU_misc_RNA_17	1378233..1378443	*pswB*		2.099	1.32
BSU03430	complement(372154..372597)	*nucA*	endonuclease	2.094	1.30
BSU17800	1912953..1914593	*yndJ*	putative integral inner membrane protein	2.093	1.19
BSU02990	322271..323119	*opuAB*	glycine betaine ABC transporter (permease)	2.091	0.86
BSU19590	complement(2131902..2133302)	*ctpA*	carboxy-terminal processing protease	2.089	1.37
BSU28450	complement(2908129..2908737)	*sdhC*	succinate dehydrogenase (cytochrome b558subunit)	2.086	1.78
BSU03380	complement(367995..368858)	*yckB*	putative ABC transporter (binding lipoprotein)	2.084	0.87
BSU24550	complement(2545410..2546876)	*gcvPB*	glycine decarboxylase (subunit 2) (glycinecleavage system protein P)	2.082	1.40
BSU18640	complement(2033895..2034572)	*yoaK*	putative membrane protein	2.077	0.92
BSU22910	complement(2397019..2397672)	*ypfA*	putative cyclic diGMP binding protein	2.072	1.53
BSU14550	1525386..1526159	*ykrA*	putative hydrolase	2.071	2.29
BSU01820	204351..204890	*adaB*	O6-methylguanine-DNA methyltransferase	2.068	1.07
BSU38030	complement(3901868..3902116)	*ywdA*	hypothetical protein	2.068	1.43
BSU39610	4067183..4068148	*yxeB*	ABC transporter (ferrioxamine bindinglipoprotein)	2.065	0.35
BSU24060	complement(2500104..2501528)	*lpdV*	branched-chain alpha-keto acid dehydrogenase E3subunit (dihydrolipoamide dehydrogenase)	2.064	1.65
BSU27070	complement(2762395..2762835)	*levD*	phosphotransferase system (PTS)fructose-specific enzyme IIA component	2.064	1.19
BSU09660	complement(1040861..1041709)	*yheN*	putative polysaccharide deacetylase	2.063	1.07
BSU21620	2280881..2281363	*yokE*	conserved hypothetical protein; phage SPbeta	2.062	1.17
BSU07420	complement(814384..815940)	*yfmM*	putative ATP-binding protein	2.057	1.56
BSU31620	3249427..3249768	*mrpC*	component of Na+/H+ antiporter	2.055	1.04
BSU35250	complement(3623938..3624828)	*ftsX*	cell-division ABC transporter	2.051	1.82
BSU16310	1701684..1702682	*fliM*	flagellar motor switching and energizingcomponent	2.050	1.37
BSU37180	complement(3814382..3816499)	*fadF*	putative iron-sulphur-binding reductase	2.049	0.44
BSU05310	complement(576946..578133)	*ydeR*	putative efflux transporter	2.046	1.24
BSU19450	2120767..2121615	*rsbRC*	Component of the piezosome (stressosome)	2.042	1.19
BSU24930	2576367..2576720	*yqzD*	conserved hypothetical protein	2.041	1.92
BSU09430	complement(1020073..1020948)	*citR*	transcriptional regulator CitR (LysR family)	2.038	1.35
BSU29400	3008938..3009864	*ytlI*	transcriptional regulator (LysR family)	2.033	1.66
BSU39500	complement(4059488..4060282)	*yxeM*	putative ABC transporter (binding lipoprotein)	2.032	1.06
BSU25610	complement(2642101..2642844)	*yqeM*	conserved hypothetical protein	2.029	1.54
BSU09480	complement(1026255..1027664)	*yhdI*	putative PLP-dependent transcriptionalregulator	2.027	1.21
BSU31390	complement(3225178..3225570)	*yugI*	putative RNA degradation protein; putativephosphorylase or nucleotidyl transferase	2.027	1.61
BSU35640	complement(3662789..3663097)	*lytA*	membrane bound lipoprotein	2.026	1.10

BSU25270	complement(2607762..2608649)	*glyQ*	glycyl-tRNA synthetase (alpha subunit)	**2.024**	**1.53**
BSU12250	complement(1295748..1296500)	*yjkB*	putative phosphate ABC transporter (ATP-bindingprotein)	**2.020**	**1.01**
BSU35510	3646753..3647406	*yvyE*	putative translation regulator	**2.019**	**1.39**
BSU16070	1678250..1679980	*ylqG*	putative glycosyltransferase	**2.018**	**1.03**
BSU29720	complement(3042566..3043294)	*ytxE*	putative flagellar motor apparatus component	**2.018**	**1.65**
BSU04250	476059..476493	*lrpC*	transcriptional regulator (Lrp/AsnC family)	**2.017**	**1.36**
BSU_tRNA_61	complement(3172872..3172948)	*trnB-Met1*	tRNA-Met	**2.017**	**1.05**
BSU_tRNA_38	952397..952470	*trnD-Trp*	tRNA-Trp	**2.011**	**0.73**
BSU15830	1656064..1656426	*yloU*	conserved hypothetical protein	**2.010**	**1.27**
BSU22310	2340802..2341422	*recU*	Holliday junction resolvase	**2.004**	**1.39**
BSU15070	1575264..1575782	*ylbN*	conserved hypothetical protein	**2.004**	**1.55**
BSU39240	complement(4030710..4031645)	*yxxF*	putative transporter	**2.003**	**1.04**
BSU16460	1715970..1716470	*cheD*	sequence specific deamidase required formethylation of methyl-accepting chemotaxis proteins (MCPs)by CheR	**2.002**	**1.36**
BSU32000	complement(3291511..3292296)	*dhbA*	2,3-dihydro-2,3-dihydroxybenzoate dehydrogenase	**1.984**	**0.14**
BSU33730	complement(3462105..3463250)	*opuBA*	choline ABC transporter (ATP-binding protein)	**1.961**	**0.43**
BSU24720	complement(2557983..2558954)	*comGB*	membrane platform component of the DNA transportmachinery	**1.957**	**0.45**
BSU_misc_RNA_86	4096997..4097409	*srlX*		**1.950**	**0.37**
BSU15600	1633369..1633962	*cysC*	adenylylsulfate kinase	**1.943**	**2.16**
BSU15590	1632208..1633356	*sat*	sulfate adenylyltransferase	**1.867**	**2.13**
BSU14170	1488413..1488868	*ykuP*	short-chain flavodoxin	**1.852**	**0.19**
BSU15610	1634061..1634834	*sumT*	uroporphyrinogen III and precorrin-1C-methyltransferase	**1.832**	**2.02**
BSU01630	complement(182370..183323)	*feuA*	iron hydroxamate-binding lipoprotein	**1.827**	**0.35**
BSU34370	complement(3529151..3529855)	*epsA*	modulator of protein tyrosine kinase EpsB	**1.824**	**0.43**
BSU14150	1487038..1487514	*ykuN*	short-chain flavodoxin	**1.807**	**0.20**
BSU31990	complement(3290289..3291485)	*dhbC*	isochorismate synthase	**1.797**	**0.12**
BSU14160	1487504..1488397	*ykuO*	conserved hypothetical protein	**1.786**	**0.15**
BSU02230	243892..245046	*purT*	phosphoribosylglycinamide formyltransferase 2	**1.777**	**2.15**
BSU13740	1440542..1441273	*queE*	7-carboxy-7-deazaguanine synthase	**1.763**	**2.53**
BSU27190	complement(2778923..2779072)	*yrzI*	conserved hypothetical protein	**1.758**	**0.35**
BSU29690	3040092..3040724	*acuA*	protein acetyltransferase	**1.746**	**2.04**
BSU24630	complement(2553930..2554502)	*sipW*	type I signal peptidase	**1.720**	**0.34**
BSU31980	complement(3288641..3290260)	*dhbE*	2,3-dihydroxybenzoate-AMP ligase	**1.716**	**0.11**
BSU02050	225064..226248	*ybdO*	putative phage protein	**1.687**	**0.46**
BSU31959	complement(3280294..3280503)	*ybdZ*	conserved hypothetical protein	**1.666**	**0.10**
BSU34360	complement(3528462..3529145)	*epsB*	protein tyrosine kinase	**1.662**	**0.41**
BSU31970	complement(3287675..3288613)	*dhbB*	isochorismatase	**1.645**	**0.09**
BSU31960	complement(3280519..3287655)	*dhbF*	siderophore2,3-dihydroxybenzoate-glycine-threonine trimeric esterbacillibactin synthetase	**1.644**	**0.08**
BSU34310	complement(3522170..3523273)	*epsG*	biofilm extracellular matrix formation enzyme	**1.621**	**0.43**
BSU34050	complement(3494985..3495701)	*lutA*	iron-sulfur oxidase component	**1.615**	**2.45**
BSU26670	2725837..2726727	*gltR*	transcriptional regulator (LysR family)	**1.613**	**2.11**
BSU01600	complement(179595..180347)	*ybbA*	putative iron-chelator esterase	**1.607**	**0.33**
BSU13720	1439448..1440107	*queC*	pre-queuosine 0 synthase	**1.580**	**2.12**
BSU26630	2721778..2722644	*yrdQ*	putative transcriptional regulator (LysRfamily)	**1.546**	**2.64**
BSU07480	820867..822330	*yfmG*	conserved hypothetical protein	**1.536**	**0.26**
BSU33140	complement(3399092..3400360)	*yvqJ*	putative efflux protein	**1.535**	**0.44**
BSU01620	complement(181347..182351)	*feuB*	iron-uptake protein	**1.516**	**0.30**
BSU34290	complement(3520030..3521106)	*epsI*	putative polysaccharide pyruvyl transferase	**1.503**	**0.31**
BSU34350	complement(3526407..3528203)	*epsC*	putative UDP-sugar epimerase	**1.501**	**0.44**
BSU01640	complement(183414..185003)	*btr*	transcriptional activator (AraC/XylS family) ofsynthesis and uptake of the siderophore bacillibactin	**1.497**	**0.50**
BSU01610	complement(180344..181354)	*feuC*	iron-uptake protein	**1.489**	**0.31**
BSU04536	complement(506455..506619)	*fbpA*	regulator of iron homeostasis	**1.483**	**0.34**
BSU34265	complement(3517485..3519002)	*epsK*	putative extracellular matrix componentexporter	**1.433**	**0.32**
BSU33770	3465776..3466387	*spbC*	killing factor SdpC	**1.428**	**0.23**
BSU39370	4045245..4046510	*hutI*	imidazolone-5-propionate hydrolase	**1.417**	**3.98**
BSU34280	complement(3518999..3520033)	*epsJ*	putative glycosyl transferase	**1.409**	**0.34**
BSU34300	complement(3521111..3522145)	*epsH*	putative glycosyltransferase involved in biofilmformation	**1.394**	**0.37**
BSU37220	complement(3819220..3819744)	*ywjB*	putative oxidoreductase	**1.390**	**0.45**
BSU15560	1629320..1629970	*pyrE*	orotate phosphoribosyltransferase	**1.375**	**0.45**
BSU34240	complement(3516233..3516883)	*epsM*	putative O-acetyltransferase	**1.284**	**0.29**
BSU04530	complement(506322..506501)	*fbpB*	regulator of iron homeostasis	**1.256**	**0.35**
BSU34320	complement(3523270..3524424)	*epsF*	putative glycosyltransferase involved inextracellular matrix formation	**1.249**	**0.36**
BSU34230	complement(3515062..3516228)	*epsN*	putative aminotransferase	**1.248**	**0.26**
BSU34250	complement(3516880..3517488)	*epsL*	putative phosphotransferase involved inextracellular matrix synthesis	**1.247**	**0.32**
BSU32870	3374492..3374959	*yusO*	putative transcriptional regulator (MarRfamily)	**1.231**	**2.07**
BSU34220	complement(3514115..3515083)	*epsO*	putative pyruvyl transferase	**1.223**	**0.26**
BSU24620	complement(2553081..2553866)	*tasA*	major biofilm matrix component	**1.221**	**0.33**
BSU09420	1018998..1020002	*lytE*	cell wall hydrolase; phosphatase-associatedprotein (major autolysin)	**1.218**	**2.02**
BSU07735	844253..844645	*yflB*	conserved hypothetical protein	**1.211**	**0.45**
BSU33710	complement(3460503..3461423)	*opuBC*	choline ABC transporter (choline-bindinglipoprotein)	**1.207**	**0.45**
BSU08080	881049..882245	*acoC*	acetoin dehydrogenase E2 component(dihydrolipoamide acetyltransferase)	**1.198**	**0.48**
BSU34330	complement(3524417..3525253)	*epsE*	bifunctional flagellar clutch andglycosyltransferase	**1.184**	**0.38**
BSU02040	complement(224075..224932)	*ybdN*	putative phage protein	**1.179**	**0.25**
BSU28910	complement(2955209..2955649)	*lrgA*	antiholin factor	**1.174**	**0.28**
BSU36310	complement(3740206..3740547)	*ssbB*	single-strand DNA-binding protein	**1.172**	**0.47**
BSU39950	complement(4103762..4104187)	*yxaJ*	putative integral membrane protein	**1.164**	**0.50**
BSU33720	complement(3461435..3462088)	*opuBB*	choline ABC transporter (permease)	**1.162**	**0.47**
BSU06830	750959..752089	*rapH*	response regulator aspartate phosphatase	**1.154**	**0.42**
BSU38770	3979753..3981105	*cimH*	citrate/malate/H+ symporter	**1.152**	**2.01**
BSU10270	1100980..1102521	*lcfB*	long-chain fatty-acid-CoA ligase (degradative)	**1.138**	**0.49**
BSU08090	882266..883642	*acoL*	acetoin dehydrogenase E3 component(dihydrolipoamide dehydrogenase)	**1.118**	**0.48**
BSU39360	4043574..4045232	*hutU*	urocanase	**1.109**	**4.54**
BSU39390	4047538..4048965	*hutM*	histidine permease	**1.098**	**4.04**
BSU13700	complement(1435628..1437727)	*clpE*	ATP-dependent Clp protease (class III stressgene)	**1.057**	**4.91**
BSU34580	complement(3551479..3552363)	*mdxJ*	putative component of maltodextrin transporter	**1.048**	**2.24**
BSU34600	complement(3553206..3554513)	*mdxF*	maltodextrin ABC transport system (permease)	**0.987**	**2.40**
BSU39380	4046503..4047462	*hutG*	formiminoglutamate hydrolase	**0.980**	**4.30**
BSU34610	complement(3554553..3555806)	*mdxE*	maltose/maltodextrin-binding lipoprotein	**0.978**	**2.35**
BSU05870	631808..632755	*gmuF*	phosphohexomutase; cupin family	**0.968**	**2.08**
BSU37230	complement(3819754..3821481)	*ywjA*	putative ABC lipid transporter (ATP-bindingprotein)	**0.957**	**0.40**
BSU34590	complement(3552369..3553205)	*mdxG*	maltodextrin ABC transporter (permease)	**0.954**	**2.07**
BSU34570	complement(3549228..3551501)	*mdxK*	maltose phosphorylase	**0.940**	**2.14**
BSU24580	2549775..2551448	*yqhH*	putative RNA polymerase-associated helicaseprotein	**0.938**	**0.45**
BSU25760	2652993..2653463	*spoIVCB*	RNA polymerase sporulation-specific sigma-Kfactor precursor (Sigma-27) (N-terminal half)	**0.907**	**0.49**
BSU06839	752079..752252	*phrH*	hexapeptide inhibitor of regulatory cascade	**0.890**	**0.39**
BSU15540	1627718..1628653	*pyrD*	dihydroorotate dehydrogenase (catalyticsubunit)	**0.889**	**0.48**
BSU17170	1792012..1792761	*pksI*	decarboxylase involved in polyketide synthesis	**0.858**	**0.45**
BSU11290	1205981..1206538	*yjaV*	putative NAD(P) binding enzyme	**0.833**	**0.46**
BSU11080	1185001..1185588	*yitQ*	hypothetical protein	**0.828**	**0.49**
BSU32830	complement(3368839..3370014)	*fadA*	acetyl-CoA C-acyltransferase	**0.823**	**0.26**
BSU39560	complement(4064536..4065093)	*yxeG*	putative integral inner membrane protein	**0.818**	**0.44**
BSU39870	complement(4095356..4095835)	*yxbD*	putative acetyltransferase	**0.814**	**0.40**
BSU24650	2555519..2555845	*yqzG*	putative exported protein	**0.805**	**0.49**
BSU33550	complement(3446237..3448132)	*yvaC*	putative integral inner membrane protein	**0.802**	**0.46**
BSU32820	complement(3367040..3368824)	*fadE*	acyl-CoA dehydrogenase (FAD dependent)	**0.789**	**0.26**
BSU11790	1252177..1252533	*yjcA*	sporulation-specific protein	**0.778**	**0.47**
BSU01960	217697..219040	*skfF*	sporulation killing factor biosynthesis andexport; ABC transporter (permease)	**0.777**	**0.33**
BSU28520	complement(2915365..2916342)	*etfA*	electron transfer flavoprotein (alpha subunit)	**0.773**	**0.46**
BSU24010	2494656..2495825	*bmr*	multidrug-efflux transporter	**0.752**	**0.48**
BSU17240	complement(1860014..1860370)	*ymzB*	conserved hypothetical protein	**0.748**	**0.43**
BSU28530	complement(2916378..2917151)	*etfB*	electron transfer flavoprotein (beta subunit)	**0.735**	**0.50**
BSU19020	2074343..2075779	*yobN*	putative amine oxidase	**0.728**	**0.44**
BSU32840	complement(3370025..3372394)	*fadN*	enoyl-CoA hydratase / 3-hydroxyacyl-CoAdehydrogenase	**0.726**	**0.24**
BSU36680	complement(3770365..3770844)	*ywmF*	putative integral membrane protein	**0.709**	**0.47**
BSU31820	complement(3264265..3264501)	*yuzF*	conserved hypothetical protein	**0.702**	**0.44**
BSU39880	complement(4095915..4096907)	*yxbC*	conserved hypothetical protein	**0.697**	**0.43**
BSU01980	219607..220032	*skfH*	sibling killing effect; sporulation killingfactor biosynthesis and export	**0.693**	**0.35**
BSU37510	3849818..3851893	*pbpG*	sporulation specific penicillin-binding protein	**0.679**	**0.41**
BSU10730	complement(1150850..1151020)	*yisI*	Spo0A-P phosphatase	**0.676**	**0.49**
BSU33050	3390782..3392230	*gerAA*	component of the GerA germination receptor	**0.653**	**0.50**
BSU30739	3141900..3142058	*ytzL*	conserved hypothetical protein	**0.651**	**0.42**
BSU37390	3837840..3838559	*albC*	putative transporter involved in subtilosinproduction	**0.650**	**0.48**
BSU21546	2273594..2273710	*bsrG*	phage toxin; type I toxin-antitoxin system	**0.642**	**0.42**
BSU30620	3134144..3134956	*ytlD*	putative permease of ABC transporter	**0.640**	**0.49**
BSU38930	3996829..3997092	*yxjJ*	hypothetical protein	**0.639**	**0.44**
BSU01950	216913..217632	*skfE*	sporulation killing factor biosynthesis andexport; ABC transporter (binding protein)	**0.638**	**0.27**
BSU01970	219087..219602	*skfG*	sporulation killing factor biosynthesis andexport	**0.636**	**0.36**
BSU13170	complement(1382677..1383147)	*guaD*	guanine deaminase	**0.623**	**0.50**

BSU07750	844770..846185	*yflA*	putative aminoacid transporter	**0.618**	**0.37**
BSU17640	complement(1897941..1899125)	*alrB*	alanine racemase	**0.613**	**0.41**
BSU23480	complement(2445094..2446263)	*dacF*	D-alanyl-D-alanine carboxypeptidase (penicilinbinding protein)	**0.605**	**0.37**
BSU26890	complement(2747984..2748817)	*csn*	chitosanase	**0.603**	**0.37**
BSU35060	complement(3602588..3603805)	*cypX*	cyclo-L-leucyl-L-leucyl dipeptide oxidase	**0.603**	**0.47**
BSU10280	complement(1102560..1102955)	*yhfM*	hypothetical protein	**0.603**	**0.49**
BSU19310	2100580..2102067	*dhaS*	putative aldehyde dehydrogenase	**0.596**	**0.41**
BSU40030	complement(4110949..4111980)	*yxaB*	putative exopolysaccharide pyruvyl transferase	**0.596**	**0.46**
BSU35070	complement(3603821..3604567)	*yvmC*	cyclodipeptide synthase	**0.594**	**0.49**
BSU27450	2803990..2804640	*glnM*	glutamine ABC transporter (permease)	**0.594**	**0.47**
BSU09400	1015647..1017053	*spoVR*	involved in spore cortex synthesis (stage Vsporulation)	**0.593**	**0.26**
BSU24490	2541051..2542007	*yqhQ*	conserved hypothetical protein	**0.588**	**0.46**
BSU01935	215404..216894	*skfC*	sporulation killing factor biosynthesis andexport	**0.586**	**0.21**
BSU04380	complement(492989..493441)	*ydaT*	conserved hypothetical protein	**0.585**	**0.41**
BSU24370	complement(2533010..2533699)	*spoIIIAG*	stage III sporulation engulfment assemblyprotein	**0.572**	**0.48**
BSU37370	3836323..3837669	*albA*	putative antilisterial bacteriocin (subtilosin)production enzyme	**0.570**	**0.40**
BSU40260	complement(4137362..4137610)	*yycQ*	putative conserved membrane protein	**0.570**	**0.49**
BSU01910	213941..214108	*skfA*	sporulation killing factor A	**0.566**	**0.17**
BSU31470	complement(3231812..3232429)	*kapD*	putative exoribonuclease (3'-5')	**0.560**	**0.48**
BSU01570	complement(159779..160543)	*ybaN*	polysaccharide deacetylase involved insporulation	**0.558**	**0.48**
BSU37380	3837682..3837843	*albB*	putative membrane component involved insubtilosin production	**0.558**	**0.43**
BSU14670	1537441..1538238	*suhB*	inositol monophosphatase	**0.557**	**0.49**
BSU03400	369773..370105	*yckD*	putative exported protein	**0.556**	**0.41**
BSU06590	complement(716780..718357)	*yerD*	putative flavoenzyme	**0.554**	**0.39**
BSU32350	complement(3322463..3323227)	*yunB*	putative protein involved in spore formation	**0.551**	**0.39**
BSU39930	4101166..4102365	*yxaM*	putative efflux transporter	**0.546**	**0.28**
BSU36990	complement(3795870..3796157)	*ywkF*	hypothetical protein	**0.543**	**0.50**
BSU19970	complement(2156757..2157110)	*yosX*	conserved hypothetical protein; phage SPbeta	**0.539**	**0.50**
BSU01920	214175..215407	*skfB*	synthesis of sporulation killing factor A	**0.538**	**0.16**
BSU12500	complement(1320570..1321166)	*xkdA*	PBSX phage protein, putative peptidase	**0.536**	**0.34**
BSU37360	3836146..3836298	*sboX*	putative bacteriocin-like product	**0.535**	**0.36**
BSU39920	4098926..4101169	*asnH*	asparagine synthetase (glutamine-hydrolyzing)	**0.524**	**0.28**
BSU32100	complement(3300034..3301254)	*yumB*	putative NAD-disulfide oxidoreductase	**0.523**	**0.34**
BSU39910	4098423..4098905	*yxnB*	hypothetical protein	**0.522**	**0.22**
BSU39900	4098150..4098419	*yxbA*	conserved hypothetical protein	**0.522**	**0.25**
BSU04740	523650..524249	*rsbX*	serine phosphatase	**0.520**	**0.47**
BSU22930	complement(2399152..2400069)	*sleB*	spore cortex-lytic enzyme	**0.520**	**0.38**
BSU21310	complement(2246151..2246480)	*yozP*	hypothetical protein; phage SPbeta	**0.519**	**0.45**
BSU17420	1874203..1875171	*spoVK*	mother cell sporulation ATPase	**0.518**	**0.40**
BSU07760	complement(846182..847258)	*yfkT*	putative spore germination integral innermembrane protein	**0.517**	**0.38**
BSU39890	4097416..4098150	*yxbB*	putative S-adenosylmethionine-dependentmethyltransferase	**0.517**	**0.21**
BSU13360	1400188..1400739	*ykoP*	conserved hypothetical protein	**0.517**	**0.40**
BSU00650	73106..73843	*yabS*	conserved hypothetical protein	**0.516**	**0.39**
BSU11730	1248665..1249348	*cotO*	spore outer coat protein	**0.511**	**0.42**
BSU11260	1204506..1204685	*yjzC*	conserved hypothetical protein	**0.507**	**0.39**
BSU23440	complement(2442684..2443304)	*spoVAA*	stage V sporulation protein AA	**0.506**	**0.41**
BSU22920	complement(2397765..2399117)	*ypeB*	spore membrane component	**0.506**	**0.34**
BSU19270	2098859..2099122	*yozN*	conserved hypothetical protein	**0.504**	**0.37**
BSU18510	2019421..2019726	*yoxC*	conserved hypothetical protein	**0.504**	**0.48**
BSU05570	complement(602441..602713)	*ydgB*	conserved hypothetical protein	**0.500**	**0.38**
BSU10410	complement(1116583..1116816)	*yhzC*	hypothetical protein	**0.499**	**0.38**
BSU37350	3836058..3836189	*sboA*	subtilosin A	**0.499**	**0.34**
BSU09680	complement(1042885..1044246)	*nhaC*	Na+/H+ antiporter	**0.498**	**0.57**
BSU06190	671994..673019	*ydjG*	putative phage replication protein	**0.498**	**0.55**
BSU30650	complement(3136238..3136675)	*dps*	DNA-protecting protein, ferritin	**0.497**	**0.61**
BSU30640	complement(3135668..3136072)	*ytkC*	putative autolytic amidase	**0.497**	**0.26**
BSU30595	3132370..3133374	*ytlA*	putative ABC transporter component	**0.497**	**0.43**
BSU29249	2995699..2995890	*ytzJ*	conserved hypothetical protein	**0.496**	**0.64**
BSU24150	complement(2510806..2511945)	*mmgC*	short chain acyl-CoA dehydrogenase	**0.493**	**0.44**
BSU31279	complement(3213342..3213596)	*yuzH*	conserved hypothetical protein	**0.493**	**0.61**
BSU05520	complement(599107..599343)	*ydzH*	conserved hypothetical protein	**0.493**	**0.44**
BSU29520	complement(3019533..3020027)	*yteJ*	putative integral inner membrane protein	**0.493**	**1.02**
BSU06049	654333..654692	*ydzV*	conserved hypothetical protein; phage terminase(fragment)	**0.492**	**0.81**
BSU05099	556562..556738	*ydzM*	putative phage protein	**0.491**	**0.24**
BSU31322	3218854..3219840	*yugO*	putative potassium channel protein	**0.488**	**0.56**
BSU14800	complement(1548681..1549310)	*ylaJ*	putative lipoprotein	**0.487**	**0.39**
BSU00840	101927..102484	*mcsA*	activator of protein kinase McsB	**0.486**	**0.77**
BSU20060	complement(2162108..2165614)	*nrdEB*	SPbeta phage ribonucleoside reductase alphasubunit	**0.486**	**0.89**
BSU00080	complement(14847..15794)	*yaaC*	conserved hypothetical protein	**0.485**	**0.46**
BSU04600	513283..514764	*ydbT*	conserved hypothetical protein	**0.485**	**0.77**
BSU25390	complement(2618466..2619779)	*yqeZ*	putative membrane bound hydrolase	**0.485**	**0.84**
BSU33250	3411684..3412094	*yvrL*	putative integral inner membrane protein	**0.485**	**0.49**
BSU21670	2286448..2287053	*ypqP*	putative capsular polysaccharide biosynthesisenzyme fragment; C-terminal part of YpqP	**0.484**	**0.41**
BSU35240	complement(3622356..3623798)	*ctpB*	PDZ-containing carboxyl-terminal proteaseprocessing protease	**0.484**	**0.40**
BSU35120	complement(3607325..3608422)	*yvlB*	conserved hypothetical protein	**0.484**	**0.77**
BSU19530	2126549..2126938	*yodA*	putative tautomerase	**0.484**	**2.43**
BSU11120	1188689..1189531	*yitT*	putative integral inner membrane protein	**0.483**	**0.55**
BSU40250	complement(4135798..4137024)	*yycR*	putative dehydrogenase	**0.483**	**0.56**
BSU00520	58783..59397	*ctc*	ribosomal protein Ctc, binding 5S RNA	**0.482**	**0.56**
BSU05550	complement(601741..602172)	*cotP*	spore coat protein	**0.482**	**0.35**
BSU20810	complement(2206682..2207758)	*yopP*	putative phage integrase; phage SPbeta	**0.481**	**0.65**
BSU04710	522088..522417	*rsbV*	anti-anti-sigma factor (antagonist of RsbW)	**0.480**	**0.54**
BSU33850	complement(3471841..3472476)	*yvbG*	putative integral inner membrane protein	**0.479**	**0.46**
BSU05500	598154..598543	*ydfP*	putative membrane bound oxidoreductase	**0.479**	**0.49**
BSU30000	complement(3070246..3071403)	*ythQ*	putative ABC transporter (permease)	**0.479**	**0.84**
BSU25380	complement(2617449..2618444)	*yqfA*	conserved hypothetical protein	**0.478**	**1.03**
BSU24750	2561585..2562913	*yqhB*	putative membrane associated protein	**0.478**	**0.47**
BSU19430	complement(2118504..2119127)	*yojJ*	putative enzyme with DAC domain	**0.478**	**0.56**
BSU18590	complement(2028175..2028579)	*yoaG*	putative permease	**0.477**	**0.86**
BSU20830	complement(2208008..2208325)	*yopN*	hypothetical protein; phage SPbeta	**0.477**	**0.85**
BSU40450	4159005..4159205	*yycD*	conserved hypothetical protein	**0.477**	**0.35**
BSU35110	complement(3607123..3607320)	*yvlC*	putative regulator (stress mediated)	**0.476**	**0.74**
BSU15520	1623736..1626951	*pyrAB*	pyrimidine-specific carbamoyl-phosphatesynthetase (large subunit)	**0.476**	**0.52**
BSU21160	2237321..2237791	*yonA*	conserved hypothetical protein; phage SPbeta	**0.476**	**1.04**
BSU36420	complement(3748421..3748702)	*spoIIID*	transcriptional regulator	**0.475**	**0.30**
BSU01670	complement(188408..189733)	*amiE*	amidase hydrolyzing N-acetylmuramyl-L-Ala bondof MurNAc peptides	**0.475**	**0.71**
BSU04730	522862..523650	*sigB*	RNA polymerase sigma-37 factor (sigma(B))	**0.474**	**0.48**
BSU36670	complement(3770104..3770292)	*csbD*	stress response protein	**0.474**	**0.40**
BSU07250	792682..795867	*cypD*	putative bifunctional P-450/NADPH-P450 reductase1	**0.474**	**0.41**
BSU11490	1226938..1227516	*yjbC*	putative thiol oxidation management factor;putative acetyltransferase	**0.474**	**0.54**
BSU09959	1071613..1071699	*sscB*	spore and germination protein	**0.473**	**0.39**
BSU14600	1530537..1531865	*pdhC*	pyruvate dehydrogenase (dihydrolipoamideacetyltransferase E2 subunit)	**0.472**	**1.18**
BSU12069	complement(1278205..1278399)	*yjzH*	conserved hypothetical protein	**0.471**	**0.62**
BSU36120	3721415..3722008	*ywrB*	putative anion transporter	**0.471**	**0.78**
BSU14610	1531870..1533282	*pdhD*	dihydrolipoamide dehydrogenase E3 subunit ofboth pyruvate dehydrogenase and 2-oxoglutaratedehydrogenase complexes	**0.470**	**1.12**
BSU30850	complement(3154735..3155553)	*ytdA*	putative UTP-glucose-1-phosphateuridylyltransferase	**0.469**	**0.46**
BSU35170	complement(3612945..3614930)	*uvrB*	excinuclease ABC (subunit B)	**0.469**	**0.42**
BSU25370	complement(2617005..2617424)	*yqfB*	conserved hypothetical protein	**0.469**	**1.06**
BSU24770	complement(2564026..2564406)	*mgsR*	transcriptional regulator of stress	**0.468**	**0.64**
BSU04720	522414..522896	*rsbW*	switch protein/serine kinase and anti-sigmafactor (inhibitory sigma-B binding protein)	**0.467**	**0.53**
BSU32570	complement(3347051..3347905)	*frlD*	fructoselysine kinase	**0.467**	**0.76**
BSU38630	3964997..3966640	*katX*	major catalase in spores	**0.466**	**0.37**
BSU08600	930818..931807	*csbB*	putative glycosyl transferase	**0.466**	**0.60**
BSU27850	complement(2845955..2847061)	*nadA*	quinolinate synthetase	**0.465**	**0.49**
BSU20580	2195986..2196264	*yoqM*	putative membrane bound protein; phage SPbeta	**0.464**	**0.44**
BSU38790	3982973..3983173	*yxzE*	putative bacteriocin	**0.464**	**0.54**
BSU03050	329774..330739	*ldh*	L-lactate dehydrogenase	**0.461**	**0.91**
BSU37240	complement(3821570..3823072)	*ywiE*	cardiolipin synthetase	**0.461**	**0.42**
BSU19320	2102168..2104066	*sqhC*	squalene-hopene cyclase	**0.460**	**0.42**
BSU04590	512814..513293	*ydbS*	conserved hypothetical protein	**0.460**	**0.75**
BSU23900	2484911..2485639	*yqjF*	conserved hypothetical protein	**0.459**	**0.50**
BSU35130	complement(3608447..3608773)	*yvlA*	conserved hypothetical protein	**0.458**	**0.72**
BSU04180	complement(470957..471502)	*ydaC*	putative methyltransferase	**0.457**	**0.45**
BSU30700	complement(3138978..3139226)	*rpmEB*	ribosomal protein L31	**0.457**	**0.49**
BSU26850	2742909..2743889	*yrpG*	putative oxidoreductase	**0.456**	**0.69**
BSU11990	complement(1269733..1270080)	*yjdB*	putative exported protein	**0.456**	**0.15**
BSU04160	467130..469214	*mtlR*	transcriptional regulator	**0.456**	**0.60**

BSU38430	complement(3944560..3945420)	*gspA*	putative glycosyl transferase (general stressprotein)	0.454	0.51
BSU09530	1030265..1031260	*yhdN*	aldo/keto reductase specific for NADPH	0.454	0.48
BSU19730	complement(2143660..2144349)	*yodS*	putative aminoacyloate CoA-transferase	0.453	0.37
BSU36060	complement(3716009..3717097)	*cotH*	spore coat protein (inner)	0.453	0.39
BSU21950	2308495..2308764	*ypzA*	conserved hypothetical protein	0.449	0.51
BSU01660	complement(186452..188380)	*nagZ*	N-acetylglucosaminidase lipoprotein	0.449	0.68
BSU21229	2241154..2241765	*youA*	hypothetical protein; phage SPbeta	0.449	0.95
BSU07930	866331..867125	*yfkD*	conserved hypothetical protein	0.448	0.46
BSU33780	complement(3466434..3467057)	*sdpI*	integral membrane regulator of autophagy	0.446	0.44
BSU32360	complement(3323300..3323605)	*yunC*	putative RNA binding protein	0.445	0.36
BSU19260	complement(2098316..2098792)	*yocM*	putative spore coat protein	0.445	0.62
BSU06910	756417..756986	*cotJC*	component of the inner spore coat	0.444	0.43
BSU38060	3905333..3906103	*ywcJ*	formate/nitrite transporter	0.443	0.86
BSU14580	1528326..1529441	*pdhA*	pyruvate dehydrogenase (E1 alpha subunit)	0.442	1.33
BSU19150	complement(2086743..2087525)	*yocB*	conserved hypothetical protein	0.441	0.42
BSU33340	complement(3421465..3421605)	*sspJ*	small acid-soluble spore protein	0.440	0.42
BSU36640	complement(3766714..3768423)	*ureC*	urease (alpha subunit)	0.439	0.79
BSU19250	complement(2097692..2098024)	*yocL*	hypothetical protein	0.439	0.57
BSU00250	35845..36459	*xpaC*	putative phosphatase	0.439	0.80
BSU16720	1743924..1744181	*ymxH*	conserved hypothetical protein	0.437	0.56
BSU10710	complement(1149958..1150191)	*gerPB*	spore germination protein	0.437	0.52
BSU19110	2084214..2084759	*yobW*	mother cell-specific membrane sporulationprotein	0.436	0.28
BSU05100	556763..557449	*yddT*	putative phage protein	0.435	0.38
BSU36660	complement(3768791..3769108)	*ureA*	urease (gamma subunit)	0.433	1.00
BSU30010	complement(3071400..3072110)	*ythP*	putative ABC transporter (ATP-binding protein)	0.432	0.74
BSU23300	complement(2431737..2432081)	*ypuD*	hypothetical protein	0.429	0.62
BSU20970	complement(2217133..2217639)	*yonX*	conserved hypothetical protein; phage SPbeta	0.429	0.58
BSU40529	complement(4166815..4166964)	*yyzH*	hypothetical protein	0.429	0.40
BSU37420	3841017..3842297	*albF*	putative peptidase involved in subtilosinproduction	0.426	0.47
BSU36550	complement(3759702..3760553)	*spoIIQ*	forespore protein required for alternativeengulfment	0.425	0.69
BSU28320	2897788..2898123	*ysnD*	inner spore coat protein	0.424	0.30
BSU02830	305658..306434	*ycdF*	putative glucose 1-dehydrogenase	0.424	0.49
BSU12410	complement(1313840..1314304)	*yjoA*	conserved hypothetical protein	0.424	0.79
BSU40000	4108058..4109128	*yxnA*	putative oxidoreductase	0.424	0.43
BSU17980	complement(1926128..1926274)	*sspP*	small acid-soluble spore protein	0.423	0.29
BSU00260	36478..37638	*yaaN*	conserved hypothetical protein	0.422	0.70
BSU00830	101449..101913	*ctsR*	transcriptional regulator	0.422	0.76
BSU19290	complement(2099446..2099790)	*yozO*	conserved hypothetical protein	0.421	0.59
BSU22800	complement(2386195..2387673)	*spoIVA*	morphogenetic stage IV sporulation protein	0.421	0.30
BSU21390	2260333..2262267	*yomE*	putative glycosyl hydrolase; phage SPbeta	0.421	1.15
BSU14980	1568065..1568304	*ylbE*	conserved hypothetical protein	0.421	0.33
BSU08570	928803..929321	*yfhK*	conserved hypothetical protein	0.420	0.37
BSU33790	complement(3467054..3467326)	*sdpR*	transcriptional regulator (ArsR family)	0.420	0.38
BSU33410	3427802..3428287	*yvgO*	conserved hypothetical protein	0.418	0.17
BSU35830	complement(3692533..3693906)	*ywtG*	putative carbohydrate transporter	0.417	0.48
BSU18600	2029020..2029313	*yozQ*	conserved hypothetical protein; conserved inBacilli	0.416	0.58
BSU06490	705441..706871	*purF*	glutamine phosphoribosylpyrophosphateamidotransferase	0.415	0.48
BSU06220	complement(674832..675857)	*ydjJ*	putative membrane associated potassium channel	0.415	0.52
BSU01650	complement(185194..186438)	*ybbC*	conserved hypothetical protein	0.413	0.67
BSU32469	3335414..3335545	*yuzJ*	putative type I toxin	0.413	0.31
BSU10950	1173333..1174091	*slpS*	phosphosulfolactate synthase	0.411	0.30
BSU14660	1537113..1537301	*ykzI*	conserved hypothetical protein	0.411	0.48
BSU07880	862004..862474	*yfkJ*	protein-tyrosine-phosphatase	0.411	0.47
BSU10080	complement(1081413..1082804)	*yhfA*	putative transporter	0.411	0.50
BSU18620	2031439..2032890	*yoaI*	putative 4-hydroxyphenylacetate-3-hydroxylase	0.410	0.27
BSU09450	1022231..1023100	*yhdF*	putative NAD(P)-dependent dehydrogenase	0.410	0.46
BSU07890	862492..862812	*yfkI*	conserved hypothetical protein	0.410	0.52
BSU06660	complement(726840..728318)	*opuE*	proline transporter	0.409	0.37
BSU17900	1922017..1922463	*pcfA*	factor controlling DNA replication	0.408	0.39
BSU28810	complement(2949053..2950024)	*abnA*	arabinan-endo 1,5-alpha-L-arabinase	0.406	0.65
BSU02590	complement(281769..282155)	*ycbP*	putative inner integral membrane protein	0.405	0.37
BSU05790	624492..625109	*ydhK*	hypothetical protein	0.404	0.55
BSU11410	1217326..1218078	*yjbA*	putative nucleic acid binding protein	0.401	0.62
BSU36010	complement(3709628..3711340)	*alsS*	alpha-acetolactate synthase	0.400	0.37
BSU28830	2951490..2951882	*ysdB*	conserved hypothetical protein	0.398	0.62
BSU36490	complement(3755291..3755860)	*ywoC*	putative hydrolase	0.398	0.73
BSU28380	complement(2902002..2903102)	*gerM*	germination (cortex hydrolysis) and sporulation(stage II, multiple polar septa) lytic enzyme	0.396	0.69
BSU39780	4085608..4086540	*iolS*	aldo-keto reductase	0.396	1.27
BSU24130	complement(2508221..2509639)	*mmgE*	2-methylcitrate dehydratase	0.396	0.34
BSU37620	3861437..3862213	*rsfA*	prespore-specific regulatory gene	0.395	0.42
BSU11839	1256109..1256363	*yjzE*	hypothetical protein	0.395	0.53
BSU36720	complement(3774400..3774561)	*ywmE*	hypothetical protein	0.394	0.44
BSU35180	complement(3615116..3615346)	*csbA*	putative membrane protein	0.394	0.48
BSU00660	73809..74825	*yabT*	putative serine/threonine-protein kinase	0.392	0.37
BSU36650	complement(3768420..3768794)	*ureB*	urease (beta subunit)	0.392	0.79
BSU02110	complement(230819..231079)	*ybyB*	conserved hypothetical protein	0.391	0.45
BSU36960	complement(3794166..3794609)	*ywlB*	conserved hypothetical protein	0.391	0.38
BSU04340	488830..490554	*ydaP*	putative enzyme with pyruvate as substrate	0.388	0.32
BSU13470	complement(1413800..1413994)	*sspD*	small acid-soluble spore protein(alpha/beta-type SASP)	0.387	0.34
BSU19689	complement(2139179..2139457)	*yokU*	conserved hypothetical protein	0.386	0.23
BSU24000	2493662..2494555	*bmrU*	putative lipid kinase BmrU	0.384	0.40
BSU40420	complement(4155433..4156725)	*purA*	adenylosuccinate synthetase	0.383	0.88
BSU02100	229525..230778	*cypC*	fatty acid beta-hydroxylating cytochrome P450	0.383	0.40
BSU39590	4066210..4066563	*yxeD*	conserved hypothetical protein	0.382	0.28
BSU33960	complement(3484072..3485466)	*araE*	arabinose-related compounds permease	0.382	0.54
BSU19240	complement(2097126..2097617)	*yocK*	putative general stress protein	0.382	0.48
BSU15300	1599283..1603584	*bpr*	bacillopeptidase F	0.381	0.28
BSU36510	3756790..3758004	*amtB*	ammonium transporter	0.380	0.72
BSU19720	complement(2143022..2143675)	*yodR*	putative acyloate-acetoacetate CoA-transferase	0.379	0.35
BSU15330	1605630..1606412	*sigG*	RNA polymerase sporulation-specific sigma factor(sigma-G)	0.379	0.18
BSU14590	1529445..1530422	*pdhB*	pyruvate dehydrogenase (E1 beta subunit)	0.378	1.18
BSU10930	complement(1171755..1172465)	*yitB*	putative phospho-adenylylsulfatesulfotransferase	0.376	0.23
BSU36000	complement(3708799..3709566)	*alsD*	alpha-acetolactate decarboxylase	0.375	0.43
BSU07920	865205..866260	*yfkE*	putative H+/Ca2+ antiporter	0.375	0.41
BSU11320	complement(1207818..1208057)	*yjzB*	conserved hypothetical protein	0.374	0.32
BSU21220	2240339..2241136	*yomU*	hypothetical protein; phage SPbeta	0.373	0.86
BSU24740	complement(2560489..2561442)	*yqxL*	putative CorA-type Mg(2+) transporter	0.372	0.46
BSU26970	2753452..2754588	*adhB*	putative oxidoreductase	0.372	0.33
BSU00170	complement(25221..25766)	*yaaI*	putative isochorismatase	0.372	0.38
BSU25600	2641214..2642035	*comER*	putative pyrroline-5'-carboxylate reductase	0.371	0.23
BSU07850	859745..860263	*yfkM*	general stress protein 18	0.371	0.41
BSU23470	complement(2444645..2444998)	*spoIIAA*	anti-anti-sigma factor (antagonist of SpoIIAB)	0.369	0.41
BSU38760	complement(3977791..3979197)	*cydA*	cytochrome bb' ubiquinol oxidase (subunit I)	0.369	1.00
BSU08580	929406..929738	*yfhL*	SdpC immunity factor	0.368	0.51
BSU06420	698612..699100	*purE*	N5-carboxyaminoimidazole ribonucleotide mutase	0.367	0.89
BSU08670	938243..938587	*ygaB*	conserved hypothetical protein	0.367	0.38
BSU21250	2242414..2243631	*yomR*	conserved hypothetical protein; phage SPbeta	0.367	0.84
BSU22240	2337577..2337996	*ypqA*	hypothetical protein	0.365	0.22
BSU39840	4091845..4092666	*yxbG*	putative oxidoreductase	0.364	0.54
BSU21210	2239586..2240296	*yomV*	conserved hypothetical protein; phage SPbeta	0.364	1.01
BSU21140	2235694..2236230	*yonC*	conserved hypothetical protein; phage SPbeta	0.363	1.14
BSU39040	complement(4007803..4008093)	*yxiS*	hypothetical protein	0.363	0.42
BSU20390	complement(2184450..2185421)	*yorG*	hypothetical protein; phage SPbeta	0.363	0.67
BSU37430	3842294..3842995	*albG*	putative integral inner membrane proteininvolved in subtilosin production and immunity	0.362	0.44
BSU12160	1285591..1288548	*yjgC*	putative formate dehydrogenase	0.361	0.30
BSU08760	complement(953373..954149)	*spo0M*	sporulation-control gene	0.360	1.15
BSU23460	complement(2444208..2444648)	*spoIIAB*	anti-sigma factor (antagonist of sigma(F)) andserine kinase	0.359	0.44
BSU24360	complement(2532353..2533009)	*spoIIIAH*	stage III sporulation ratchet engulfmentprotein	0.359	0.44
BSU23450	complement(2443429..2444196)	*sigF*	RNA polymerase sporulation-specific sigma factor(sigma-F)	0.359	0.41
BSU10700	complement(1149318..1149935)	*gerPC*	spore germination protein	0.355	0.25
BSU08510	complement(924633..925544)	*yfhF*	putative nucleotide binding protein	0.354	0.50
BSU31321	3218525..3218857	*mstX*	atypical membrane-integrating protein (Misticprotein)	0.351	0.52
BSU09690	complement(1044373..1044873)	*nhaX*	stress response protein, UspA family	0.350	0.41
BSU13789	1445314..1445541	*ykzQ*	putative peptidoglycan binding protein	0.350	0.28
BSU15320	1604771..1605490	*sigE*	RNA polymerase sporulation-specific sigma-29factor (sigma-E)	0.348	0.18
BSU32610	complement(3351110..3352096)	*frlB*	fructoselysine-6-P-deglycase	0.348	0.68
BSU21150	2236269..2237285	*yonB*	conserved hypothetical protein; phage SPbeta	0.348	0.96

BSU18520	2019797..2020567	*yoxB*	conserved hypothetical protein	0.347	0.33
BSU21130	2234233..2235669	*yonD*	conserved hypothetical protein; phage SPbeta	0.345	0.70
BSU13790	1445638..1446336	*ykvQ*	putative sporulation-specific glycosylase	0.345	0.26
BSU03990	451618..452739	*mtlD*	mannitol-1-phosphate dehydrogenase	0.344	0.57
BSU14970	1567651..1568049	*ylbD*	conserved hypothetical protein	0.344	0.23
BSU13160	1382020..1382430	*ohrB*	organic hydroperoxide resistance reductase B	0.342	0.42
BSU21329	complement(2247367..2247543)	*youB*	conserved hypothetical protein; phage SPbeta	0.342	0.35
BSU02840	306459..308144	*ycdG*	putative oligo-carbohydrate hydrolase	0.342	0.33
BSU06890	755907..756155	*cotJA*	component of the inner spore coat	0.342	0.29
BSU08590	929725..930585	*yfhM*	epoxide hydrolase	0.341	0.49
BSU03788	complement(430185..430274)	*yczM*	putative type I toxin	0.340	0.22
BSU03200	344551..345462	*putB*	proline oxidase	0.337	1.12
BSU39050	complement(4008143..4010203)	*katE*	catalase 2	0.337	0.35
BSU03210	345479..347026	*putC*	1-pyrroline-5-carboxylate dehydrogenase	0.336	1.02
BSU18280	complement(1958027..1959559)	*yngK*	putative exported protein	0.336	0.19
BSU22250	complement(2338017..2338394)	*yppG*	conserved hypothetical protein;methionine-glutamine-rich protein	0.335	0.24
BSU12150	complement(1284870..1285445)	*yjgB*	conserved hypothetical protein	0.335	0.26
BSU10940	1172650..1173336	*slpH*	2-phosphosulfolactate phosphatase	0.334	0.22
BSU23410	complement(2440775..2441791)	*spoVAD*	stage V sporulation protein AD	0.333	0.28
BSU40280	complement(4138800..4139537)	*yycO*	conserved hypothetical protein	0.333	0.33
BSU39660	complement(4072284..4072973)	*yxdJ*	two-component response regulator [YxdK]	0.332	1.39
BSU36090	3718794..3719129	*ywrE*	conserved hypothetical protein	0.331	0.50
BSU18220	complement(1951228..1952010)	*yngF*	putative Methylglutaconyl-CoA hydratase	0.331	0.31
BSU30470	complement(3119565..3119837)	*ytzC*	conserved hypothetical protein	0.329	0.23
BSU13190	complement(1386024..1386983)	*isp*	intracellular serine protease	0.329	0.25
BSU12170	1288541..1289101	*yjgD*	conserved hypothetical protein	0.329	0.19
BSU08980	975231..976409	*yhbH*	factor involved in shape determination(sporulation)	0.328	0.18
BSU36970	complement(3794676..3795350)	*spoIIR*	pro-sigma(E) endopeptidase (stage IIsporulation)	0.328	0.37
BSU10720	complement(1150206..1150427)	*gerPA*	spore germination protein	0.328	0.26
BSU06430	699093..700235	*purK*	N5-carboxyaminoimidazole ribonucleotidesynthase	0.328	0.71
BSU06290	complement(682375..683364)	*yeaA*	conserved hypothetical protein	0.328	0.59
BSU14568	complement(1526924..1527067)		putative antitoxin	0.325	0.22
BSU30840	3154007..3154726	*yteA*	putative DksA homolog	0.324	0.28
BSU03982	451185..451616	*mtlF*	phosphotransferase system (PTS)mannitol-specific enzyme IIA component	0.323	0.61
BSU02310	249979..251319	*ybfO*	putative exported hydrolase	0.322	0.56
BSU18210	complement(1949682..1951217)	*yngE*	putative methylcrotonoyl-CoA carboxylase	0.322	0.37
BSU15310	1603779..1604708	*spoIIGA*	protease processing pro-sigma-E	0.321	0.28
BSU14250	complement(1494403..1495368)	*yknT*	hypothetical protein	0.321	0.32
BSU39810	4088002..4089387	*csbC*	putative sugar transporter	0.321	0.43
BSU32280	3317502..3318002	*yutG*	putative phosphatidylglycerophosphatase A	0.321	0.29
BSU13169	1382457..1382627	*ykzN*	hypothetical protein	0.317	0.46
BSU06480	703237..705465	*purL*	phosphoribosylformylglycinamidine synthetase II	0.315	0.45
BSU21320	complement(2246656..2247342)	*yomL*	conserved hypothetical protein; phage SPbeta	0.315	0.27
BSU02600	282469..282897	*cwlJ*	cell wall hydrolase	0.314	0.24
BSU18670	complement(2037601..2038779)	*yoaN*	oxalate decarboxylase	0.311	0.41
BSU31930	3278325..3279461	*ald*	L-alanine dehydrogenase	0.311	1.08
BSU25820	complement(2658006..2658770)	*yqcI*	conserved hypothetical protein	0.309	0.25
BSU37030	3798789..3799343	*racA*	chromosome-anchoring protein RacA	0.309	0.31
BSU13020	complement(1371015..1371875)	*ykgA*	putative aminohydrolase	0.309	0.45
BSU40950	4204900..4205517	*yyaC*	conserved hypothetical protein	0.308	0.31
BSU25120	2593000..2593254	*yqfT*	conserved hypothetical protein	0.308	0.27
BSU30940	complement(3163735..3166131)	*glgP*	glycogen phosphorylase	0.307	0.30
BSU37310	complement(3831512..3832228)	*fnr*	transcriptional regulator (FNR/CAP family)	0.305	1.65
BSU03060	330771..332396	*lctP*	L-lactate permease	0.305	0.81
BSU34510	complement(3542179..3542691)	*yvdQ*	conserved hypothetical protein	0.304	0.24
BSU37210	3818906..3819178	*ywjC*	conserved hypothetical protein	0.301	0.45
BSU31020	complement(3182015..3182539)	*yuaF*	putative membrane integrity integral membraneprotein	0.301	0.55
BSU04220	473803..474225	*ydaG*	putative general stress protein	0.299	0.32
BSU17970	complement(1925655..1926047)	*cotM*	spore coat protein (outer)	0.298	0.22
BSU19700	complement(2140898..2141725)	*kamB*	epsilon-amino-beta-lysine acetyl transferase	0.294	0.25
BSU19330	2104056..2104901	*sodF*	superoxide dismutase	0.294	0.28
BSU30930	3163237..3163704	*ytaB*	putative receptor	0.292	0.35
BSU12420	1314453..1315724	*yjoB*	ATPase possibly involved in protein degradation	0.291	0.60
BSU27470	complement(2805704..2806228)	*yrrD*	conserved hypothetical protein	0.290	0.23
BSU32860	3374001..3374333	*yusN*	conserved hypothetical protein	0.289	0.24
BSU06900	756139..756402	*cotJB*	component of the inner spore coat	0.286	0.27
BSU06280	complement(681547..682362)	*ydjP*	putative peroxydase	0.282	0.61
BSU10670	complement(1148494..1148712)	*gerPF*	spore germination protein	0.281	0.23
BSU30980	complement(3169763..3171646)	*glgB*	1,4-alpha-glucan branching enzyme	0.279	0.27
BSU03680	417993..419747	*yclG*	putative uronase	0.278	0.17
BSU17030	1775067..1775612	*cotE*	morphogenic spore protein	0.278	0.16
BSU19670	complement(2138037..2138717)	*yodN*	conserved hypothetical protein	0.277	0.15
BSU31270	3212591..3213328	*tgl*	protein-glutamine gamma-glutamyltransferase(transglutaminase)	0.277	0.18
BSU03930	445344..446129	*gdh*	glucose 1-dehydrogenase	0.276	0.26
BSU29500	complement(3018309..3018764)	*ytfJ*	conserved hypothetical protein	0.274	0.20
BSU15510	1622657..1623751	*pyrAA*	pyrimidine-specific carbamoyl-phosphatesynthetase (small subunit, glutaminase subunit)	0.271	0.71
BSU38830	3986428..3987885	*aldY*	putative aldehyde dehydrogenase	0.270	0.42
BSU03981	449724..451160	*mtlA*	phosphotransferase system (PTS)mannitol-specific enzyme IICB component	0.269	0.47
BSU32580	complement(3347919..3348821)	*frlM*	fructose-amino acid permease	0.269	0.43
BSU22169	2329870..2330022	*ypzG*	conserved hypothetical protein	0.268	0.22
BSU27430	2802358..2803086	*glnQ*	glutamine ABC transporter (ATP-binding protein)	0.264	0.21
BSU00430	51680..52552	*yabG*	sporulation-specific protease	0.264	0.14
BSU24230	complement(2519102..2520382)	*spoIVB*	regulatory membrane-associated serine protease	0.263	0.27
BSU38320	3933209..3933595	*cidA*	holin regulator of murein hydrolases	0.261	0.99
BSU09140	complement(988417..988872)	*yhcM*	hypothetical protein	0.261	0.36
BSU06440	700232..701527	*purB*	adenylosuccinate lyase	0.260	0.55
BSU10790	1157237..1159081	*asnO*	asparagine synthetase	0.260	0.23
BSU27440	2803108..2803929	*glnH*	glutamine ABC transporter (glutamine-bindinglipoprotein)	0.258	0.17
BSU22070	complement(2319440..2320024)	*xpt*	xanthine phosphoribosyltransferase	0.257	0.81
BSU09770	complement(1050811..1052172)	*yheD*	spore coat associated protein	0.256	0.28
BSU08970	973156..975051	*prkA*	serine protein kinase	0.255	0.17
BSU06460	702319..702573	*purS*	factor required forphosphoribosylformylglycinamidine synthetase activity	0.255	0.39
BSU15490	1620476..1621390	*pyrB*	aspartate carbamoyltransferase	0.254	1.06
BSU37280	complement(3826259..3829945)	*narG*	nitrate reductase (alpha subunit)	0.254	1.96
BSU08490	complement(924210..924401)	*yfhD*	conserved hypothetical protein	0.253	0.29
BSU28060	complement(2863294..2864292)	*spoIIB*	spatial and temporal regulator of thedissolution of septal peptidoglycan during engulfment(stage II sporulation)	0.252	0.25
BSU30950	complement(3166118..3167572)	*glgA*	bacterial glycogen (starch) synthase	0.252	0.24
BSU09958	1071402..1071488	*sscA*	spore assembly and germination protein	0.251	0.17
BSU19600	2133455..2134156	*yodH*	putative S-adenosylmethionine-dependentmethyltransferase	0.249	0.21
BSU06370	694662..695984	*pbuG*	hypoxanthine/guanine permease	0.247	0.78
BSU00450	53183..53368	*sspF*	small acid-soluble spore protein(alpha/beta-type SASP)	0.247	0.18
BSU18240	complement(1953181..1954515)	*yngHA*	biotin carboxylase/methylcrotonoyl-CoAcarboxylase subunit	0.246	0.23
BSU38180	3918777..3919022	*ywzA*	conserved hypothetical protein	0.243	0.38
BSU06470	702570..703253	*purQ*	phosphoribosylformylglycinamidine synthetase I	0.242	0.46
BSU12050	complement(1277062..1277457)	*yjdH*	hypothetical protein	0.242	0.30
BSU30970	complement(3168624..3169766)	*glgC*	glucose-1-phosphate adenylyltransferase(ADP-glucose pyrophosphorylase) subunit alpha	0.239	0.24
BSU32130	3303042..3304022	*guaC*	GMP reductase	0.239	0.73
BSU00640	70538..73021	*spoIIE*	SpoIIAA-phosphate serine phosphatase	0.238	0.20
BSU33240	3410466..3411623	*oxdC*	oxalate decarboxylase	0.238	0.45
BSU06450	701601..702326	*purC*	phosphoribosylaminoimidazole succinocarboxamidesynthetase	0.238	0.48
BSU27469	complement(2805501..2805692)	*yrzR*	conserved hypothetical protein	0.238	0.22
BSU15500	1621374..1622660	*pyrC*	dihydroorotase	0.237	0.79
BSU27840	complement(2844675..2845838)	*safA*	morphogenetic protein associated with SpoVID	0.235	0.16
BSU18230	complement(1952031..1952930)	*yngG*	putative hydroxymethylglutaryl-CoA lyase	0.235	0.16
BSU40270	complement(4137626..4138789)	*yycP*	conserved hypothetical protein	0.231	0.24
BSU19710	complement(2141703..2143013)	*yodQ*	putative deacylase	0.231	0.28
BSU36040	complement(3714002..3714679)	*ywrJ*	conserved hypothetical protein	0.227	0.22
BSU39770	4084799..4085554	*iolR*	transcriptional regulator (DeoR family)	0.225	1.81
BSU22200	complement(2332784..2333011)	*cotD*	spore coat protein (inner)	0.224	0.14
BSU04370	complement(492654..492911)	*ydaS*	conserved hypothetical protein	0.219	0.28
BSU17410	complement(1872812..1873579)	*cwlC*	N-acetylmuramoyl-L-alanine amidase	0.218	0.12
BSU37270	complement(3824806..3826269)	*narH*	nitrate reductase (beta subunit)	0.217	1.87
BSU11139	complement(1190036..1190233)	*yizC*	conserved hypothetical protein; genus orphan	0.217	0.19
BSU18020	1930264..1930410	*sspN*	small acid-soluble spore protein	0.215	0.14
BSU30960	complement(3167569..3168600)	*glgD*	glucose-1-phosphate adenylyltransferase(ADP-glucose pyrophosphorylase) beta subunit	0.215	0.23
BSU09150	989022..989591	*yhcN*	putative lipoprotein	0.214	0.24
BSU37260	complement(3824226..3824780)	*narJ*	nitrate reductase molybdenum cofactor assemblychaperone NarJ	0.214	1.83

BSU10680	complement(1148744..1149145)	gerPE	spore germination protein	0.212	0.27
BSU37320	complement(3832327..3833514)	narK	nitrite extrusion permease	0.210	1.80
BSU00160	complement(23868..25151)	yaaH	spore peptidoglycan hydrolase	0.209	0.32
BSU09780	complement(1052162..1053253)	yheC	spore coat associated protein, similar to YheD	0.206	0.18
BSU13510	1417719..1417895	ykzE	conserved hypothetical protein	0.206	0.15
BSU31380	3224864..3225100	yuzA	conserved hypothetical protein	0.203	0.21
BSU29430	complement(3011555..3011689)	ytzD	hypothetical protein	0.203	0.16
BSU37290	complement(3830141..3830617)	arfM	transcriptional regulator	0.203	1.58
BSU04430	complement(495740..496561)	ydbD	putative manganese-containing catalase	0.200	0.22
BSU19810	2151626..2152045	ypqP	putative capsular polysaccharide biosynthesisenzyme fragment; N-terminal part of YpqP	0.200	0.11
BSU09390	complement(1013958..1015616)	ygxB	putative integral inner membrane protein	0.196	0.16
BSU04400	494506..494877	gsiB	general stress protein	0.194	0.27
BSU20190	2169807..2169926	yosA	putative type I toxin; phage SPbeta	0.193	0.17
BSU18260	complement(1956218..1957360)	yngJ	acyl-CoA dehydrogenase, short-chain specific	0.193	0.16
BSU10910	complement(1169877..1170476)	yisZ	putative adenylylsulfate kinase	0.190	0.15
BSU18250	complement(1954525..1956174)	yngI	putative acetoacetyl-CoA synthetase	0.187	0.14
BSU29630	complement(3032417..3033040)	refZ	regulator of FtsZ	0.184	0.24
BSU38070	complement(3906142..3906972)	sacT	transcriptional antiterminator	0.184	0.18
BSU32590	complement(3348825..3349703)	frlN	fructose-amino acid permease	0.183	0.40
BSU37820	complement(3883427..3884278)	spsK	putative dTDP-4-dehydrorhamnose reductase	0.183	0.12
BSU04200	472585..473088	ydaE	conserved hypothetical protein	0.181	0.31
BSU31000	complement(3179926..3180447)	yuaI	putative acetyl-transferase	0.180	0.60
BSU10430	complement(1117734..1118633)	yhxD	putative oxidoreductase	0.179	0.23
BSU22060	complement(2318127..2319443)	pbuX	xanthine permease	0.177	0.71
BSU37920	3893441..3893986	gerQ	inner spore coat protein	0.176	0.18
BSU19740	complement(2144356..2145690)	yodT	putative aminovalerate aminotransferase	0.175	0.22
BSU39670	complement(4073081..4073953)	iolJ	2-deoxy-5-keto-D-gluconic acid 6-phosphatealdolase	0.174	1.57
BSU37250	complement(3823558..3824229)	narI	nitrate reductase (gamma subunit)	0.172	1.69
BSU31140	3193863..3194348	cdoA	cysteine dioxygenase	0.170	0.12
BSU04190	471709..472569	ydaD	putative dehydrogenase	0.170	0.31
BSU31010	complement(3180465..3181994)	yuaG	putative flotillin-like protein	0.170	0.56
BSU39720	complement(4078173..4079066)	iolE	2-keto-myo-inositol dehydratase	0.169	1.83
BSU40530	4167110..4167592	cotF	spore coat protein	0.169	0.09
BSU07550	827455..827802	yflT	heat stress induced protein	0.167	0.24
BSU32640	3354066..3354212	sspG	small acid-soluble spore protein	0.166	0.10
BSU13780	1444099..1445298	ykvP	spore protein	0.166	0.18
BSU19610	2134244..2134495	yodI	conserved hypothetical protein	0.165	0.09
BSU05529	complement(599875..600105)	ydzR	conserved hypothetical protein	0.162	0.17
BSU28340	2898931..2899752	ysnF	putative stress response protein	0.161	0.19
BSU28100	complement(2869964..2870989)	ysxE	spore coat protein	0.161	0.16
BSU11280	1205165..1205899	yjaU	conserved hypothetical protein	0.160	0.13
BSU38330	3933577..3934254	ywbG	anti-holin factor controlling activity of mureinhydrolases	0.156	0.77
BSU37890	complement(3889732..3890901)	spsC	putative glutamine-dependent sugar transaminase	0.156	0.09
BSU37840	complement(3885239..3885979)	spsI	glucose-1-phosphate thymidylyltransferase	0.155	0.14
BSU37810	complement(3882979..3883434)	spsL	dTDP-4-deoxyrhamnose-3,5-epimerase	0.155	0.14
BSU23350	2435012..2435224	ypzD	conserved hypothetical protein	0.154	0.13
BSU37880	complement(3888862..3889731)	spsD	putative TDP-glycosamine N-acetyltransferase	0.154	0.06
BSU39710	complement(4076842..4078158)	iolF	inositol transport protein	0.153	1.78
BSU37440	complement(3843001..3844377)	ywhL	conserved hypothetical protein	0.153	0.15
BSU37830	complement(3884292..3885239)	spsJ	dTDP-glucose 4,6-dehydratase	0.151	0.10
BSU13509	1417561..1417716	ykzP	conserved hypothetical protein	0.150	0.13
BSU08800	complement(957705..959060)	ygaK	putative FAD-dependent oxido-reductase	0.149	0.14
BSU34530	3544642..3545604	cotR	spore coat protein assembly factor CotR	0.148	0.10
BSU32600	complement(3349761..3351029)	frlO	fructose amino acid-binding lipoprotein	0.147	0.37
BSU19510	complement(2124529..2124765)	yojB	conserved hypothetical protein	0.146	0.34
BSU09165	989712..990680	yhcO	hypothetical protein	0.145	0.07
BSU39740	complement(4081029..4082006)	iolC	2-deoxy-5-keto-D-gluconic acid kinase	0.144	2.04
BSU39690	complement(4074896..4075765)	iolH	putative sugar-phosphate epimerase/isomerase	0.143	1.73
BSU32850	complement(3372740..3373648)	putM	proline dehydrogenase 1	0.138	0.07
BSU25150	2595669..2596412	yqfQ	conserved hypothetical protein	0.136	0.10
BSU19490	2124021..2124494	gerT	component of the spore coat	0.135	0.11
BSU19690	complement(2139454..2140869)	kamA	lysine 2,3-aminomutase	0.134	0.11
BSU39700	complement(4075785..4076819)	iolG	myo-inositol 2-dehydrogenase/D-chiro-inositol3-dehydrogenase	0.134	1.77
BSU38750	complement(3976791..3977807)	cydB	cytochrome bb' ubiquinol oxidase (subunit II)	0.133	0.81
BSU39730	complement(4079083..4080996)	iolD	3D-(3,5/4)-trihydroxycyclohexane-1,2-dionehydrolase	0.131	1.94
BSU39680	complement(4073974..4074810)	iolI	inosose isomerase	0.130	1.72
BSU39750	complement(4082030..4082845)	iolB	5-deoxy-D-glucuronic acid isomerase	0.129	2.27
BSU06230	676442..677863	iolT	myo-inositol transporter	0.127	2.25
BSU09350	complement(1010984..1011226)	yhdB	conserved hypothetical protein	0.127	0.10
BSU38080	complement(3907012..3907314)	ywcI	hypothetical protein	0.127	0.14
BSU28410	complement(2904727..2904951)	gerE	transcriptional regulator	0.126	0.10
BSU30890	complement(3159258..3159689)	ytxO	outer spore coat protein	0.125	0.09
BSU37850	complement(3886004..3887023)	spsG	putative glycosyltransferase	0.123	0.06
BSU18030	1930447..1930698	tlp	small acid-soluble spore protein(thioredoxin-like protein)	0.122	0.07
BSU37900	complement(3890922..3892346)	spsB	putative dTDP glycosyl/glycerophosphatetransferase	0.120	0.06
BSU11799	1252815..1253021	yjzK	conserved hypothetical protein	0.119	0.07
BSU28110	complement(2871022..2872749)	spoVID	morphogenetic spore protein (stage VIsporulation)	0.117	0.12
BSU03789	complement(430356..430469)	yczN	putative spore and germination protein	0.116	0.05
BSU30880	3157961..3159184	ytcC	putative glucosyltransferase	0.115	0.08
BSU32650	3354212..3354487	yurS	hypothetical protein	0.114	0.07
BSU39760	complement(4082920..4084383)	mmsA	methylmalonate-semialdehyde dehydrogenase	0.114	2.19
BSU30870	3157008..3157958	ytcB	putative UDP-glucose epimerase	0.112	0.09
BSU11760	complement(1250656..1251174)	cotX	spore coat protein (insoluble fraction)	0.110	0.04
BSU37450	complement(3844416..3845771)	ywhK	factor interacting with DNA helicase PcrA	0.109	0.09
BSU14569	complement(1526859..1527017)	ykzV	putative type I toxin	0.109	0.09
BSU37860	complement(3887026..3887748)	spsF	putative glycosyltransferase	0.108	0.05
BSU37910	complement(3892351..3893121)	spsA	spore coat dTDP-glycosyltransferase	0.107	0.06
BSU11780	complement(1251631..1252017)	cotV	spore coat protein (insoluble fraction)	0.107	0.04
BSU30860	3155725..3157011	ytcA	putative UDP-glucose dehydrogenase	0.106	0.04
BSU16880	1760464..1760721	ymfJ	conserved hypothetical protein	0.106	0.12
BSU37870	complement(3887741..3888862)	spsE	putative phosphoenolpyruvate-sugarpyruvyltransferase	0.105	0.05
BSU34520	complement(3542943..3544286)	yvdP	spore coat protein; putative oxidoreductase	0.105	0.09
BSU17700	complement(1904995..1905195)	cotC	spore coat protein (outer)	0.098	0.13
BSU25710	complement(2648903..2649655)	cwlH	N-acetylmuramoyl-L-alanine amidase	0.095	0.12
BSU11770	complement(1251273..1251590)	cotW	spore coat protein (insoluble fraction)	0.095	0.05
BSU06850	753265..753702	yeeK	spore associated protein	0.095	0.10
BSU07310	802351..803286	yfnD	putative glycosyltransferase (complexcarbohydrate synthase)	0.094	0.04
BSU09180	complement(990612..991265)	yhcQ	conserved hypothetical protein	0.093	0.09
BSU10920	complement(1170473..1171642)	yitA	putative sulfate adenylyltransferase	0.091	0.10
BSU38740	complement(3975088..3976791)	cydC	ABC membrane transporter (ATP-binding protein)required for cytochrome bb' function	0.091	0.80
BSU06300	complement(683462..685003)	cotA	outer spore coat copper-dependent laccase	0.090	0.09
BSU29570	complement(3025445..3025654)	sspA	small acid-soluble spore protein (alpha-typeSASP)	0.089	0.08
BSU11810	1253385..1253639	spoVIF	sporulation-specific protein needed for heatresistance	0.087	0.03
BSU11809	1253103..1253252	yjcZ	putative type I toxin	0.087	0.04
BSU08660	937900..938154	sspE	small acid-soluble spore protein (gamma-typeSASP)	0.087	0.09
BSU16730	1744367..1745260	spoVFA	spore dipicolinate synthase subunit A	0.083	0.04
BSU36050	complement(3714739..3715881)	cotB	spore coat protein (outer)	0.082	0.08
BSU30910	complement(3160761..3161894)	cotSA	spore coat protein	0.082	0.05
BSU40660	complement(4179163..4180377)	yybF	putative permease	0.080	0.07
BSU36070	3717238..3717825	cotG	spore morphogenetic protein	0.079	0.04
BSU30920	3162084..3163157	cotI	spore coat kinase	0.079	0.04
BSU07290	800232..801143	yfnF	putative glycosyltransferase	0.077	0.03
BSU36030	3712617..3713945	ywrK	putative Na+/H+ antiporter	0.076	0.07
BSU07300	801172..802350	yfnE	putative glycosyltransferase (complexcarbohydrate synthase)	0.075	0.04
BSU07280	799240..800208	yfnG	putative CDP-sugar-dehydratase/epimerase	0.075	0.03
BSU17260	complement(1861384..1862712)	aprX	alkaline serine protease	0.073	0.05
BSU18440	complement(2008572..2010053)	gltB	glutamate synthase (small subunit)	0.073	0.39
BSU07270	798469..799233	yfnH	putative glucose-1-phosphatecytidylyltransferase	0.072	0.05
BSU38730	complement(3973364..3975091)	cydD	ABC membrane transporter (ATP-binding protein)required for cytochrome bb' function	0.069	0.87
BSU30900	complement(3159691..3160746)	cotS	spore coat protein	0.068	0.07
BSU16740	1745263..1745865	spoVFB	spore dipicolinate synthase subunit B	0.068	0.05
BSU19790	2149084..2150037	cgeB	protein involved in maturation of the outermostlayer of the spore	0.065	0.03
BSU11750	complement(1250016..1250504)	cotY	outer spore coat protein (insoluble fraction)	0.062	0.02
BSU39580	4065597..4065962	yxeE	inner spore coat protein	0.060	0.03
BSU19780	2148676..2149077	cgeA	spore outermost layer component	0.059	0.03
BSU17310	complement(1865512..1865787)	ymaG	inner spore coat protein; cell wall associatedprotein	0.054	0.04
BSU11740	complement(1249442..1249888)	cotZ	spore coat protein (insoluble fraction,outermost layer)	0.052	0.02

BSU09230	997175..997597	*yhcV*	putative oxidoreductase	**0.051**	**0.04**	
BSU27830	complement(2843931..2844527)	*coxA*	spore cortex protein	**0.047**	**0.04**	
BSU18450	complement(2010070..2014632)	*gltA*	glutamate synthase (large subunit)	**0.041**	**0.26**	
BSU12090	complement(1280626..1280874)	*cotT*	spore coat protein (inner coat)	**0.039**	**0.04**	
BSU25080	complement(2590282..2590671)	*yqfX*	conserved hypothetical protein	**0.039**	**0.02**	
BSU19770	complement(2148166..2148471)	*cgeC*	protein involved in maturation of the outermostlayer of the spore	**0.039**	**0.03**	
BSU_misc_RNA_35	complement(2331320..2331720)	*rnpB*		**0.039**	**0.78**	
BSU17670	complement(1901117..1901377)	*cotU*	spore coat protein	**0.027**	**0.06**	
BSU23970	complement(2491289..2491948)	*artQ*	High affinity arginine ABC transporter(permease)	**0.027**	**0.02**	
BSU23960	complement(2490574..2491296)	*artR*	High affinity arginine ABC transporter(ATP-binding protein)	**0.023**	**0.01**	
BSU09750	complement(1050031..1050234)	*sspB*	small acid-soluble spore protein (beta-typeSASP)	**0.022**	**0.07**	
BSU23980	complement(2492029..2492796)	*artP*	High affinity arginine ABC transporter bindinglipoprotein	**0.016**	**0.01**	
BSU11190	1195034..1196071	*argC*	N-acetylglutamate gamma-semialdehydedehydrogenase	**0.015**	**0.01**	
BSU11210	1197326..1198102	*argB*	N-acetylglutamate 5-phosphotransferase(acetylglutamate kinase)	**0.012**	**0.01**	
BSU11200	1196091..1197311	*argJ*	ornithine acetyltransferase; amino-acidacetyltransferase	**0.0102**	**0.01**	
BSU29450	complement(3013133..3014344)	*argG*	argininosuccinate synthase	**0.0062**	**0.00**	
BSU29440	complement(3011751..3013136)	*argH*	argininosuccinate lyase	**0.0056**	**0.00**	
BSU11250	1203461..1204420	*argF*	ornithine carbamoyltransferase	**0.0040**	**0.00**	
BSU11220	1198099..1199256	*argD*	N-acetylornithine aminotransferase	**0.0026**	**0.00**	
BSU11230	1199327..1200388	*carA*	arginine-specific carbamoyl-phosphate synthetase(small subunit)	**0.0019**	**0.00**	
BSU11240	1200381..1203473	*carB*	arginine-specific carbamoyl-phosphate synthetase(large subunit)	**0.0017**	**0.00**	

Table S2. Strains and plasmids used in this study.

Strain	Genotype	Reference or source
168	*trpC2*	Laboratory stock
OAM1124	*trpC2 dnaJ*::Tn (Km^r)	This study
NBS2001J	*trpC2 dnaJ* (Sp^r)	Unpublished (K Asai)
OAM959	*trpC2 thrC*::*sigX-lacZ* (Em^r) *rnpB*::Tn (Km^r)	11
OAM1125	*trpC2 rnpB*::Tn (Km^r)	This study
GP193	*rny*- Pxyl-*rny* (Cm^r)	12
OAM962	*trpC2 thrC*::*sigX-lacZ* (Em^r) *rnpB*-Pspac-*rnpB* (Em^r Tc^r)	11
OAM821	*trpC2 proB*::*lacZ* (Tc^r)	6
OAM1126	*trpC2 proB*::*lacZ* (Tc^r) *dnaJ*::Tn (Km^r)	This study
OAM1127	*trpC2 proB*::*lacZ* (Tc^r) *dnaK*::Tn (Km^r)	This study
OAM1128	*trpC2 proB*::*lacZ* (Tc^r) *grpE*::Tn (Km^r)	This study
OAM1129	*trpC2 proB*::*lacZ* (Tc^r) *nusA*::Tn (Km^r)	This study
OAM1130	*trpC2 proB*::*lacZ* (Tc^r) *nusA*::Tn (Km^r) *amyE*::Pxyl-*ylxR* (Cm^r)	This study
OAM1131	*trpC2 proB*::*lacZ* (Tc^r) *cshA*::Tn (Km^r)	This study
OAM1132	*trpC2 proB*::*lacZ* (Tc^r) *ptsG*::Tn (Km^r)	This study
OAM1133	*trpC2 proB*::*lacZ* (Tc^r) *pykA*::Tn (Km^r)	This study
OAM1134	*trpC2 proB*::*lacZ* (Tc^r) *xtmB*::Tn (Km^r)	This study
OAM1135	*trpC2 proB*::*lacZ* (Tc^r) *flhB*::Tn (Km^r)	This study
OAM822	*trpC2 proB*::*lacZ* (Tc^r) *ylxR*::Tn (Km^r)	10
OAM1136	*trpC2 proB*::*lacZ* (Tc^r) *rnpB*::Tn (Km^r)	This study
OAM1220	*trpC2 proB*::*lacZ* (Tc^r) *tig*::pUKM504 (Km^r)	This study
OAM1137	*trpC2 proB*::*lacZ* (Tc^r) *rnpB*::Tn (Km^r) *dnaJ* (Sp^r)	This study
OAM1138	*trpC2 proB*::*lacZ* (Tc^r) *dnaJ*::Tn (Km^r) *amyE*::Pxyl-*dnaJ* (Cm^r)	This study
OAM841	*trpC2 proB*::*lacZ* (Tc^r) *ylxR*::Tn (Km^r) *amyE*::Pxyl-*ylxR* (Cm^r)	10
OAM1139	*trpC2 proB*::*lacZ* (Tc^r) *amyE*::Pxyl-*ylxR* (Cm^r)	This study
OAM1141	*trpC2 proB*::*lacZ* (Tc^r) *dnaJ*::Tn (Km^r) *amyE*::Pxyl-*ylxR* (Cm^r)	This study
OAM1140	*trpC2 proB*::*lacZ* (Tc^r) *rny*-Pxyl-*rny* (Cm^r)	This study
OAM1142	*trpC2 proB*::*lacZ* (Tc^r) *rny*-Pxyl-*rny* (Cm^r) *rnpB*::Tn (Km^r)	This study
OAM1143	*trpC2 proB*::*lacZ* (Tc^r) *rny*-Pxyl-*rny* (Cm^r) *dnaJ*::Tn (Km^r)	This study
OAM1144	*trpC2 proB*::*lacZ* (Tc^r) *rnpB* (Em^r)-Pspac-*rnpB* (*lacZ*::Tc^r)	This study
OAM1145	*trpC2 proB*::*lacZ* (Tc^r) *rnpB* (Em^r)-Pspac-*rnpB* (*lacZ*::Tc^r) *dnaJ*::Tn (Km^r)	This study
OAM1150	*trpC2 miaB* (Em^r)	This study
OAM1151	*trpC2 proB*::*lacZ* (Tc^r) *miaB* (Em^r)	This study
OAM1152	*trpC2 proB*::*lacZ* (Tc^r) *dnaJ*::Tn (Km^r) *miaB* (Em^r)	This study
YABBd	*trpC2 yabB* (Em^r *lacZ*)	This study
OAM1153	*trpC2 proB*::*lacZ* (Tc^r) *yabB* (Em^r *lacZ*::Tc^r)	This study
OAM1154	*trpC2 proB*::*lacZ* (Tc^r) *dnaJ*::Tn (Km^r) *yabB* (Em^r *lacZ*::Tc^r)	This study
YQFNd	*trpC2 trmK* (Em^r *lacZ*)	This study
OAM1155	*trpC2 proB*::*lacZ* (Tc^r) *trmK* (Em^r *lacZ*::Tc^r)	This study
OAM1156	*trpC2 proB*::*lacZ* (Tc^r) *trmK* (Em^r *lacZ*::Tc^r) *dnaJ*::Tn (Km^r)	This study
YQEVd	*trpC2 mtaB* (Em^r *lacZ*)	This study
OAM1157	*trpC2 proB*::*lacZ* (Tc^r) *mtaB* (Em^r *lacZ*::Tc^r)	This study
OAM1158	*trpC2 proB*::*lacZ* (Tc^r) *mtaB* (Em^r *lacZ*::Tc^r) *dnaJ*::Tn (Km^r)	This study
OAM1159	*trpC2 proB*::*lacZ*-1 (Tc^r)	This study
OAM1160	*trpC2 proB*::*lacZ*-1 (Tc^r) *dnaJ*::Tn (Km^r)	This study
OAM1161	*trpC2 proB*::*lacZ*- 1 (Tc^r) *ylxR*::Tn (Km^r)	This study
OAM1162	*trpC2 proB*::*lacZ*- 1 (Tc^r) *rnpB*::Tn (Km^r)	This study
OAM1221	*trpC2 proB*::*lacZ*-2 (Tc^r)	This study
OAM1222	*trpC2 proB*::*lacZ*-2 (Tc^r) *dnaJ*::Tn (Km^r)	This study
OAM1223	*trpC2 proB*::*lacZ*- 2 (Tc^r) *ylxR*::Tn (Km^r)	This study
OAM1224	*trpC2 proB*::*lacZ*- 2 (Tc^r) *rnpB*::Tn (Km^r)	This study
OAM1163	*trpC2 amyE*::P*proBA*-*lacZ* (Cm^r)	This study
OAM1164	*trpC2 amyE*::P*proBA*-*lacZ* (Cm^r) *ylxR*::Tn (Km^r)	This study
OAM1165	*trpC2 amyE*::P*proBA*-*lacZ* (Cm^r) *dnaJ*::Tn (Km^r)	This study
OAM1166	*trpC2 amyE*::P*proBA*-*lacZ* (Cm^r) *rnpB*::Tn (Km^r)	This study
OAM1167	*trpC2 amyE*::P*proBA*-*lacZ*-2 (Cm^r::Tc^r)	This study
OAM1168	*trpC2 amyE*::P*proBA*-*lacZ*-2 (Cm^r::Tc^r) *rny*- Pxyl-*rny* (Cm^r)	This study
YODFd	*trpC2 yodF*-*lacZ* (Em^r)	13
OAM1169	*trpC2 yodF*-*lacZ* (Em^r) *dnaJ* (Sp^r)	This study
OAM1170	*trpC2 yodF*-*lacZ* (Em^r) *ylxR* (Km^r)	This study
OAM1224	*trpC2 yodF*-*lacZ* (Em^r) *tig*:: pUKM504 (Km^r)	This study
OAM1171	*trpC2 yodF*-*lacZ* (Em^r) *rnpB*::Tn (Km^r)	This study
OAM1172	*trpC2 yodF*-*lacZ* (Em^r) *rnpB*::Tn (Km^r) *dnaJ* (Sp^r)	This study
OAM1173	*trpC2 yodF*-*lacZ* (Em^r) *amyE*::Pxyl-*ylxR* (Cm^r)	This study
OAM1174	*trpC2 yodF*-*lacZ* (Em^r) *amyE*::Pxyl-*ylxR* (Cm^r) *dnaJ*::Tn (Km^r)	This study
OAM1175	*trpC2 yodF*-*lacZ* (Em^r) *amyE*::Pxyl-*ylxR* (Cm^r) *rnpB*::Tn (Km^r)	This study
OAM1176	*trpC2 amyE*::P*yoyD*-*lacZ* (Cm^r)	This study
OAM1177	*trpC2 amyE*::P*yoyD*-*lacZ* (Cm^r) *ylxR*::Tn (Km^r)	This study
OAM1178	*trpC2 amyE*::P*yoyD*-*lacZ* (Cm^r) *dnaJ* (Sp^r)	This study
OAM1179	*trpC2 amyE*::P*yoyD*-*lacZ* (Cm^r) *rnpB*::Tn (Km^r)	This study
OAM1180	*trpC2 besA*-*lacZ* (Em^r)	This study
OAM1181	*trpC2 besA*-*lacZ* (Em^r) *dnaJ*::Tn (Km^r)	This study
OAM1182	*trpC2 besA*-*lacZ* (Em^r) *ylxR*::Tn (Km^r)	This study
OAM1183	*trpC2 besA*-*lacZ* (Em^r) *rnpB*::Tn (Km^r)	This study
OAM1184	*trpC2 amyE*::P*besA*-*lacZ* (Cm^r)	This study
OAM1185	*trpC2 amyE*::P*besA*-*lacZ* (Cm^r) *ylxR*::Tn (Km^r)	This study

OAM1186	*trpC2 amyE*::P*besA*-*lacZ* (Cm^r) *dnaJ*::Tn (Km^r)	This study
OAM1187	*trpC2 amyE*::P*besA*-*lacZ* (Cm^r) *rnpB*::Tn (Km^r)	This study
YYDGd	*trpC2 epeE*-*lacZ* (Em^r)	13
OAM1188	*trpC2 epeE*-*lacZ* (Em^r) *dnaJ*::Tn (Km^r)	This study
OAM1189	*trpC2 epeE*-*lacZ* (Em^r) *ylxR*::Tn (Km^r)	This study
OAM1190	*trpC2 epeE*-*lacZ* (Em^r) *rnpB*::Tn (Km^r)	This study
OAM1191	*trpC2 amyE*::P*epeX*-*lacZ* (Cm^r)	This study
OAM1192	*trpC2 amyE*::P*epeX*-*lacZ* (Cm^r) *ylxR*::Tn (Km^r)	This study
OAM1193	*trpC2 amyE*::P*epeX*-*lacZ* (Cm^r) *dnaJ*::Tn (Km^r)	This study
OAM1194	*trpC2 amyE*::P*epeX*-*lacZ* (Cm^r) *rnpB*::Tn (Km^r)	This study
OAM1039	*trpC2 amyE*::P*argGH*-*lacZ* (Cm^r)	14
OAM1195	*trpC2 amyE*::P*argGH*-*lacZ* (Cm^r) *ylxR*::Tn (Km^r)	This study
OAM1196	*trpC2 amyE*::P*argGH*-*lacZ* (Cm^r) *dnaJ*::Tn (Km^r)	This study
OAM1197	*trpC2 amyE*::P*argGH*-*lacZ* (Cm^r) *rnpB*::Tn (Km^r)	This study
JH642 (ϕCMIVCA)	*trpC2 pheA1 spoIVCA*-*lacZ* (Cm^r) [ϕCMIVCA]	15
OAM1198	*trpC2 pheA1 spoIVCA*-*lacZ* (Cm^r) [ϕCMIVCA] *dnaJ*::Tn (Km^r)	This study
OAM1199	*trpC2 pheA1 spoIVCA*-*lacZ* (Cm^r) [ϕCMIVCA] *ylxR*::Tn (Km^r)	This study
OAM1200	*trpC2 pheA1 amyE*::P*spoIVCA*-*lacZ* (Cm^r)	This study
OAM1201	*trpC2 pheA1 amyE*::P*spoIVCA*-*lacZ* (Cm^r) *dnaJ*::Tn (Km^r)	This study
YXJAd	*trpC2 nupG*-*lacZ* (Em^r)	13
OAM1202	*trpC2 nupG*-*lacZ* (Em^r) *dnaJ*::Tn (Km^r)	This study
OAM1203	*trpC2 nupG*-*lacZ* (Em^r) *ylxR*::Tn (Km^r)	This study
OAM1204	*trpC2 amyE*::P*nupG*-*lacZ* (Cm^r)	This study
OAM1205	*trpC2 amyE*::P*nupG*-*lacZ* (Cm^r) *dnaJ*::Tn (Km^r)	This study
OAM1206	*trpC2 proB*-FLAG (Cm^r)	This study
OAM1207	*trpC2 proB*-FLAG (Cm^r) *dnaJ*::Tn (Km^r)	This study
OAM1208	*trpC2 citM*-FLAG (Cm^r)	This study
OAM1209	*trpC2 citM*-FLAG (Cm^r) *dnaJ*::Tn (Km^r)	This study
OAM1210	*trpC2 spoIVCA*-FLAG (Cm^r)	This study
OAM1211	*trpC2 spoIVCA*-FLAG (Cm^r) *dnaJ*::Tn (Km^r)	This study
OAM1212	*trpC2 nupG*-FLAG (Cm^r)	This study
OAM1213	*trpC2 nupG*-FLAG (Cm^r) *dnaJ*::Tn (Km^r)	This study
ASK216	*trpC2 amyE*::*spoIID*-*lacZ* (Cm^r)	16
OAM1214	*trpC2 amyE*::*spoIID*-*lacZ* (Cm^r) *dnaJ*::Tn (Km^r)	This study
168::pPS918	*trpC2 amyE*::*sspE*-*lacZ* (Cm^r)	17
OAM1215	*trpC2 amyE*::*sspE*-*lacZ* (Cm^r) *dnaJ*::Tn (Km^r)	This study
ASK218	*trpC2 amyE*::*cotA*-*lacZ* (Cm^r)	18
OAM1216	*trpC2 amyE*::*cotA*-*lacZ* (Cm^r) *dnaJ*::Tn (Km^r)	This study
PY79 derivative	*amyE*::*cotD*-*lacZ* (Cm^r) *skf*::Tc^r	19
OAM1218	*trpC2 amyE*::*cotD*-*lacZ* (Cm^r)	This study
OAM1219	*trpC2 amyE*::*cotD*-*lacZ* (Cm^r) *dnaJ*::Tn (Km^r)	This study
Plasmid	Description	
pMarA	Amp^r Em^r Km^r	20
pX	Amp^r *amyE*::*xylR*-Pxyl Cm^r	1
pX-dnaJ	Px carrying *dnaJ* (*dnaJ* ORF with its SD), Cm^r	This study
pMutinIII	Insertion vecter, ampicillin and erythromycin resistance, *lacZI*	2
pMutin-besA	pMutinIII carrying P*besA* and a part of *besA* ORF	This study
pMUTIN-His	Amp^r Em^r His-tag Pspac	3
pMUTIN-His-miaB	Amp^r Em^r, a part of *miaB,* His-tag Pspac	This study
pSK10Δ6-Tc	Tc^r '*lacZ*	6
pSPB106	Tc^r *proB* (305th codon) ::*lacZ*	6
pSPB108	Tc^r *proB* (68th codon) ::*lacZ*	This study
pSPB107	Tc^r *proB* (2th codon) ::*lacZ*	This study
pIS284	Amp^r Cm^r *amyE*	4
pIS284-proB	pIS284 carrying *proB* (-500 to -1 relative to the translation start site)	This study
pIS284-yoyD	pIS284 carrying *yoyD* (-237 to -1 relative to the translation start site)	This study
pIS284-besA	pIS284 carrying *besA* (-292 to -1 relative to the translation start site)	This study
pIS284-epeX	pIS284 carrying *epeX* (-360 to -1 relative to the translation start site)	This study
pUKM504	pUC19 carrying Km^r	7
pUKM504-tig	pUKM504 carrying partial ORF of *tig*	This study
pLacZ::Tc	Amp^r *lacZ*::Tc^r	Gift from Asai K
ECE75	Amp^r, Cm^r::Tc^r	BGSC
pCA3xFLAG	Amp^r, FLAG, Cm^r	5
pproB-flag	pCA3xFLAG carrying C-terminal region of *proB*	This study
pcitM-flag	pCA3xFLAG carrying C-terminal region of *citM*	This study
pspoIVCA-flag	pCA3xFLAG carrying C-terminal region of *spoIVCA*	This study
pnupG-flag	pCA3xFLAG carrying C-terminal region of *nupG*	This study
pGBT9	Amp^r, Trp1, Gal4DBD	Clontech
pGBT9-dnaK	Amp^r, Trp1, Gal4DBD-*dnaK*	This study
pGBT9-grpE	Amp^r, Trp1, Gal4DBD-*grpE*	This study
pGAD-C1	Amp^r, Leu2, Gal4AD	8
pGAD-C2	Amp^r, Leu2, Gal4AD	8
pGAD-C3	Amp^r, Leu2, Gal4AD	8

Table S3. Oligonucleotides used for this study.

Name	Sequence	Product/use
695	5'-GCTTGTAAATTCTATCATAATTG-3'	Transposon mutagenesis
696	5'-AGGGAATCATTTGAAGGTTGG-3'	Transposon mutagenesis
pX-dnaJ-Spe	5'-AAACTAGTAAATTCGGGAGAGTGAAGCG-3'	pX-dnaJ
pX-dnaJ-Bam	5'-ATGGGATCCTTAATCGCCTTTAAACGCGCG-3'	pX-dnaJ
pMut-besA-F-E	5'-TTGGAATTCACCCTGGCTTTTGGGAG-3'	pMUTIN-besA, pIS-besA
pMut-besA-R-B	5'-ATCGGATCCCTCCGCTCCGCCATG-3'	pMUTIN-besA
pUKM-miaB-F-E	5'-CGCGAATTCCGCCTTCTTTAAAAGACGC-3'	pMUTIN-His-miaB
pUKM-miaB-R-Xh	5'-GCGCTCGAGGCGATGAATGTTATGCGTTC-3'	pMUTIN-His-miaB
pUKM-tig-F-E	5'-CGCGAATTCAATTCCTGGATTCCGTAAAGG-3'	pUKM504-tig
pUKM-tig-R-Xh	5'-GCGCTCGAGTAGTTTTCAGCTTTTCCGC-3'	pUKM504-tig
pIS-proB-F-E	5'-ATTGAATTCGATATTGAGCGTGTGGACCACCGCG-3'	pIS284-proB
pIS-proB-R-B	5'-TTCGGATCCATACGCGTTTCGATACCGTTCT-3'	pIS284-proB
pIS-yodF-F-E	5'-TTGGAATTCTTGCGGATCTCTGGCG-3'	pIS284-yoyD
pIS-yodF-R-B	5'-ATCGGATCCTATTCAAGTCCTCCATCTTTTTTTG-3'	pIS284-yoyD
pIS-besA-R-B	5'-ATCGGATCCGCAGTACGACATTCCTCTCC-3'	pIS284-besA
pIS-yydF-E	5'-ATCGAATTCCGCATAACTG CTTTTAGAG-3'	pIS284-epeX
pIS-yydF-B	5'-ATCGGATCCATTATCCCTCCTCCTTTTCTAA-3	pIS284-epeX
Pflag-proB-F-E	5'-TTAGAATTCAGCTGGATCAAAGGTCG-3'	pproB-flag
Pflag-proB-R-Xb	5'-GATCTAGAGTCTTTTACATTGACCCAGT-3'	pproB-flag
Pflag-citM-F-E	5'-TTAGAATTCTTTAATAGGATGTTTAGTATCGGG-3'	pcitM-flag
Pflag-citM-R-Xb	5'-GATCTAGATACGGAAATAGAGATCGCACCG-3'	pcitM-flag
Pflag-nupG-F-E	5'-TTAGAATTCTAACAGTATGCTTGTCGG-3'	pnupG-flag
Pflag-nupG-R-Xb	5'-GATCTAGACCACACAAACAGGCCGAC-3'	pnupG-flag
Pflag-spoIVCA-F-E	5'-TTAGAATTCTGGCGCAAAGGACC-3'	pspoIVCA-flag
Pflag-spoIVCA-R-Xb	5'-GATCTAGAATACGTTTCAATATATATGGTG-3'	pspoIVCA-flag
pSK-ProB-E	5'-AAAGAATTCATTATGTGACAAACGATCTTG-3'	pSPB107, pSPB108
pSK-ProB-H1	5'-ATGAAGCTTTTCATTTATTCTCCTCCGCGG-3'	pSPB107
pSK-ProB-H2	5'-ATGAAGCTTTGATGGTAACGGGACGG-3'	pSPB108
dnaK5'	5'-TTGGATCCGTAGTAAAGTTATCGGAATC-3'	pGBT9-dnaK
dnaK3'	5'-TTGGATCCTTATTTTTTGTTTTGGTCGTCGTT-3'	pGBT9-dnaK
grpE5'	5'-TTGGATCCGTTCAGAAGAAAAACAAACCGTTG-3'	pGBT9-grpE
grpE3'	5'-TTGGATCCTTATTGATTCACTTTGACCATGGA-3'	pGBT9-grpE

Fig. S1. Expression of *proB*::*lacZ-1* and *proB*::*lacZ-2*. β-Galactosidase activities are shown in Miller units. Data represents means ± standard deviations of three independent experiments. The *x*-axis represents the growth time in h relative to the end of vegetative growth (T0). Cells were grown in SM. Significant differences for some data points were determined using nonpaired t-test and are shown in the panel. *$P < 0.05$. Substrate CPRG was used. OAM1159 and OAM1221, wild type; OAM1161 and OAM1223, *ylxR*(*rnpM*); OAM1160 and OAM1222, *dnaJ*; OAM1162 and OAM1224, *rnpB*. Schematic representation of fusion structure is shown alongside the panel. Box and bent arrows show open reading frame and promoter, respectively. Number in parenthesis indicates promoter region relative to the translation start site. “aa”, amino acid.

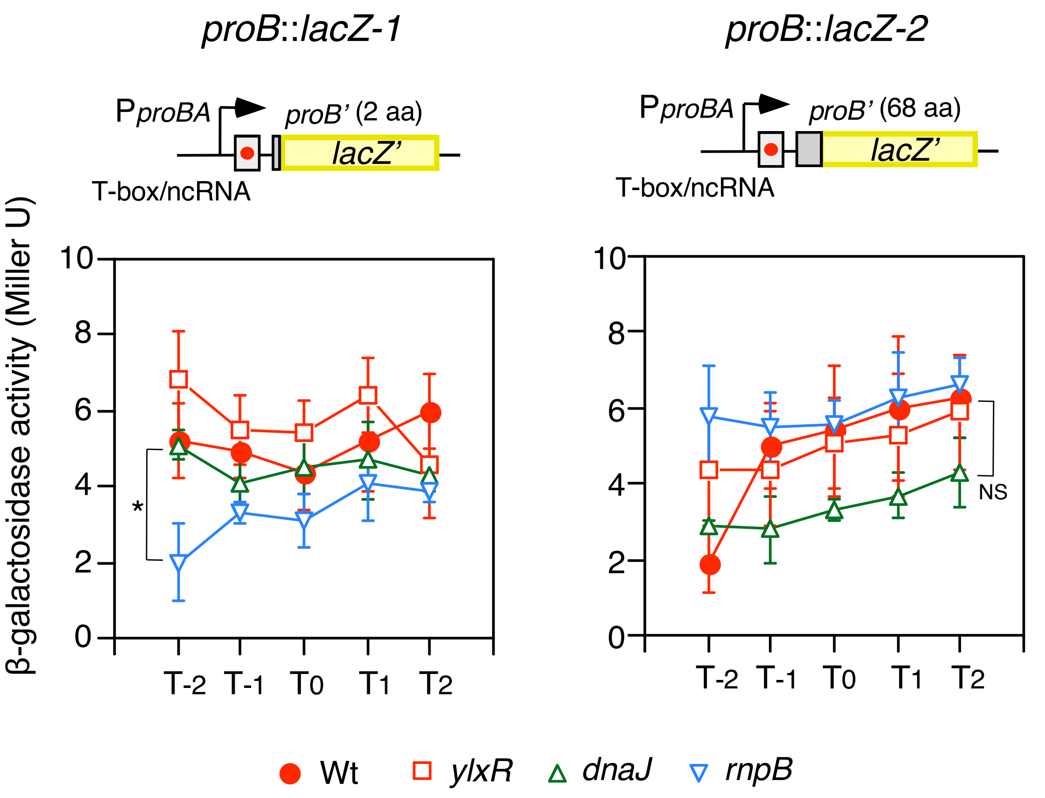

Fig. S1. Expression of *proB*::*lacZ-1* and *proB*::*lacZ-2*.

Fig S2. Characterization of *ylxR*(*rnpM*), *dnaJ* and *rnpB* strain. (**A**) Schemes of glucose-response system. Arrows and T-bar indicate activation. and inhibition, respectively. The dotted line shows a multi-step reaction. The regulatory cascade constitutes two pathways. i) Feedback regulation including *ylxR*. Association of CshA with RNAP (21), glucose-stimulated CshA acetylation and *ylxR* expression (22), CshA-dependent P*ylxS* regulation driving YlxR expression, which regulates metabolic genes including *proB* (10), and transcriptional regulation of *tsaEBD* through YlxR-binding to the promoter of *tsaEBD*, whose products are assembled and regulate translation of pyruvate dehydrogenase (PDH) (23). Pyruvate dehydrogenase provides acetyl-CoA, which would be the acetyl moiety source for CshA acetylation. ii) Glucose-stimulated response. Incorporated glucose finally upregulates manganese incorporation through transcriptional regulation of manganese transporter genes (14), leading to activation of *ywlE* encoding a protein arginine phosphatase. This response stabilizes proteins including PDH. Finally, *rnpB*::Tn causes stringent response, which downregulates PDH-encoding genes (24). Ac, acetyl moiety; RNAP, RNA polymerase. (**B**) Expression of *proB*::*lacZ* under various temperature conditions. β-Galactosidase activities are shown in Miller units. Data represents means ± standard deviations of three independent experiments. The *x*-axis represents the growth time in h relative to the end of vegetative growth (T0). Cells were grown in SM. Substrate CPRG was used. OAM821, wild type; OAM1126, *dnaJ.* (**C**) Summary of RNA-seq results for PDH. Data from Table S1 and Ref 10. (**D**) Schematic model for YlxR(RnpM) function as non-specific DNA binding protein in the presence of glucose. (**E**) Cell growth profiles in various mutants. Typical cell growth profiles in SM monitored with a Klett calorimeter (Fisher Scientific, Waltham, MA, USA) are shown. OAM1163, wild type; OAM1164, *ylxR*(*rnpM*); OAM1165, *dnaJ*; OAM1166, *rnpB*.

A

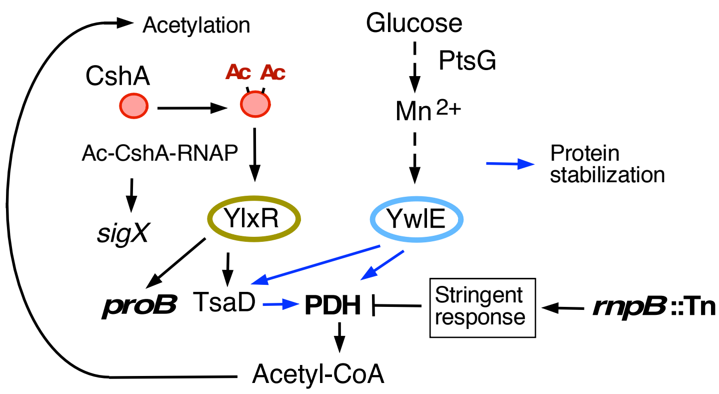

B

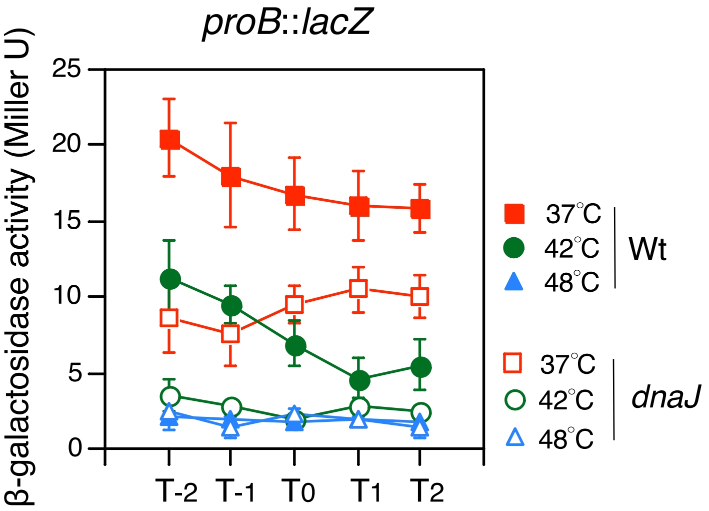

C

Gene	RNA-seq	
	ylxR vs Wt (2% glucose)	*rnpB* vs Wt (No glucose)
pdhA	1.23	0.44
pdhB	1.32	0.38
pdhC	0.82	0.47
pdhD	0.87	0.47

D

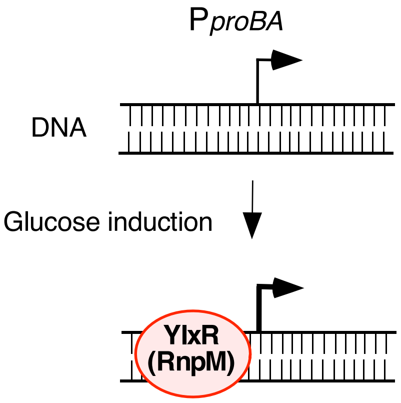

E

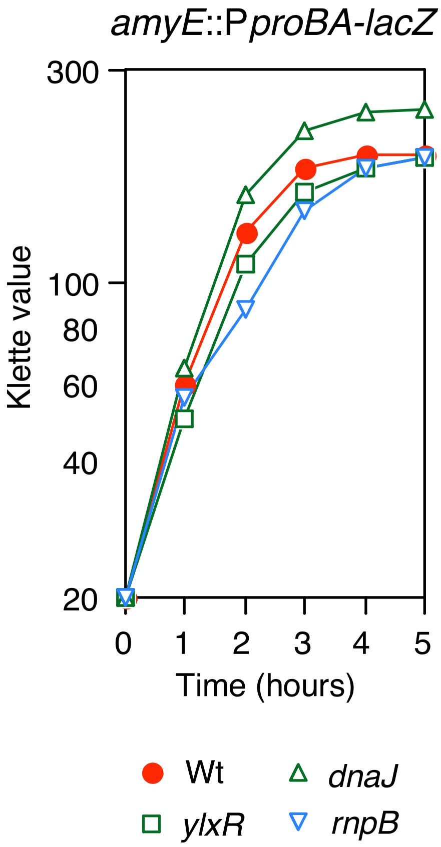

Fig. S2. Characterization of *ylxR* (*rnpM*), *dnaJ*, and *rnpB* strains.

Fig. S3. Expression of *besA, epeXE* and P*argGH*. β-Galactosidase activities are shown in Miller units. Data represents means ± standard deviations of three independent experiments. The *x*-axis represents the growth time in h relative to the end of vegetative growth (T0). Cells were grown in SM. Significant differences for some data points were determined using nonpaired t-test and are shown in the panel. *P < 0.05; NS, not significant. Substrate CPRG was used for *besA* and *epeXE*. Substrate ONPG was used for P*argGH*. Schematic representations of fusion structure are shown aside or above the panel. Box and bent arrows show open reading frame and promoter, respectively. Number in parenthesis indicates promoter region relative to the translation start site. "aa", amino acid. (**A**) Summary of RNA-seq results of the selected genes for LacZ analysis. For *rnpB* and *dnaJ,* see Table S1. For *ylxR,* culture conditions are different from the cases for *rnpB* and *dnaJ* and see Ref 10. Vertical lines show genes which constitute an operon. **(B)** Expression of transcriptional P*argGH-lacZ* fusion in various genetic backgrounds. OAM1039, wild type; OAM1195, *ylxR*(*rnpM*); OAM1196, *dnaJ*; OAM1197, *rnpB*. (**C**) Expression of transcriptional P*besA-lacZ* and *besA-lacZ* fusions in various genetic backgrounds. Left, OAM1184, wild type; OAM1185, *ylxR*(*rnpM*); OAM1186, *dnaJ*; OAM1187, *rnpB*. Right, OAM1180, wild type; OAM1182, *ylxR*(*rnpM*); OAM1181, *dnaJ*; OAM1183, *rnpB*. **(D)** Expression of transcriptional P*epeX-lacZ* and *epeE-lacZ* fusions in various genetic backgrounds. Left, OAM1191, wild type; OAM1192, *ylxR*(*rnpM*); OAM1193, *dnaJ*; OAM1194, *rnpB*. Right, YYDGd, wild type; OAM1189, *ylxR*(*rnpM*); OAM1188, *dnaJ*; OAM1190, *rnpB*.

A

Gene \ Fold	RNA-seq *rnpB* vs Wt (No glucose)	*dnaJ* vs Wt (No glucose)	*ylxR* vs Wt (2% glucose)
proB	3.25	1.20	0.15
proA	2.58	1.20	0.16
yoyD	2.96	1.60	2.57
yodF	2.85	1.69	2.15
besA	2.31	0.27	1.20
dhbA	1.98	0.14	1.41
dhbC	1.80	0.12	1.94
dhbE	1.72	0.11	1.83
dhbB	1.65	0.09	1.75
dhbF	1.64	0.08	2.40
epeX (yydF)	3.47	0.30	9.12
epeE (yydG)	2.41	0.44	3.43
epeP (yydH)	1.50	0.51	3.12
argG	0.01	0.00	85.68
argH	0.01	0.00	98.98

B

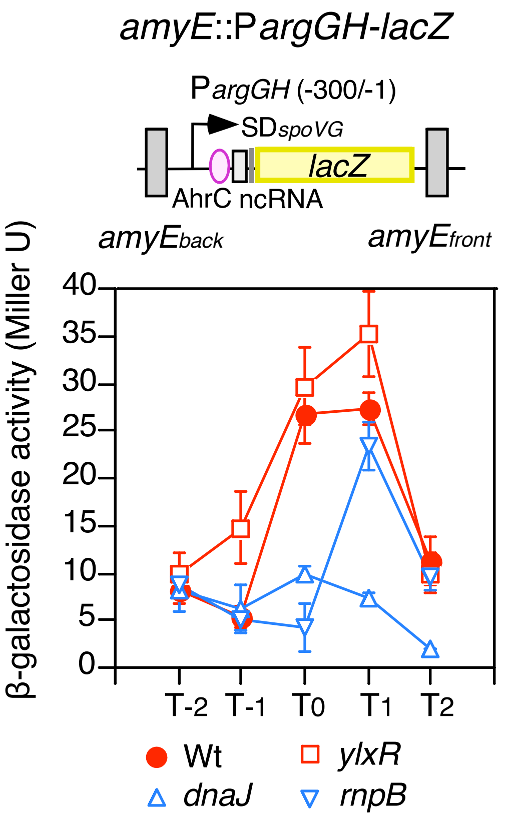

C

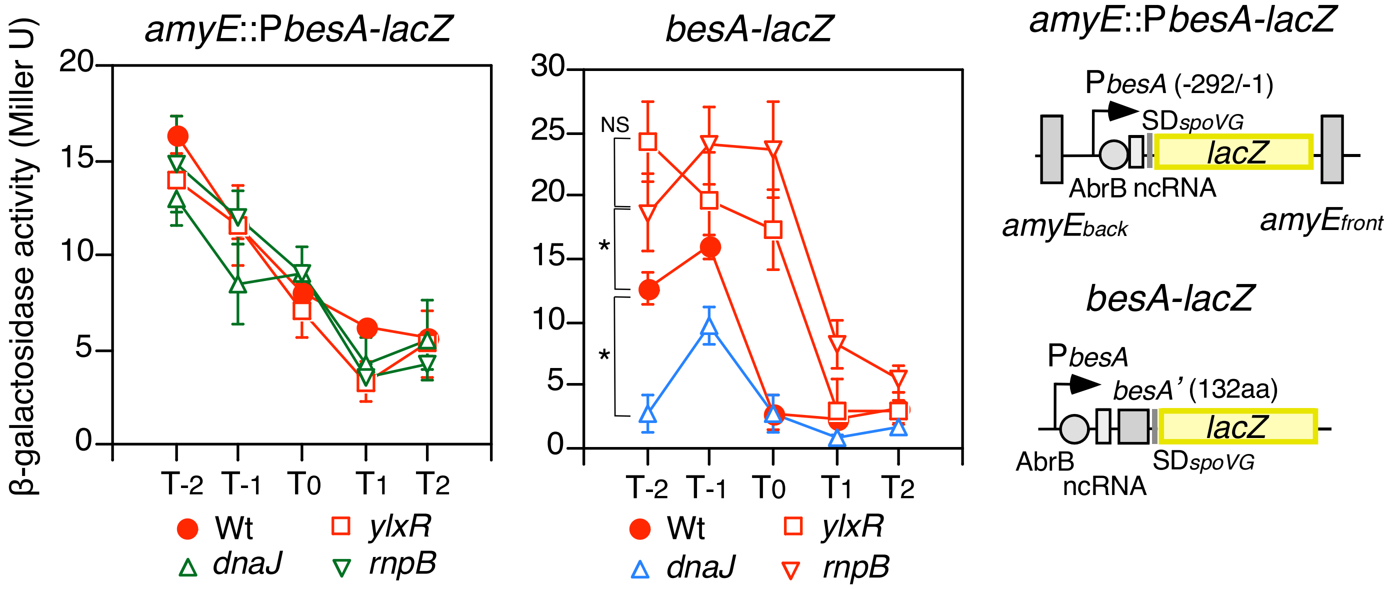

D

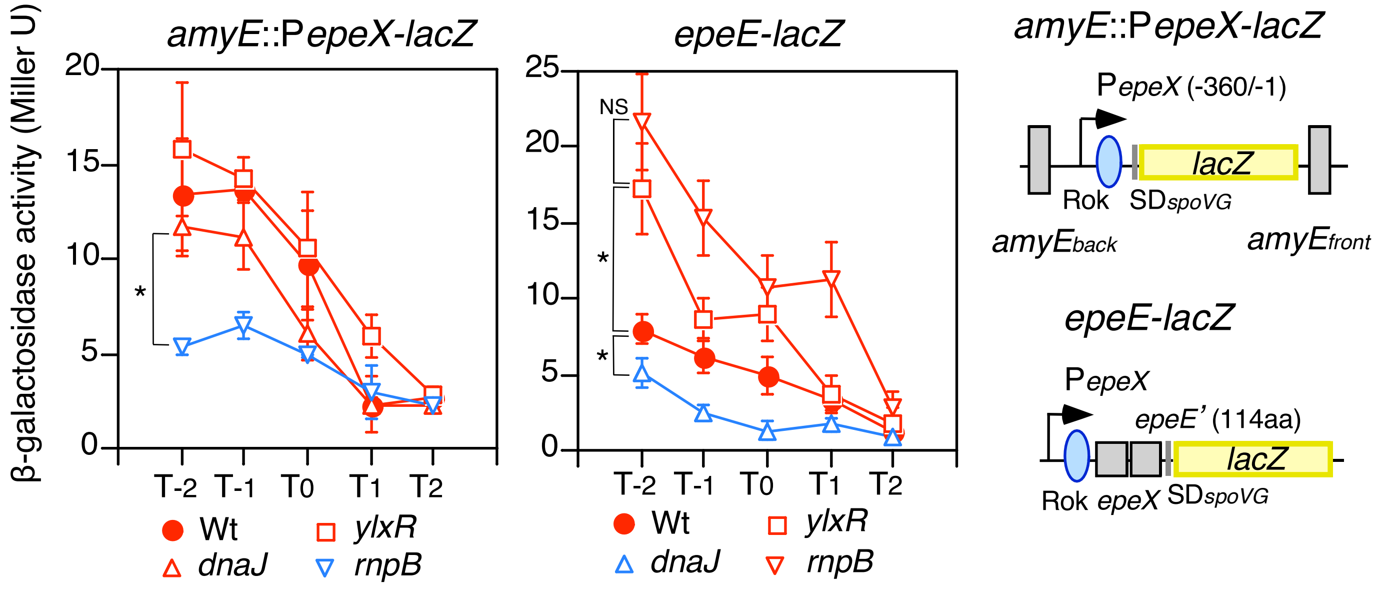

Fig. S3. Expression of P*argGH, besA, and epeXE*.

Fig S4. Y2H analysis and the expression of *spoIVCA* and *nupG*. (A) Y2H results using full-length *dnaK* and *grpE* as bait protein. Genes in bald letters show those gene products decreased in the *dnaJ* mutant in western blot analyses. (**B**) Western blots of SpoIVCA-FLAG (OAM1210), NupG-FLAG (OAM1212). Their derivatives with the *dnaJ* disruption were also analyzed (OAM1211 and OAM1213). (**C**) Schematic representations of transcriptional fusion structure. Box and bent arrows show open reading frame and promoter, respectively. Number in parenthesis indicate promoter region relative to the translation start site. "aa", amino acid. (**D**) β-Galactosidase activities are shown in Miller units. Data represents means ± standard deviations of three independent experiments. The *x*-axis represents the growth time in h relative to the end of vegetative growth (T0). Cells were grown in SM. Substrates CPRG and ONPG were used for *spoIVCA* and *nupG*, respectively. i) OAM1200, wild type; OAM1201, *dnaJ*. ii) JH642 (ϕCMIVCA), wild type; OAM1199, *ylxR*(*rnpM*); OAM1998, *dnaJ*. iii) OAM1204, wild type; OAM1205, *dnaJ*. iv) YXJAd, wild type; OAM1203, *ylxR*(*rnpM*); OAM1202, *dnaJ*. (**E**) Sporulation gene fusions in the *dnaJ* background. The numbers denote means of the peak values (samples were taken at five points) from three independent experiments and standard deviations are in the parenthesis. *spoIID-lacZ* (ASK216, wild type; OAM1214, *dnaJ*), *sspE-lacZ* (168::pPSP918, wild type; OAM1215, *dnaJ*), *cotA-lacZ* (ASK218, wild type; OAM1216, *dnaJ*), *cotD-lacZ* (OAM1218, wild type; OAM1219, *dnaJ*). **(F)** Sporulation test after 24 hours of incubation at 37°C. Wild type 168 cells and *dnaJ* OAM1124 cells were used. Left, phase contrast microscopic images of cells. Right, viable cell numbers before and after heat treatment. The plating was performed in triplicate and each number of colonies is shown.

A

Bait :	Prey
dnaK :	*gltR, smc,* ***spoIVCA****, tenA, yceF,* ***thiW****,* ***nupG****, ypbH, dgkB, nadF, yqeI, ftsE, ftsX, tsaC*
grpE :	*citM, clpQ,* ***spoIVCA****, dhaS, gltR, wapA,* ***nupG****, ykoD, yvbX*

B

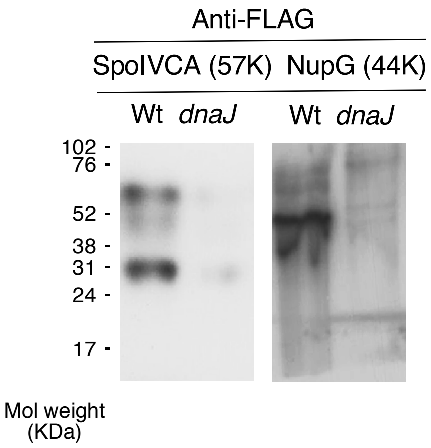

C

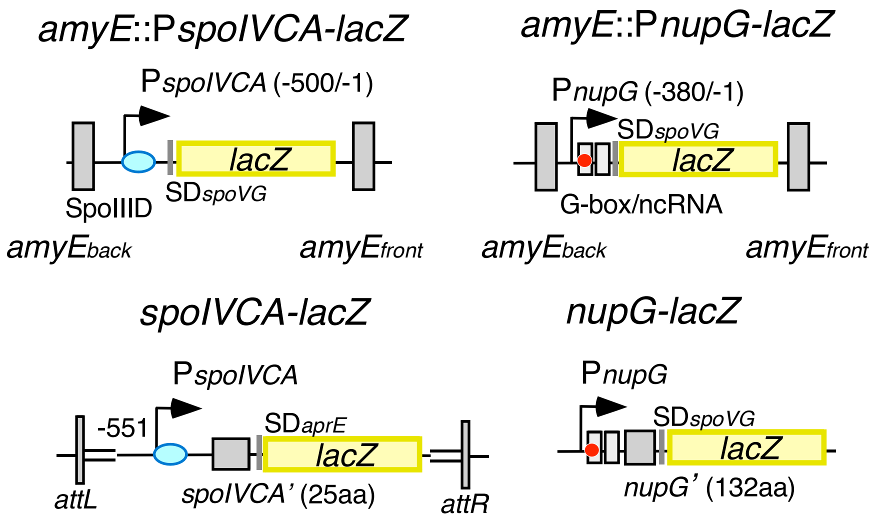

D

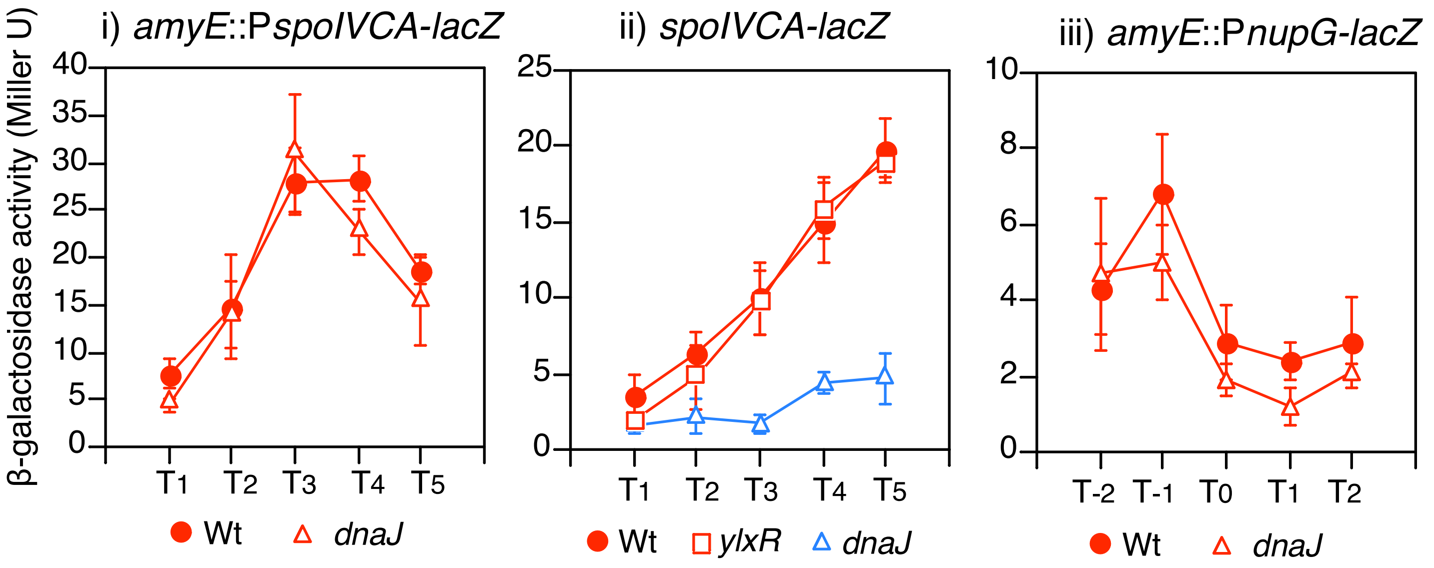

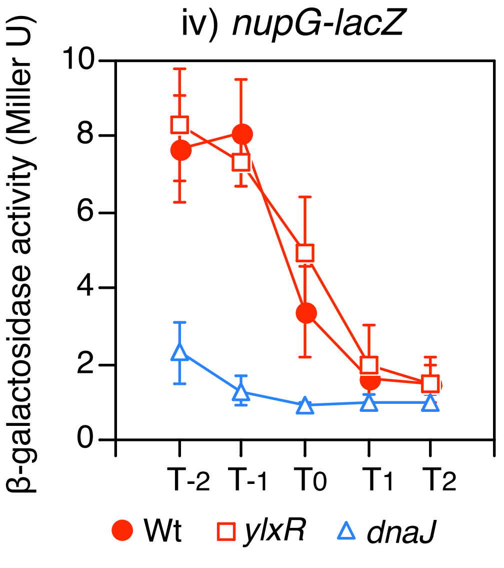

E

Gene fusion	Regulator	β-Gal activity (SD) Miller unit	
		Wt	*dnaJ*
spoIID-lacZ	SigG SpoVT YfhP	41 (3)	37 (5)
sspE-lacZ	SigE SpoIIID Spo0A	29 (3)	32 (4)
cotA-lacZ	SigK GerE	7 (1)	1 (0)
cotD-lacZ	SigK SpoIIID GerE	14 (2)	1 (0)

Fig. S4. Y2H analysis and the expression of *spoIVCA* and *nupG* (continue).

F

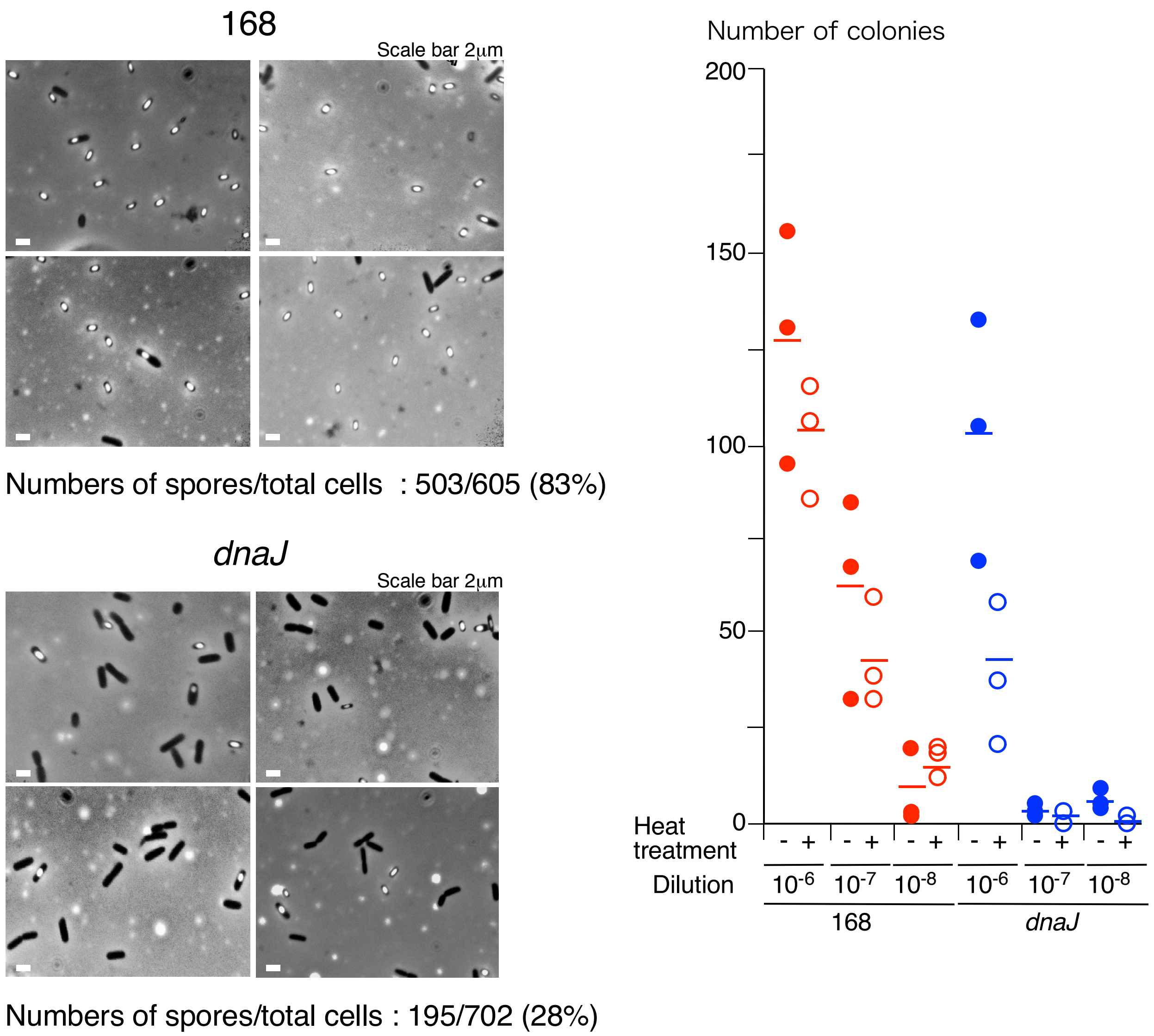

Fig. S4. Y2H analysis and the expression of *spoIVCA* and *nupG*.

Fig. S5. The model proposed for the effects of *rnpB*-depletion by Trinquier *et al*. Left, cascade of stringent response caused by *rnpB*-depletion (24). Right, hypothetic model by Trinquier et al (25). Green ovals show ribosome subunits.

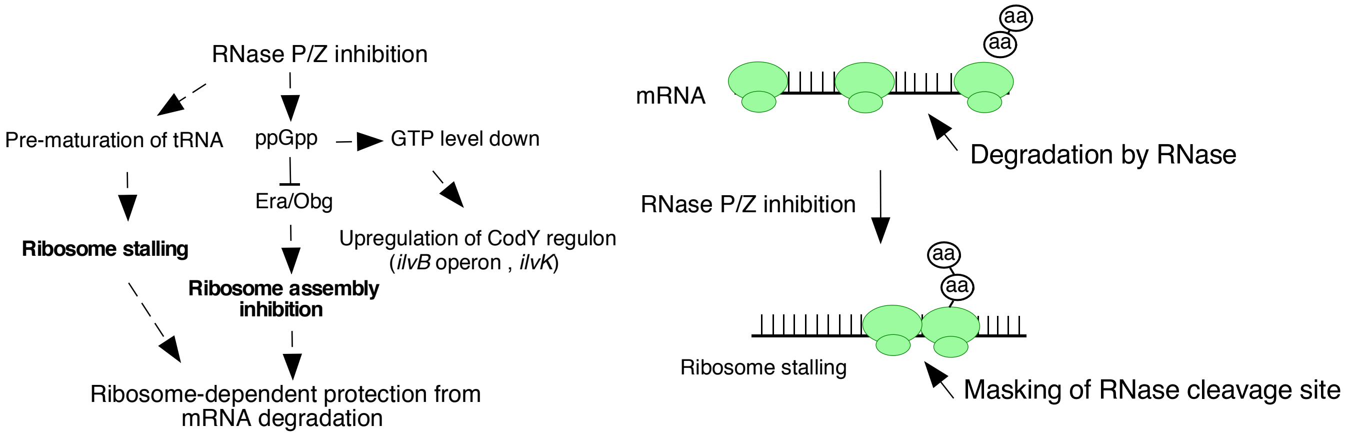

Fig. S5. The model proposed for the effects of *rnpB*-depletion by Trinquier *et al*.
